# The PERK Branch of the Unfolded Protein Response Safeguards Protein Homeostasis and Mesendoderm Specification of Human Pluripotent Stem Cells

**DOI:** 10.1002/advs.202303799

**Published:** 2023-10-27

**Authors:** Fang Liu, Zhun Liu, Weisheng Cheng, Qingquan Zhao, Xinyu Zhang, He Zhang, Miao Yu, He Xu, Yichen Gao, Qianrui Jiang, Guojun Shi, Likun Wang, Shanshan Gu, Jia Wang, Nan Cao, Zhongyan Chen

**Affiliations:** ^1^ Advanced Medical Technology Center Zhongshan School of Medicine and the First Affiliated Hospital Sun Yat‐Sen University Guangzhou 510080 P. R. China; ^2^ Key Laboratory for Stem Cells and Tissue Engineering Sun Yat‐Sen University Ministry of Education Guangzhou 510080 P. R. China; ^3^ Department of Clinical Laboratory The First Affiliated Hospital of Anhui Medical University Hefei 230022 P. R. China; ^4^ Prenatal Diagnosis Center Department of Obstetrics and Gynecology The First Affiliated Hospital of Anhui Medical University Hefei 230022 P. R. China; ^5^ Department of Medical Informatics Zhongshan School of Medicine Sun Yat‐Sen University Guangzhou 510080 P. R. China; ^6^ Guangzhou Municipal Key Laboratory of Mechanistic and Translational Obesity Research Guangdong Provincial Key Laboratory of Diabetology The Third Affiliated Hospital of Sun Yat‐Sen University Guangdong 510080 P. R. China; ^7^ National Laboratory of Biomacromolecules CAS Center for Excellence in Biomacromolecules Institute of Biophysics Chinese Academy of Sciences Beijing 100101 P. R. China; ^8^ College of Life Sciences University of Chinese Academy of Sciences Beijing 100049 P. R. China; ^9^ School of Health and Life Sciences University of Health and Rehabilitation Sciences Shandong 266071 China

**Keywords:** human pluripotent stem cells, mesendoderm specification, PERK, proteostasis, unfolded protein response

## Abstract

Cardiac development involves large‐scale rearrangements of the proteome. How the developing cardiac cells maintain the integrity of the proteome during the rapid lineage transition remains unclear. Here it is shown that proteotoxic stress visualized by the misfolded and/or aggregated proteins appears during early cardiac differentiation of human pluripotent stem cells and is resolved by activation of the PERK branch of unfolded protein response (UPR). PERK depletion increases misfolded and/or aggregated protein accumulation, leading to pluripotency exit defect and impaired mesendoderm specification of human pluripotent stem cells. Mechanistically, it is found that PERK safeguards mesendoderm specification through its conserved downstream effector ATF4, which subsequently activates a novel transcriptional target WARS1, to cope with the differentiation‐induced proteotoxic stress. The results indicate that protein quality control represents a previously unrecognized core component of the cardiogenic regulatory network. Broadly, these findings provide a framework for understanding how UPR is integrated into the developmental program by activating the PERK‐ATF4‐WARS1 axis.

## Graphical abstract

The development of the heart involves extensive rearrangements of the proteome. However, it remains unclear how developing cardiac cells uphold proteome integrity amidst swift lineage transition. This study highlights the crucial role played by the PERK branch of unfolded protein response to resolve differentiation‐induced proteotoxic stress, thereby safeguarding initial cardiac specification in human pluripotent stem cells.

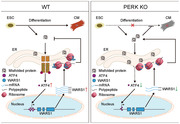

## 1 Introduction

The heart is the first organ to form and function during human embryogenesis. Heart development involves the sequential specification of the pluripotent epiblast cells into the mesendoderm, mesoderm, cardiac progenitor cells (CPCs), and differentiated cardiac cell types, including cardiomyocytes (CMs).^[^ ^1^ ^]^ Cardiac differentiation of human pluripotent stem cells (hPSCs), including human embryonic stem cells (hESCs) and human induced pluripotent stem cells (hiPSCs), reproduces putative mechanisms of the aforementioned sequence of events, emerging as an invaluable model to study both human heart development and pathobiology of inherited and acquired heart disease.^[^ ^2^ ^]^

During the rapid cell fate transitions in cardiogenesis, the acquisition of cellular identities involves robust and dynamic modulation of transcription of a vast number of genes. It not only rewrites the transcriptome to determine a particular cellular state but also triggers a myriad of changes in the composition of the proteome. To maintain the integrity and quality of the proteome, hereafter referred to as protein homeostasis (or proteostasis), cells that undergo cardiac commitment must acquire a considerable capacity for protein synthesis and also the machinery for precisely folding of newly synthesized proteins. Accumulated evidence indicates that proteostasis determines successful cell function, development, and organismal viability,^[^ ^3^ ^]^ whereas its dysregulation leads to misfolded protein aggregation, closely correlating with many developmental defects and degenerative diseases.^[^ ^4^ ^]^ Although the identity and function of many of the lineage‐defining core transcriptional networks, as well as the epigenetic mechanisms that shape them are well‐characterized,^[^ ^5^ ^]^ quality control mechanisms that ensure proteostatic health during the dynamic cardiac developmental transitions remain poorly understood, especially in humans.

Human proteostasis is regulated by a complex network consisting of approximately 2700 components that coordinate protein synthesis, folding, disaggregation, and degradation.^[^ ^6^ ^]^ This proteostasis network includes a diverse collection of macromolecular machines such as chaperones and folding enzymes that operate in diverse ways to maintain proteome integrity. The endoplasmic reticulum (ER) serves as a primary organelle coordinating diverse cellular processes essential for protein folding and assembling. If a cell suddenly needs to make a large number of new proteins, it can overwhelm the ER and unfolded proteins may accumulate, causing ER stress and triggering subsequent activation of a series of complementary adaptive mechanisms to cope with protein‐folding alterations, known as the unfolded protein response (UPR).^[^ ^7^ ^]^ UPR orchestrates the recovery of ER function, enabling the cell to either restore protein homeostasis or initiate programmed death when the ER stress is prolonged. The UPR machinery encompasses three key pathways: activated downstream of the ER stress sensors protein kinase RNA‐like ER kinase (PERK), inositol‐requiring protein‐1α (IRE1α), and activating transcription factor‐6 (ATF6).^[^ ^7a^ ^]^ The activated UPR sensors initiate three branches of signaling transduction, leading to down‐regulation of protein translation, generation of chaperone proteins, and expression of genes that restore the protein folding capacity in the ER. Whether and how the UPR program integrates with human cardiogenesis remains unexplored.

In the present study, we assess the proteostasis control mechanism in cardiac commitment of hPSCs. We hypothesize that the UPR is activated during the lineage transition to cope with the differentiation‐induced protein synthesis stress and that this activation is important for proteome integrity maintenance and cardiac commitment of hPSCs.

## 2 Results

### 2.1 Accumulation of Insoluble Protein Aggregates and Dynamic Activation of Specific Arms of the UPR during Cardiac Differentiation of hPSCs

**Figure 1:**
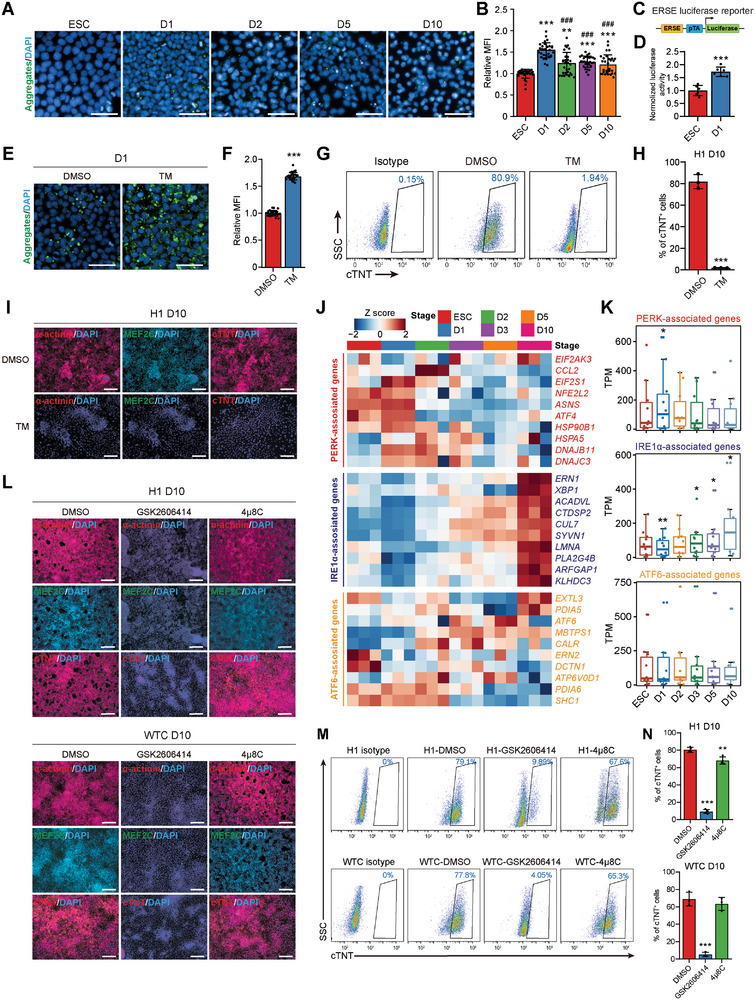
Transient unfolded protein accumulation accompanied by the activation of PERK at the early stage of CM differentiation. A) Representative immunofluorescence analyses of cells at each stage of CM differentiation of H1 hESCs stained with Proteostat (green, protein aggregates). D，differentiation day. Scale bars, 50 µm. B) Quantitative mean fluorescence intensity (MFI) of the protein aggregates normalized to the cell number in samples in (A). *n* = 3 biologically independent experiments, ten fields of view per experiment. ^***^ *P*<0.001 versus ESC; ^###^ *P* < 0.001 versus D1. C) Schematic of the ER stress element (ERSE) luciferase reporter construct. D) Quantitative analyses of ERSE‐luciferase activity in ESC and D1 cells. *n* = 6 biologically independent experiments. ^***^ *P*<0.001 versus ESC. E,F) Representative E) and quantitative F) immunofluorescence analyses of the protein aggregates in D1 cells treated with DMSO or tunicamycin (TM) during differentiation. *n* = 3 biologically independent experiments, 10 fields of view per experiment. ^***^ *P*<0.001 versus DMSO. Scale bars, 50 µm. G,H) Representative G) and quantitative H) flow cytometric analyses of cTNT+ cells in D10 cultures treated with DMSO or TM during differentiation. *n* = 3 biologically independent experiments. ^***^ *P*<0.001 versus DMSO. I) Immunofluorescence analyses of CM markers α‐actinin, MEF2C, and cTNT in D10 cultures treated with DMSO or TM during differentiation. Scale bars, 200 µm. J) Heatmap showing the expression levels of PERK‐, IRE1α‐, and ATF6‐associated genes during CM differentiation of H1 hESCs revealed by RNA‐seq. *n* = 3 biologically independent experiments. K) Expression levels of PERK‐, IRE1α‐, and ATF6‐associated genes during CM differentiation of H1 hESCs revealed by RNA‐seq. ^*^ *P*<0.05, ^**^ *P*<0.01 versus ESCs. L) Immunofluorescence analyses of CM markers α‐actinin, MEF2C, and cTNT in H1 hESC‐ and WTC hiPSC‐derived D10 cultures treated with DMSO, GSK2606414 (PERK inhibitor), or 4µ8C (IRE1α inhibitor) during differentiation. Scale bars, 200 µm. M,N) Representative M) and quantitative N) flow cytometric analyses of cTNT^+^ cells in H1 hESC‐ and WTC hiPSC‐derived D10 cultures treated with DMSO, GSK2606414, or 4µ8C during differentiation. *n* = 4 (H1) or 3 (WTC) biologically independent experiments. ^**^ *P*<0.01, ^***^ *P*<0.001 versus DMSO. Data represent mean ± SD. Statistical significance was determined by one‐way ANOVA with a post‐hoc Tukey test B,N), unpaired two‐tailed t‐test D, F, and H), and Wilcoxon test K).

To study how proteostasis is regulated in cardiac development, we employed direct differentiation of hPSCs to CMs as a model system of human cardiomyogenesis, using a fully chemically defined protocol^[^ ^8^ ^]^ (Figure S1A, Supporting Information) that efficiently generated monolayers of CMs with a >90% purity (Figure S1B, Supporting Information). Differentiated CMs had well‐organized sarcomeres (Figure S1C, Supporting Information) and exhibited robust staining of CM‐specific markers α‐actinin and cTNT (Figure S1D, Supporting Information). To determine whether cells face the burden of protein synthesis overload during the rapid lineage transitions in cardiac differentiation, we captured cell samples at time points corresponding to stage‐specific transitions in cell state including pluripotency (differentiation day (D) 0), mesendoderm (D1), mesoderm (D2), CPCs (D5), and CMs (D10), and stained them with Proteostat dye, which becomes highly fluorescent upon binding to the misfolded and/or aggregated proteins.^[^ ^9^ ^]^ We observed a robust increase of Proteostat staining in mesendoderm cells at D1, which decreased from differentiation D2 in both H1 hESC and WTC hiPSC^[^ ^10^ ^]^ lines (**Figure** **1**A,B; Figure S2A,B, Supporting Information). Consistently, ER stress element (ERSE) reporter assay^[^ ^11^ ^]^ revealed the increased ER stress in D1 cells compared to the undifferentiated hESCs (Figure 1C,D).

To confirm the fidelity of the Proteostat dye staining, we performed immunofluorescent staining analysis and found that the Proteostat dye signals are positively correlated with P62 (Figure S2C,D, Supporting Information) and ubiquitin (Figure S2E,F, Supporting Information) staining at the single cell level, suggesting that the Proteostat^+^ aggregates are ubiquitinated targets undergoing lysosome‐mediated degradation. We also co‐stained the Proteostat dye with apoptotic and necrotic markers and found no overlap, indicating that the Proteostat dye signals are not associated with cell death (Figure S2G–I, Supporting Information). These results confirm that the Proteostat^+^ structures are misfolded and/or aggregated proteins.

To illuminate whether these aggregates affect cardiac differentiation, we treated the differentiating cells with tunicamycin (TM), an N‐glycosylation inhibitor that causes accumulation of the unfolded proteins in the ER.^[^ ^12^ ^]^ TM‐treatment led to markedly increased Proteostat staining in mesendoderm cells (Figure 1E,F) and a total failure of CM differentiation (Figure 1G–I), suggesting that protein homeostasis, which is critical for cardiogenesis of hPSCs, is transiently disturbed after the initiation of cardiac differentiation and partially restored after mesodermal specification.

Because of the pivotal role of UPR in alleviating misfolded protein accumulation‐induced ER stress, we examined whether the three major UPR pathways, including PERK, IRE1α, and ATF6, are activated during cardiac lineage commitment. We performed RNA‐sequencing (RNA‐seq) at each stage of CM differentiation and generated a time‐course gene expression profile using a wide range of known UPR genes. We found that the expression levels of PERK‐associated genes were transiently elevated in the early stages of differentiation (D1 and D2) and decreased thereafter, whereas a large panel of IRE1α‐associated genes were dominantly expressed in the CM stage (Figure 1J). Additionally, the expression of ATF6‐associated genes was not consistently altered during differentiation (Figure 1K). These results suggest that the transient activation of PERK or IRE1α branch of UPR may regulate early or late stage of cardiac differentiation, respectively, via affecting protein homeostasis maintenance.

### 2.2 PERK is Required for CM Differentiation

To verify which branch of UPR plays a physiological role in cardiomyogenesis, we treated the H1 hESCs and WTC hiPSCs that underwent cardiac differentiation with small molecule inhibitors of PERK (GSK2606414, 1 µm), IRE1α (4µ8C, 3 µm), or ATF6 (Ceapin‐A7, 9 µm), as well as an activator of ATF6 (AA147, 10 µm), respectively. We found that the cardiomyogenic potential of both hESCs and hiPSCs was only severely impaired in GSK2606414‐ or Ceapin‐A7‐treated cells (Figure 1L–N; Figure S3A–E, Supporting Information), indicating that both PERK and ATF6 are critical for cardiac commitment. Interestingly, we found that only pharmacological inhibition of PERK, but not ATF6, led to the accumulation of misfolded and/or protein aggregates in D1 cells (Figure S3F,G, Supporting Information), suggesting that PERK may protect the cells from differentiation‐induced ER stress. Therefore, PERK was evaluated for the remainder of this study.

**Figure 2:**
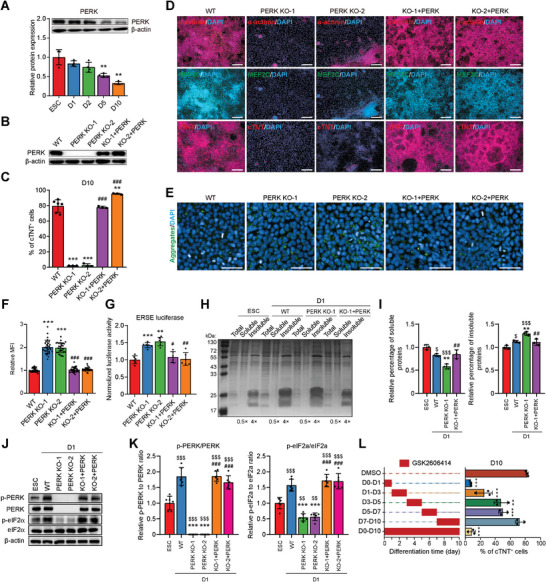
PERK depletion impairs CMs differentiation. A) Representative and quantitative immunoblot analysis of PERK during CM differentiation of hESCs. β‐actin was used as a loading control. *n* = 4 biologically independent experiments. ^**^ *P*<0.01 versus ESC. B) Immunoblot analysis of PERK in wildtype (WT) hESCs, PERK KO clone 1 (PERK KO‐1) and 2 (PERK KO‐2), and PERK re‐expressed hESC clones. C) Quantitative flow cytometric analysis of cTNT^+^ cells in D10 cultures differentiated from WT, PERK KO, and PERK re‐expressed hESCs. *n* = 6 (WT) or 3 (other groups) biologically independent experiments. ^**^ *P*<0.01, ^***^ *P*<0.001 versus WT; ^###^ *P*<0.001 versus the corresponding PERK KO clone. D) Immunofluorescence analysis of CM markers in D10 cultures differentiated from WT, PERK KO, and PERK re‐expressed hESCs. Scale bars, 200 µm. E,F) Representative E) and quantitative F) immunofluorescence analysis of the protein aggregates in D1 cultures differentiated from WT, PERK KO, and PERK re‐expressed hESCs. *n* = 3 biologically independent experiments, ten fields of view per experiment. Scale bars, 50 µm. ^***^ *P*<0.001 versus WT; ^###^ *P*<0.001 versus the corresponding PERK KO clone. G) Quantitative analysis of ERSE‐luciferase activity in D1 cultures differentiated from WT, PERK KO, and PERK re‐expressed hESCs. *n* = 6 biologically independent experiments. ^**^ *P*<0.01, ^***^ *P*<0.001 versus WT; ^#^ *P*<0.05, ^##^ *P*<0.01 versus the corresponding PERK KO clone. H,I) Representative H) and quantitative I) SDS‐PAGE gel stained with Coomassie brilliant blue for detection of protein in the total, soluble, and insoluble fractions of D1 cultures differentiated from WT, PERK KO‐1, and PERK re‐expressed hESCs. *n* = 3 biologically independent experiments. ^$^ *P*<0.05, ^$$$^ *P*<0.001 versus WT ESC; ^**^ *P*<0.01 versus WT D1; ^##^ *P*<0.01 versus PERK KO‐1 at D1. J,K) Representative J) and quantitative K) immunoblot analysis of p‐PERK, PERK, p‐eIF2α, and eIF2α in D1 cultures differentiated from WT, PERK KO, or PERK re‐expressed H1 hESCs. β‐actin was used as a loading control. *n* = 6 biologically independent experiments. ^$$^ *P*<0.01, ^$$$^ *P*<0.001 versus WT ESC; ^***^ *P*<0.001 versus WT D1; ^###^ *P*<0.001 versus the corresponding PERK KO clone at D1. L) Effect of stage‐specific treatments of GSK2606414 on cardiac differentiation. Left panel, schematic diagram of the time windows of GSK2606414‐treatment; right panels, the corresponding percentage of cTNT^+^ cells detected by flow cytometry. *n* = 3 biologically independent experiments. ^**^ *P*<0.01, ^***^ *P*<0.001 versus DMSO. Data represent mean ± SD. Statistical significance was determined by one‐way ANOVA with a post‐hoc Tukey test.

To identify the expression pattern of PERK during cardiac differentiation, we measured its protein expression by immunoblot analysis. Consistent with the expression pattern of PERK‐associated genes (Figure 1K), PERK was enriched in hESCs at the early stage of differentiation, and gradually down‐regulated after further specification (**Figure** **2**A). To investigate the role of PERK in cardiac differentiation, we generated a PERK knockout (KO) embryonic stem cell line from hESCs using the CRISPR/Cas9 system. Guide RNA was targeted to the first exon of PERK, and CRISPR/Cas9‐mediated DNA deletion leading to frameshift mutation was introduced into both alleles. DNA sequencing and immunoblot analysis confirmed two clonal of PERK KO hESC lines, PERK KO‐1 and PERK KO‐2 (Figure S4A, Supporting Information; Figure 2B). Bioinformatic prediction^[^ ^13^ ^]^ followed by Sanger sequencing revealed no off‐target editing of the top five potential sites (Figure S4B, Supporting Information). In addition, by using a doxycycline‐inducible lentiviral system, PERK expression could be completely restored in both PERK KO clones by doxycycline addition for 24 h (Figure 2B).

During passaging, both PERK KO hESC lines retained a stable growth rate and undifferentiated morphology. They also exhibited high alkaline phosphatase activity (Figure S4C, Supporting Information) and uniform expression of the pluripotent markers, including NANOG, OCT4, SOX2, TRA‐1‐81, and SSEA4 (Figure S4D–F, Supporting Information), as well as the proliferative marker Ki67 (Figure S4G, Supporting Information). These data demonstrate that PERK is dispensable for self‐renewal of undifferentiated hESCs. In striking contrast, the PERK KO clones almost completely lost the capacity to generate CMs upon cardiac induction in comparison with the isogenic wild‐type (WT) cells, whereas re‐expression of PERK for only 24 h rescued the cardiomyogenic defects in both KO cell lines (Figure 2C,D; Figure S5A–C, Supporting Information). We also generated PERK KO cell line using WTC hiPSCs as a replicate (Figure S6A, Supporting Information). As expected, PERK KO in hiPSCs resulted in a similar phenotype as in hESCs (Figure S6B–D, Supporting Information).

To assess whether the cardiogenic defect caused by PERK KO is method‐dependent, we used an alternative cardiac differentiation protocol^[^ ^14^ ^]^ and observed similar results (Figure S7A–D, Supporting Information). Since the majority of CMs generated by modulating the WNT signaling are ventricular‐like,^[^ ^14^, ^15^ ^]^ we asked whether atrial CM differentiation may have a different requirement of PERK. To test it, we differentiated WT and PERK KO hESCs into atrial‐like CMs using a published protocol^[^ ^16^ ^]^ and found PERK KO similarly prevented the generation of atrial‐like CMs (Figure S8A–D, Supporting Information). To further investigate this question, we employed a recently developed self‐organizing human cardioid method^[^ ^17^ ^]^ which can pattern and morph into chamber‐like structures and best to date recapitulate the in vivo heart lineage architecture to model human development in dishes. Cardioids derived from the wildtype hESCs could rapidly and reproducibly self‐assemble into 3D sphere and formed beating cavity‐containing structures positive for cardiomyocyte markers cTNT and MEF2C 7.5 days post differentiation (Figure S9A,B, Supporting Information). In contrast, cardioids derived from the PERK KO hESCs failed to recapitulate this self‐assembly and specification program and were absent for CM formation (Figure S9C, Supporting Information), whereas re‐expression of PERK rescued the cardioid formation defects in both KO cell lines. Together, these results suggest that PERK plays a pivotal role in cardiac differentiation of hPSCs via affecting proteostasis during early mesendoderm specification.

To test this hypothesis, we further examined the presence of protein aggregates by Proteostat staining in WT, PERK KO, and PERK re‐expression cell lines upon induction to mesendoderm (at D1). As expected, insoluble protein aggregates became visible in WT hESC‐ and hiPSC‐derived mesendoderm cells (at D1) and were dramatically accumulated after PERK KO when compared to the WT control (Figure 2E,F; Figure S6E,F, Supporting Information), indicating a more severely disturbed protein homeostasis after PERK ablation. Notably, this phenotype induced by PERK deficiency could be completely restored by re‐introduction of PERK (Figure 2E,F; Figure S6E,F, Supporting Information). To further validate the accumulation of protein aggregates in mesendoderm cells, we adopted an alternative mesodermal differentiation protocol,^[^ ^18^ ^]^ which sequentially generates mesendoderm and lateral mesoderm by using Activin A and BMP4 as the core inducers (Figure S10A,B, Supporting Information). We observed a similar accumulation of the Proteostat^+^ protein aggregates after differentiation, peaked in D1 mesendoderm cells (Figure S10C,D, Supporting Information). Once again, PERK KO hESCs that underwent lateral mesoderm specification generated more protein aggregates than their WT counterparts at both D1 (Figure S10E,F, Supporting Information) and D2 (Figure S10G,H, Supporting Information), a phenomenon that could be reversed by re‐introduction of PERK. Consistently, ERSE reporter assay revealed a significantly elevated ER stress level in PERK KO cells, which could also be reversed by re‐introduction of PERK (Figure 2G).

To further confirm the presence of protein aggregates after PERK ablation, we performed biochemical fractionation of lysates from WT, PERK KO, and PERK re‐expressed mesendoderm cells at D1 to enrich protein aggregates.^[^ ^19^ ^]^ As expected, we found a visible decrease in the soluble protein fraction accompanied by an increase in the insoluble protein fraction in WT D1 cells when compared to the undifferentiated hESCs (Figure 2H,I). The presence of proteins at a variety of molecular weights in the insoluble fraction suggests that this aggregation appears to affect many proteins. Notably, we confirmed that PERK KO cells at D1 contained significantly more insoluble protein aggregates and less soluble proteins than their WT counterparts, which was reversible by PERK re‐expression (Figure 2H,I). Consistently, we confirmed a significant increase of the total ubiquitin levels in WT D1 cells compared to the undifferentiated ESCs by immunoblot analysis, and the ubiquitin level in D1 cells further increased after PERK KO (Figure S11, Supporting Information). Once again, PERK KO‐induced elevation of ubiquitin could be restored by PERK re‐introduction (Figure S11, Supporting Information). Consistent with the observation that protein aggregates of D1 mesendodermal cells are not associated with cell death (Figure S2G‐I, Supporting Information), WT and PERK KO cells exhibited comparable cell survival and growth rate (Figure S12, Supporting Information), indicating that PERK KO‐induced cardiogenic defect is not associated with either cell death or cell cycle arrest.

As a kinase, PERK is activated by phosphorylation (p‐PERK). P‐PERK further phosphorylates the alpha subunit of eukaryotic initiation factor 2 (eIF2α), leading to the transient attenuation of global protein synthesis.^[^ ^20^ ^]^ By immunoblot analysis, we found increased phosphorylation of both PERK and eIF2α in D1 mesendoderm cells compared to the undifferentiated hESCs, whereas PERK KO resulted in a sharp decrease of phosphorylated eIF2α, which could be restored by re‐introduction of PERK (Figure 2J,K).

We next accessed the period during which PERK is required by adding GSK2606414 at various frames over the course of cardiac differentiation. GSK2606414 applied at the window in which mesendoderm was formed (D0‐1) was equivalently effective to GSK2606414‐treatment during the entire differentiation period, producing the anticipated robust decrease in cTNT^+^ CMs (Figure 2L; Figure S13A, Supporting Information). In contrast, pharmacological inhibition of PERK during mesoderm, CPC, or CM formation stages had less or no effects (Figure 2L). This result was further supported by oligo siRNAs‐mediated transient PERK knockdown (KD) experiments, in which PERK KD at D2 resulted in similar reduction in cTNT^+^ CMs formation (Figure S13B–D, Supporting Information), with a level similar to GSK2606414 administration at this window. To further confirm this conclusion, we examined the expression of mesendoderm marker Brachyury in WT and PERK KO cardioids at D1.5 and found that PERK KO similarly prevented the generation of mesendoderm in 3D culture (Figure S9D, Supporting Information). To explore whether PERK is required for maintaining CM character and function, we generated CMs with advanced maturity according to published methods^[^ ^20^, ^21^ ^]^ (Figure S14A–E, Supporting Information) and treated them with GSK2606414. We found that PERK inhibition by GSK2606414 did not affect the proliferation (Figure S14F,G, Supporting Information), survival (Figure S14H,I, Supporting Information), or calcium handling properties of CMs (Figure S14J,K, Supporting Information), indicating that PERK has little impact on differentiated CMs. Together, these results demonstrate that PERK expression is necessary for cardiogenesis of hESCs, possibly through affecting mesendoderm lineage commitment, the earliest step of cardiac differentiation.

### 2.3 Deletion of PERK Disrupts Gene Networks in Pluripotency Exit and Mesendoderm Commitment

**Figure 3:**
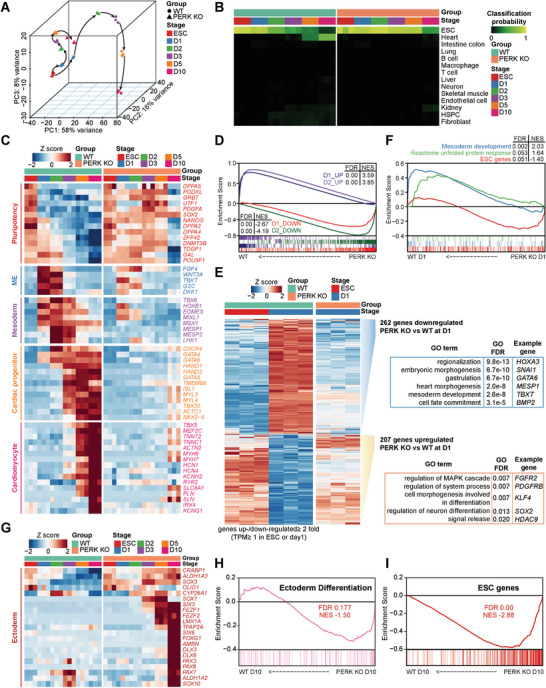
PERK KO hESCs lose transcriptional signatures of developing CMs. A) Principal‐component analysis of the global gene expression profile across all samples during CM differentiation of WT and PERK KO hESCs revealed by RNA‐seq. B) Cell classification heatmap of all tested samples generated by CellNet analysis. Higher classification scores indicate a higher probability that a query sample (vertical axis) resembles the training sample (horizontal axis). C) Expression of marker genes of each stage of CM differentiation detected by RNA‐seq. D) Gene set enrichment analysis (GSEA) of the RNA‐seq data from WT and PERK KO hESCs at D1 and D2. Sets of genes that significantly up‐ or down‐regulated in WT mesendoderm (D1) and mesoderm (D2) cultures compared with the WT undifferentiated ESCs, respectively, are used. E) Heatmap showing up‐ and down‐regulated genes in WT and PERK KO mesendoderm cells (D1), as compared to WT ESC. Gene ontology (GO) analysis of genes deregulated by at least twofold in PERK KO mesendoderm cells as compared to WT mesendoderm cells are presented in the right panel. F) GSEA of the RNA‐seq data from WT and PERK KO mesendoderm cells (D1). Gene sets from the GO term “mesoderm development”, the Reactome Pathways term “unfolded protein response”, and the ESC‐enriched genes^[^ ^76^ ^]^ are used. G) Expression of ectoderm marker genes detected by RNA‐seq. H,I) GSEA of the RNA‐seq data from WT and PERK KO cells at D10. Gene sets from the GO term “ectoderm differentiation” and the ESC‐enriched genes^[^ ^76^ ^]^ are used.

To explore the molecular mechanisms by which PERK regulates mesendoderm commitment and subsequent cardiac specification, we compared the transcriptomes of the cultures at each stage of CM differentiation derived from both WT and PERK KO hESCs by RNA‐seq. By principal‐component (PCA) and correlation analysis, we found that the PERK KO hESCs failed to recapitulate the continuous molecular roadmap of human cardiogenesis that was apparent in the WT hESCs. Instead, PERK KO cells exposed to the differentiation‐inducing cues retained similar transcriptional profile to undifferentiated cells and appeared to be “stuck” at the pluripotency exit step, failing to initiate the mesendoderm segregation and subsequent cardiogenic program (**Figure** **3**A; Figure S15A, Supporting Information). To further confirm this observation, we compared the transcriptome of WT and PERK KO cells using the CellNet informatics platform that reconstructs gene regulatory networks and provides a quantitative metric of cell identity by calculating the classification score of each sample.^[^ ^22^ ^]^ Consistently, we found that the classification term “Heart”, which started to emerge in WT hESCs at D5 and became more apparent at D10, was completely absent in PERK KO cells after differentiation (Figure 3B). In contrast, differentiated PERK KO cells were still classified mainly as “ESCs” and scored similar to undifferentiated cells even after being cultured in the CM differentiation condition for up to 5 days (Figure 3B).

Furthermore, by using the ImpluseDE2 tool,^[^ ^23^ ^]^ we divided the differentially expressed genes in WT hESCs that underwent normal CM differentiation into four categories according to their expression patterns. However, such expression patterns were not observed in the PERK KO group (Figure S15B, Supporting Information). Interestingly, in gene ontology (GO) enrichment analysis, we found that genes up‐regulated during normal cardiac differentiation enriched similar sets of terms with the down‐regulated genes in PERK KO cells (Figure S15C, Supporting Information), and vice versa for genes that decreased during cardiac differentiation (Figure S15D, Supporting Information). More specifically, by evaluating a panel of well‐studied genes, we found that the sequential induction of mesendoderm, mesodermal, CPC, and CM genes that are involved in normal cardiogenesis of WT hESCs was dramatically impaired by PERK KO, whereas the expression of pluripotent genes that gradually decrease during WT hESC differentiation retained high in differentiating PERK KO cells (Figure 3C). In aggregate, these results suggest that PERK is a crucial regulator of pluripotency exit and mesendoderm cell generation, required for hESCs to enter the normal cardiogenic program.

To further investigate the PERK‐dependent pluripotency exit and early germ‐layer specification, we more specifically analyzed the gene expression changes in control and PERK KO cells at the mesendoderm (D1) and mesoderm (D2) stages. By gene set enrichment analysis, we observed that the genes that were up‐ or down‐regulated in WT mesendoderm/mesoderm when compared with the ESCs had a totally different expression trend in PERK KO cells (Figure 3D). More precisely, we found 262 and 860 genes to be aberrantly down‐regulated in PERK‐depleted cells at D1 (Figure 3E) and D2 (Figure S16A, Supporting Information), respectively. These genes are involved in embryonic morphogenesis and mesendoderm/mesoderm development (e.g., *TBXT*, *MESP1*, *EOMES*, and *GATA6*) (Figure 3E,F; Figure S16A,B, Supporting Information), suggesting that PERK KO hESCs have lost pluripotency and are not able to fully differentiate. Conversely, a panel of 207 and 1007 genes were abnormally up‐regulated in PERK‐depleted cells at D1 (Figure 3E) and D2 (Figure S16A, Supporting Information), respectively, with a strong enrichment of the gene networks associated with ESC self‐renewal and growth (Figure 3E,F; Figure S16A–C, Supporting Information). As expected, PERK depletion also strongly reduced the expression of UPR‐related genes (Figure 3F; Figure S16D, Supporting Information), suggesting a close correlation between the activation of PERK brunch of UPR and faithful pluripotency exit/mesendoderm commitment of hESCs.

To further elucidate the fate of PERK‐depleted cells, we analyzed the transcriptional signatures of the up‐regulated genes in PERK KO cells at D10 compared with WT cells. We found that genes that are important for ectoderm differentiation, including many known marker transcripts of the neural cells (e.g., *PAX6*, *SIX6*, and *SOX10*), were significantly enriched in PERK KO hESCs that underwent 10 days of cardiac differentiation (Figure 3G,H; Figure S17A, Supporting Information). As expected, pluripotency genes were also up‐regulated in PERK KO cells at D10 (Figure 3I). The retention of pluripotency and presence of neural cells in PERK‐depleted cells were further confirmed by immunofluorescence staining analysis for ESC marker OCT4 and key neural marker PAX6 and SOX1 (Figure S17B, Supporting Information). These data further suggest that PERK KO cells retain pluripotency and differentiate into neural lineage despite being cultured in mesendoderm‐inducing conditions.

### 2.4 PERK is Required for Mesoderm and Endoderm Differentiation

**Figure 4:**
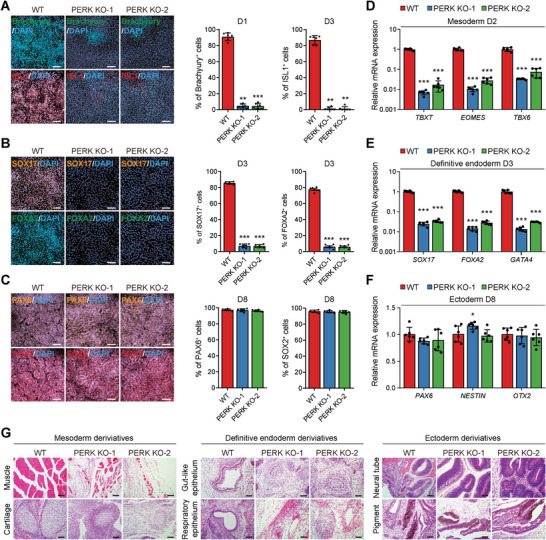
PERK regulates mesoderm and endoderm specification. A–C) Representative (left) and quantitative (right) immunofluorescence analysis of the mesoderm markers Brachyury and ISL1 A), definitive endoderm markers FOXA2 and SOX17 B), and ectoderm markers PAX6 and SOX2 C) in cultures derived from WT and PERK KO hESCs. *n* = 6 biologically independent experiments. Scale bars, 100 µm. ^**^ *P*<0.01, ^***^ *P*<0.001 versus WT. D–F) RT‐qPCR analysis of mesoderm D), definitive endoderm E), and ectoderm F) marker gene expression in cultures derived from WT and PERK KO hESCs. *n* = 6 biologically independent experiments. ^*^ *P*<0.05, ^***^ *P*<0.001 versus WT. G) Hematoxylin and eosin staining of teratoma derived from WT and PERK KO hESCs, showing tissue derivatives of the mesoderm (left), definitive endoderm (middle), or ectoderm (right). Scale bars, 100 µm. Data represent mean ± SD. Statistical significance was determined by one‐way ANOVA with a post‐hoc Tukey test.

Because mesendoderm is the source of both the mesoderm and the definitive endoderm,^[^ ^24^ ^]^ we next examined the impact of PERK KO on mesodermal and endodermal specification of hESCs. Ectoderm‐specific differentiation was also performed as a control. Strikingly, PERK ablation led to >90% decreases in the presence of Brachyury^+^/ISL1^+^ mesodermal cells (**Figure** **4**A) or SOX17^+^/FOXA2^+^ endodermal cells (Figure 4B), as revealed by immunofluorescence staining analysis of cells at D3 of cardiac differentiation and definitive endoderm differentiation. In contrast, induction of PAX6^+^/SOX2^+^ ectodermal cells at D8 of ectoderm differentiation from hESCs was not altered by PERK KO (Figure 4C). This conclusion was further validated by measuring the mRNA expression of three germ‐layer markers, in which key mesodermal and endodermal genes were significantly down‐regulated in PERK KO cells, whereas expression levels of genes important for ectoderm formation retained similar in the presence or absence of PERK (Figure 4D–F).

To further document the differentiation potential of PERK‐deficient hESCs, we performed teratoma assay, the gold standard for proving pluripotency of hPSCs,^[^ ^25^ ^]^ by subcutaneously transplanting both the WT and PERK KO hESCs into the groin of immunodeficient mice. Nine weeks after transplantation, we examined the teratoma tissues for evidence of cellular differentiation. In mice that received WT hESC transplantation, we observed various tissues originating from the mesoderm (e.g., muscle and cartilage), the definitive endoderm (e.g., respiratory and gut‐like epithelium), and the ectoderm (e.g., neural tube and pigment epithelial cells) (Figure 4G). In contrast, derivatives from mesoderm or endoderm were much less frequently observed and appeared to be immature in teratomas formed by the PERK KO hESCs in vivo, when compared with the WT control (Figure 4G). Once again, the formation of ectodermal derivatives was not obviously altered by PERK KO (Figure 4G). Taken together, these data from both the in vitro and in vivo context establish that PERK is determinant for hESCs to differentiate into the definitive endoderm and mesoderm, progenies of the mesendoderm precursors.

### 2.5 ATF4 is the Downstream Target of PERK and Directs Cell Fate toward Mesendoderm

**Figure 5:**
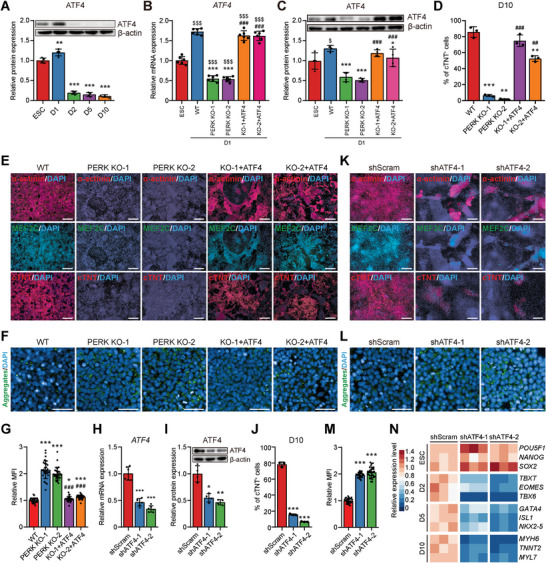
PERK promotes mesendoderm differentiation via activation of ATF4. A) Representative (upper) and quantitative (lower) immunoblot analysis of ATF4 during CM differentiation of hESCs. β‐actin was used as a loading control. *n* = 4 biologically independent experiments. ^**^ *P*<0.01, ^***^ *P*<0.001 versus ESC. B) RT‐qPCR analysis of ATF4 in D1 mesendoderm cells differentiated from WT, PERK KO, and ATF4‐overexpressed PERK KO hESCs. *n* = 6 biologically independent experiments. ^$$$^ *P*<0.001 versus WT ESC; ^***^ *P*<0.001 versus WT D1; ^###^ *P*<0.001 versus the corresponding PERK KO clone at D1. C) Representative (upper) and quantitative (lower) immunoblot analysis of ATF4 in D1 mesendoderm cells differentiated from WT, PERK KO, and ATF4‐overexpressed PERK KO hESCs. β‐actin was used as a loading control. *n* = 4 biologically independent experiments. $*P*<0.05 versus WT ESC; ^*^ *P*<0.05, ^***^ *P*<0.001 versus WT D1; ^###^ *P*<0.001 versus the corresponding PERK KO clone at D1. D) Quantitative flow cytometric analysis of cTNT+ cells in D10 cultures differentiated from WT, PERK KO, and ATF4‐overexpressed PERK KO hESCs. *n* = 3 biologically independent experiments. ^**^ *P*<0.01, ^***^ *P*<0.001 versus WT; ^##^ *P*<0.01, ^###^ *P*<0.001 versus the corresponding PERK KO clone. E) Immunofluorescence analysis of CM markers in D10 cultures differentiated from WT, PERK KO, and ATF4‐overexpressed PERK KO hESCs. Scale bars, 200 µm. F,G) Representative F) and quantitative G) immunofluorescence analysis of the protein aggregates in D1 cultures differentiated from WT, PERK KO, and ATF4‐overexpressed PERK KO hESCs. *n* = 3 biologically independent experiments, ten fields of view per experiment. Scale bars, 50 µm. ^*^ *P*<0.05, ^***^ *P*<0.001 versus WT; ^###^ *P*<0.001 versus the corresponding PERK KO clone. H,I) RT‐qPCR H) and immunoblot I) analysis of ATF4 in shScram control and ATF4 KD mesendoderm cells at D1. shATF4‐1 and shATF4‐2 represent two independent ATF4 shRNAs. *n* = 6 and 4 biologically independent experiments for H) and I), respectively. ^*^ *P*<0.05, ^**^ *P*<0.01, ^***^ *P*<0.001 versus shScram. J) Quantitative flow cytometric analysis of cTNT+ cells in D10 cultures differentiated from shScram control and ATF4 KD hESCs. *n* = 3 biologically independent experiments. ^***^ *P*<0.001 versus shScram. K) Immunofluorescence analysis of CM markers in D10 cultures differentiated from shScram control and ATF4 KD hESCs. Scale bars, 200 µm. L,M) Representative L) and quantitative M) immunofluorescence analysis of the protein aggregates in shScram control and ATF4 KD mesendoderm cells at D1. *n* = 3 biologically independent experiments, ten fields of view per experiment. Scale bars, 50 µm. ^***^ *P*<0.001 versus shScram. N) Heatmap showing the relative expression level of marker genes of each differentiation stage in shScram control and ATF4 KD cells, determined by RT‐qPCR. *n* = 3 biologically independent experiments. Data represent mean ± SD. Statistical significance was determined by one‐way ANOVA with a post‐hoc Tukey test.

Next, we investigated the underlying mechanisms of PERK on regulation of its target gene during mesendoderm specification and subsequent CM differentiation. Whereas PERK activation by ER stress leads to global translational attenuation, it paradoxically increases the translation of ATF4,^[^ ^26^ ^]^ a key downstream effector of PERK to relieve protein folding pressure and safeguard ER proteostasis.^[^ ^27^ ^]^ Hence, we explored whether ATF4 is involved in PERK‐regulated cardiac differentiation. The expression level of ATF4 was peak at D1, the mesendoderm stage, then rapidly decreased thereafter (Figure 5A), consisting with the expression pattern of PERK (Figure 2A) and PERK‐regulated genes (Figure 1J). In addition, there were noteworthy reductions in *ATF4* mRNA and protein levels at D1 after PERK KO (**Figure** **5**B,C). To further investigate whether ATF4 is activated by PERK in D1 cells, we constructed a luciferase‐based lentiviral ATF4 reporter as previously reported^[^ ^28^ ^]^ to measure the ATF4 translation rate (Figure S18A, Supporting Information). As expected, increased ATF4 reporter activation was observed at D1 and was fully abolished by PERK KO, whereas PERK re‐expression reactivated ATF4 reporter to the WT level (Figure S18B, Supporting Information). As a stress‐inducible transcription factor, ATF4 translocate into the nucleus to activate the expression of genes involved in relieving ER stress.^[^ ^27a^ ^]^ Consistently, we found an increase of translocation of ATF4 into the nucleus in D1 mesendoderm cells when compared with the hESCs (Figure S18C,D, Supporting Information). Once again, PERK KO completely inhibited ATF4 activation and its nuclear translocation, which could be rescued by re‐introduction of PERK (Figure S18C,D, Supporting Information). In accordance with these observations, the ATF4 target protein GRP78 (encoded by the gene *HSPA5*) exhibited a significant increase in expression (Figure 1J; Figure S18E,F, Supporting Information) and augmented translocation to the cell membrane (Figure S18G, Supporting Information) on D1 of differentiation compared to undifferentiated hESCs. The translocated GRP78 can subsequently function as a cell‐surface receptor for CRIPTO,^[^ ^29^ ^]^ a crucial regulator of mesendodermal development.^[^ ^30^ ^]^ Furthermore, consistent with the fact that apoptosis is not induced at D1, the expression of another known ATF4 target CHOP, which initiates apoptosis in cells experiencing irreversible ER stress,^[^ ^7b^ ^]^ was not increased in PERK KO on D1 (Figure S18E,F, Supporting Information). These data collectively suggest that ATF4 is regulated by PERK and may act as the downstream effector of PERK to specify mesendoderm from hESCs.

To test this hypothesis, we transiently overexpressed ATF4 for 24 hours to restore its expression on PERK KO clones by using the doxycycline‐inducible lentivirus (Figure 5B,C). Remarkably, enforced expression of ATF4 rescued important aspects of the PERK null phenotype, including restoring the majority of the cardiogenic potential (Figure 5D,E; Figure S19A–D, Supporting Information) and significantly alleviating the accumulation of protein aggregates at D1 (Figure 5F,G; Figure S19E,F, Supporting Information) induced by PERK KO. To determine whether ATF4 is similarly necessary for maintaining protein homeostasis during mesendoderm specification and subsequent cardiomyogenesis, as PERK is, we depleted ATF4 using two specific small hairpin RNAs (shRNAs) in H1 hESCs (Figure 5H,I). Consistent with the observations on PERK KO hESCs, analysis of the pluripotent and proliferative markers in control (shScram) and two *ATF4* KD cell lines (shATF4‐1 and shATF4‐2) suggested that *ATF4* KD does not affect hESC self‐renewal (Figure S20, Supporting Information). However, *ATF4* KD closely resembled the cardiac differentiation phenotypes upon PERK depletion, resulted in remarkable reduction in CM formation at D10 (Figure 5J,K; Figure S21A–C, Supporting Information) and increased accumulation of protein aggregates at D1 (Figure 5L,M). These phenotypes were confirmed independently by *ATF4* KD in WTC hiPSCs (Figure S21D–J, Supporting Information). Consistently, the total protein ubiquitin level was much higher in *ATF4* KD mesendoderm (Figure S21K,L, Supporting Information) and mRNA expression levels of key regulatory genes for mesoderm (D2), CPCs (D5), and CMs (D10) were significantly attenuated after *ATF4* KD (Figure 5N). Taken together, these results support the hypothesis that ATF4 is the critical mediator of the PERK‐dependent molecular network that guides cellular proteostasis and CM differentiation of hPSCs.

### 2.6 ATF4 Binds to and Regulates the Transcription of UPR Genes in Mesendoderm Cells

**Figure 6:**
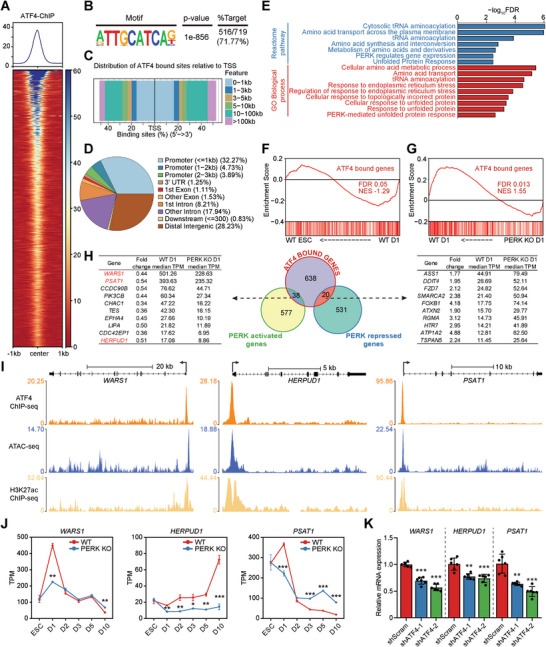
Genome‐wide occupancy of ATF4 in mesendoderm cells. A) ChIP‐seq signal heatmap of ATF4 in mesendoderm cells at D1 using a ±1 kb window centered on peak regions. ChIP‐seq signal was sorted in descending order by signal intensity. B) Motif enriched at ATF4 bound sites in mesendoderm cells at D1. C) Distribution of the distance of ATF4 bound sites relative to the transcriptional start sites in mesendoderm cells at D1. D) Fractions of genomic annotations of ATF4 bound sites in mesendoderm cells at D1. E) GO and Reactome Pathways enrichment analysis of the nearest neighboring genes of ATF4 bound sites. F,G) GSEA of the RNA‐seq data from WT ESCs and WT D1 mesendoderm cells F), as well as WT and PERK KO mesendoderm cells at D1 G). Set of the nearest neighboring genes of ATF4 bound sites, terms as “ATF4 bound genes” is used. H) Venn diagram (middle panel) outlining the overlap between genes positively and negatively regulated by PERK and ATF4 bound sites in mesendoderm cells at D1. Representative overlapping genes and their expression levels are represented in the left and right panel. I) Genome browser view showing the tracks of ATF4 ChIP‐seq, H3K27ac ChIP‐seq, and ATAC‐seq signals at the *WARS1*, *HERPUD1*, and *PSAT1* gene loci, as indicated. J) Expression levels of *WARS1*, *HERPUD1*, and *PSAT1* during CM differentiation of WT and PERK KO hESCs revealed by RNA‐seq. *n* = 3 biologically independent experiments. ^*^ *P*<0.05, ^**^ *P*<0.01, ^***^ *P*<0.001 versus WT. K) RT‐qPCR analysis of *WARS1, HERPUD1*, and *PSAT1* in shScram control and *ATF4* KD mesendoderm cells at D1. *n* = 6 biologically independent experiments. ^**^ *P*<0.01, ^***^ *P*<0.001 versus shScram. Data represent mean ± SD. Statistical significance was determined by unpaired, two‐tailed *t*‐tests J) and one‐way ANOVA with a post‐hoc Tukey test K).

To understand how ATF4 regulates mesendoderm development and to further identify genes directly regulated by ATF4 in mesendoderm, we analyzed chromatin occupancy of ATF4 at D1 of CM differentiation by chromatin immunoprecipitation with sequencing (ChIP‐seq). ChIP‐seq detected 719 ATF4 binding sites associated with 696 genes across the genome (**Figure** **6**A). The ATF4 binding motif was overrepresented in the sequences bound by ATF4 as previously reported^[^ ^31^ ^]^ (Figure 6B), supporting the fidelity of the ChIP‐seq data set. ATF4 binding was mostly found in promoters (≤3 kb to gene bodies, 40%), introns (26%), and distal intergenic regions (28%), as expected for transcription factors (Figure 6C,D). Enrichment analysis of ATF4‐bound genes identified Reactome Pathway and GO Biological Process terms that are closely associated with protein homeostasis, translation, and UPR (Figure 6E). Supporting the thought that ATF4 may globally intersect with the transcription network for mesendoderm commitment under the control of PERK, we observed increased expression of ATF4‐bound genes at D1 compared to that of undifferentiated ESCs (Figure 6F), whereas PERK KO adversely reduced the expression of these genes (Figure 6G).

Intersecting regions bound by ATF4 with PERK‐regulated genes at D1 (615 activated and 551 repressed genes) identified 58 potential direct transcriptional targets of ATF4 (38 activated and 20 repressed genes) (Figure 6H). Notably, the ATF4‐repressed targets contain many genes important for hESC pluripotency maintenance (e.g., *KLF4* and *FZD7* ^[^ ^32^ ^]^ and neural development (e.g., *FOXB1*,^[^ ^33^ ^]^ *ATP1A2*,^[^ ^34^ ^]^ *DDIT4*,^[^ ^35^ ^]^ *SMARCA2*,^[^ ^36^ ^]^ and *RGMA*,^[^ ^37^ ^]^ suggesting that ATF4 may transcriptionally repress pluripotency and neural genes to ensure proper lineage segregation during mesendoderm specification. As expected, the ATF4‐activated targets involve many known UPR genes that regulate protein homeostasis (e.g., *HERPUD1*, *PSAT1*, and *CHAC1*). Moreover, we identified *WARS1*, which encodes the tryptophanyl‐tRNA synthetase 1, as an ATF4‐activated gene with the highest level of expression at D1 among other targets (Figure 6H). WARS1 catalyzes the aminoacylation of tRNA with tryptophan, enabling the linkage of the amino acid with nucleotide triplets contained in tRNAs, an essential first step in protein synthesis.^[^ ^38^ ^]^ We thus speculated that *WARS1*, together with other ATF4‐activated UPR genes, may govern mediate the roles of PERK‐ATF4 in cardiac differentiation. By intersection of our ChIP‐seq results with published datasets of transposase‐accessible chromatin and sequencing (ATAC‐seq) in mesoderm^[^ ^39^ ^]^ and H3K27ac ChIP‐seq in ESCs from the encode database,^[^ ^40^ ^]^ we further confirmed the robust ATF4 occupation, as well as gain of chromatin accessibility and H3K27ac deposition at the transcription start sites of *WARS1*, *HERPUD1*, and *PSAT1*, three highly expressed genes at D1 (Figure 6I), reinforcing the notion that these genes are direct targets activated by ATF4. In sum, ATF4 may transcriptionally repress pluripotency/neural genes and activate genes that protect against ER stress that could occur during ESC‐to‐mesendoderm fate transition by directly binding to these gene loci.

### 2.7 WARS1 is a Key Transcriptional Target of ATF4 and Mediates PERK‐ATF4 Activity during CM Differentiation

**Figure 7:**
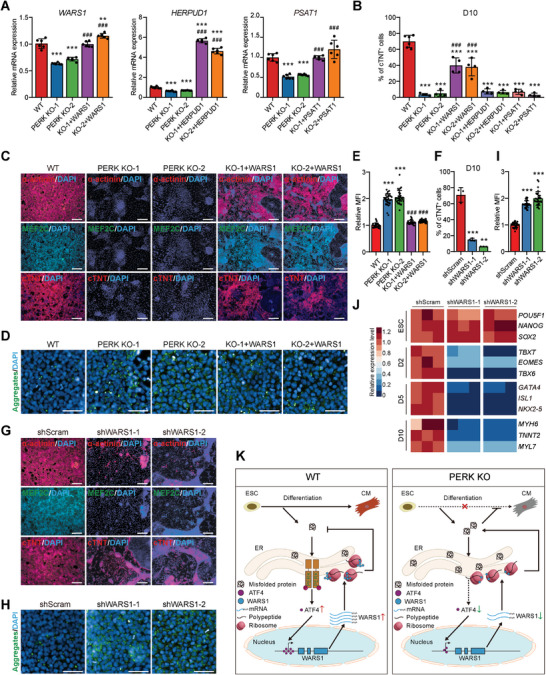
*WARS1* is a key downstream transcriptional mediator of PERK‐ATF4 during mesendoderm differentiation. A) RT‐qPCR analysis of *WARS1, HERPUD1*, and *PSAT1* in D1 mesendoderm cells differentiated from WT, PERK KO, and WARS1‐overexpressed PERK KO hESCs. *n* = 6 biologically independent experiments. ^**^ *P*<0.01, ^***^ *P*<0.001 versus WT; ^###^ *P*<0.001 versus the corresponding PERK KO clone. B) Quantitative flow cytometric analysis of cTNT^+^ cells in D10 cultures differentiated from WT and PERK KO hESCs, as well as PERK KO hESCs that receive WARS1‐, HERTUD1‐, or PSAT1‐overexpresstion, respectively. *n* = 6 (WT) or 4 (other groups) biologically independent experiments. ^***^ *P*<0.001 versus WT; ^###^ *P*<0.001 versus the corresponding PERK KO clone. C) Immunofluorescence analysis of CM markers in D10 cultures differentiated from WT, PERK KO, and WARS1‐overexpressed PERK KO hESCs. Scale bars, 200 µm. D,E) Representative D) and quantitative E) immunofluorescence analysis of the protein aggregates in D1 mesendoderm cells differentiated from WT, PERK KO, and WARS1‐overexpressed PERK KO hESCs. *n* = 3 biologically independent experiments, ten fields of view per experiment. Scale bars, 50 µm. ^***^ *P*<0.001 versus WT; ^###^ *P*<0.001 versus PERK KO. F) Quantitative flow cytometric analysis of cTNT^+^ cells in D10 cultures differentiated from shScram control and *WARS1* KD hESCs. shWARS1‐1 and shWARS1‐2 represent two independent *WARS1* shRNAs. *n* = 3 biologically independent experiments. ^**^ *P*<0.01, ^***^ *P*<0.001 versus shScram. G) Immunofluorescence analysis of CM markers in D10 cultures differentiated from shScram control and *WARS1* KD hESCs. Scale bars, 200 µm. H,I) Representative H) and quantitative I) immunofluorescence analysis of the protein aggregates in D1 mesendoderm cells differentiated from shScram control and *WARS1* KD hESCs. *n* = 3 biologically independent experiments, ten fields of view per experiment. Scale bars, 50 µm. ^***^ *P*<0.001 versus shScram. J) Heatmap showing the relative expression level of marker genes of each differentiation stage in shScram control and *WARS1* KD cells, determined by RT‐qPCR. *n* = 3 biologically independent experiments. K) Schematic model of PERK‐mediated protein hemostasis control and cell fate regulation during cardiogenesis of hESCs. Data represent mean ± SD. Statistical significance was determined by one‐way ANOVA with a post‐hoc Tukey test.

We next determined whether *WARS1*, *HERPUD1*, and *PSAT1* are directly associated with PERK‐ATF4‐mediated protein homeostasis maintenance during mesendoderm specification and subsequent CM differentiation. As profiled by RNA‐seq and quantitative PCR (qPCR) analysis, expression level of all three genes elevated in WT cells at D1 of differentiation compared to ESCs and significantly decreased after PERK deletion (Figure 6J) or *ATF4* KD (Figure 6K). To measure their impacts on cell fate outcomes, we individually overexpressed them for 24 h to transiently restore their expression on both PERK KO hESC clones by using the doxycycline‐inducible lentivirus (**Figure** **7**A). Only overexpression of WARS1 conferred visible amendment against PERK KO‐induced cardiomyogenic defect (Figure 7B,C; Figure S22A, Supporting Information). We further confirmed the phenotype by overexpression of WARS1 in PERK KO WTC hiPSCs (Figure S22B–D, Supporting Information). These data indicate WARS1 as a major target that underlines PERK‐ATF4‐governed mesendoderm specification and subsequent CM differentiation. This notion was further supported by the fact that WARS1 overexpression significantly decreased the PERK KO‐induced unfolded protein aggregate accumulation at D1 (Figure 7D,E; Figure S22E,F, Supporting Information).

Furthermore, to determine whether WARS1 can phenocopy PERK and ATF4 in protecting mesendoderm progenitors from differentiation‐induced ER stress and safeguards hPSC cardiomyogenesis, we depleted WARS1 in H1 hESCs using two specific shRNAs (Figure S23A, Supporting Information). Once again, the shScram and two *WARS1* KD cell lines (shWARS1‐1 and shWARS1‐2) had no differences in expressing the pluripotent and proliferative markers (Figure S23B–D, Supporting Information), indicating that *WARS1* KD does not affect hPSC self‐renewal. However, *WARS1* KD exactly mimicked the phenotypes of PERK or ATF4 depletion, resulted in similar degrees of decline in CM formation at D10 (Figure 7F,G; Figure S24A–C, Supporting Information) and accumulation of unfolded protein aggregates at D1 (Figure 7H,I). These phenotypes were further independently confirmed by *WARS1* KD in WTC hiPSCs (Figure S24D–I, Supporting Information). Similarly, the total protein ubiquitin level was much higher in *WARS1* KD mesendoderm (Figure S24J,K, Supporting Information) and qPCR analysis detected obvious decreases in mRNA expression levels of key regulatory genes for mesoderm (D2), CPCs (D5), and CMs (D10) after *WARS1* KD (Figure 7J). In aggregate, these results indicate that PERK‐ATF4‐WARS1 signaling resolves ER stress during mesendoderm specification and is critical for cells to adopt a new fate during cardiac differentiation of hPSCs (Figure 7K).

## 3 Discussion

Lineage commitment is modeled as a network of regulatory programs that direct dynamic gene expression and ultimately mediate the precise control of a cell's proteome, in the process of determining the cellular identity. Dynamic regulation of these programs is critical for cell fate transitions during heart development, and dysregulation can lead to developmental failure and congenital heart disease. Though many of the genetic and epigenetic factors that govern heart development are known,^[^ ^41^ ^]^ whether protein homeostasis control represents an additional layer of regulation remains unexplored. Our study proves that proteostasis maintained by the PERK‐ATF4‐WARS1 signaling axis is critical for pluripotency exit, enabling hESCs to enter the mesendoderm and subsequent cardiogenic program (Figure 7K). hESCs deficient in PERK fail mesendoderm specification and retain pluripotency, eventually undergo neural differentiation despite the presence of the mesendoderm‐inducing and neural blocking signals.

Our results establish a previously unrecognized link between UPR signaling and human cardiac lineage commitment. Most of the examples of UPR's physiological roles are related to its function in highly secretory cells (e.g., pancreatic β cells) which constantly cope with the burden of protein synthesis overload and efficiently transport and secrete newly synthesized proteins.^[^ ^42^ ^]^ CMs are the fundamental units of the heart. They produce a large number of membrane‐located ion channels and secreted proteins (e.g., extracellular matrix proteins, hemodynamic hormone, and atrial natriuretic peptide) to maintain proper contractility and endocrine/paracrine function of the heart.^[^ ^43^ ^]^ Thus, how CMs within the adult heart oversee and govern the integrity of secreted and membrane protein synthesis in both physiological and pathological conditions has attracted the attention of more and more scholars recently.^[^ ^44^ ^]^ However, the protein quality control mechanisms during embryonic heart development remain less investigated, especially in humans.

It has been shown that exposure of mouse embryos to short‐term gestational hypoxia induces heart defect at the outflow tract.^[^ ^45^ ^]^ This is accompanied by the rapid induction of UPR and perturbed cardiogenic signals in cardiac mesoderm and CPCs, suggesting that UPR may be involved in embryonic heart development. By using endothelial‐specific PERK conditional knockout mouse model, it has been shown that PERK signaling is required for embryonic cardiac valve development.^[^ ^46^ ^]^ A recent work has also reported that pharmacological activation of ATF6 in hESCs promotes their mesodermal differentiation.^[^ ^47^ ^]^ Moreover, the UPR is activated and regulates cardiac remodeling during pressure‐overload induced heart failure,^[^ ^48^ ^]^ a process known to recapitulate many key events of embryonic heart development.^[^ ^2b^ ^]^ For example, PERK knockout mice exhibited increased CM apoptosis and decreased cardiac function after thoracic aortic constriction, suggesting a protective role of PERK under pressure overload.^[^ ^48a^ ^]^ These studies collectively suggest a role of UPR in regulating heart development. Here, we provide direct evidence of PERK in fine tuning human cardiac lineage commitment, especially at the early stage. In addition, considering PERK deficiency results in failure of mesendoderm specification, PERK may not only affect cardiac development but also have broader effects on other mesendoderm‐derived cells (e.g., endoderm cells shown in Figure 4). Therefore, the findings presented here may represent a conserved mechanism for protein hemostasis maintain and cell fate commitment in mesendoderm and their progenies. Interestingly, the elevation of the IRE1α‐associated genes is visible along with the emergence of CMs during hESC cardiac differentiation (Figure 1J,K ), suggesting that the IRE1α brunch of UPR may regulate CM proteostasis to preserve its function and warrant further investigation.

Our work directly links UPR to early cell fate determination of hPSCs. Recent studies have demonstrated that the UPR plays essential roles in regulating the survival, self‐renewal, or proliferation of many tissue stem cell (TSC) types that are long‐lived, such as hematopoietic stem cells,^[^ ^49^ ^]^ skeletal muscle satellite cells,^[^ ^50^ ^]^ and intestinal stem cells.^[^ ^51^ ^]^ In this scenario, TSCs can interrogate ER stress and use differential UPR activation to either promote their self‐renewal/proliferation through relieving mild proteostatic stress, or to eliminate individual stem cell that is subjected to severe stress and damage via inducing the cell death program, thereby ensuring long‐term tissue homeostasis. In contrast to the TSCs above‐mentioned, PERK does not affect the self‐renewal or proliferation of undifferentiated hESCs despite highly expressed (Figure S4C‐G, Supporting Information). However, its absence results in the accumulation of unfolded proteins, leading to severe defects in pluripotency exit and altered cell fate decisions. These results lead to the proposition of a new paradigm in hPSCs, in which early activation of the UPR in an undifferentiated state is not directly prerequisite for cell self‐renewal/proliferation, but instead, can actually prime the cell for the future demands of plenty new proteins after differentiation, thereby ensuring proteostasis during rapid lineage transitions.

Although it is known that ER stress can trigger an integrated UPR program that is coordinated by three distinct branches (IRE1α, ATF6, and PERK), a central question is why metazoan cells have evolved three independent and mechanistically distinct sensors of protein misfolding. Accumulated evidence suggests both distinct and overlapping functions of the three UPR pathways and the same perturbation can lead to bifurcated UPR branch activation. For example, by using the Perturb‐seq, a multiplexed single‐cell CRISPR screening platform, Adamson et al. dissected the UPR program and found that each UPR branch could operate independently and being executed in markedly different ways within a homogeneous population.^[^ ^52^ ^]^ In addition, differential activation of UPR branches is observed in some stem cell populations. For example, PERK signaling is predominately activated in hematopoietic stem cells compared to downstream progenitor populations, whereas the IRE1α brunch has an opposite activity and is majorly activated in progenitors.^[^ ^49^ ^]^ Consistently, myeloid cell and eosinophil progenitors selectively activate the IRE1α signals without inducing parallel UPR pathways.^[^ ^53^ ^]^ Furthermore, ER stress activates PERK but not IRE1α brunch in adipose‐derived stem cells and attenuates their adipocyte differentiation.^[^ ^54^ ^]^ Therefore, we believe that hPSCs can similarly interrogate ER stress and use differential UPR activation (refers specifically to PERK here) to mitigate against protein misfolding and safeguard mesendoderm lineage commitment.

By performing ATF4 ChIP‐seq, we identified downstream genes directly bound and regulated by the PERK‐ATF4 signaling during CM differentiation. We found that efficient cardiac specification requires the tryptophanyl‐tRNA synthetase WARS1, a conserved transcriptional target of ATF4 also identified in mouse fibroblasts^[^ ^55^ ^]^ and human glioblastoma cells.^[^ ^56^ ^]^ Aminoacyl‐tRNA synthetases (ARSs) are essential enzymes for protein synthesis^[^ ^57^ ^]^ and have not been previously implicated in ESC biology and heart development. ARSs ligate amino acids to their corresponding tRNAs to initiate protein synthesis in a high‐fidelity manner, with one for each amino acid. The fidelity is of high functional importance for precise protein synthesis and folding, as mice harboring a missense mutation in alanyl‐tRNA synthetase which disrupts the specific interaction between the ARS and its cognate tRNA showed accumulation of misfolded proteins, leading to neurodegeneration.^[^ ^58^ ^]^ Although the conserved catalytic mechanism of ARSs for building proteins is well understood, their functions have expanded throughout evolution have only recently come to light.^[^ ^59^ ^]^ Accumulated evidences suggest that each ARS has a unique role in building and controlling complex systems^[^ ^59b^ ^]^ or involves in various human diseases,^[^ ^59a^ ^]^ in a manner that coordinates with their catalytic activity. However, the understanding of the physiological roles and underlined mechanisms of ARSs in human cardiogenesis is extremely poor. In the present study, we prove that WARS1, a direct target of ATF4, is essential for PERK‐mediated proteostasis maintain and cardiac lineage commitment (Figure 7F–J). This is consistent with the fact that loss‐of‐function mutation in *WARS1* is associated with developmental defects of the musculoskeletal system development, which consists of many mesodermal derivatives such as the bones, muscles, and cartilage, according to the Human Phenotype Ontology database.^[^ ^60^ ^]^ The aforementioned connection between ATF4 and WARS1 strongly points to a model in which aminoacylation of tRNA is directly linked to UPR to ensure correct protein folding and cellular homeostasis in human cardiogenesis (Figure 7K).

In sum, our data indicate that proteostasis safeguarded by UPR is also a core component of the cardiogenic regulatory network, thus providing a framework for understanding how molecular control of protein hemostasis is coordinated within cell fate determination. A further step will be to examine the interconnectedness between proteostasis and the other regulators of cardiogenesis, for example, in‐depth analysis of how epigenetic and transcriptional regulators rewire the UPR network to cope with each differentiation state. It will also be interesting to learn whether other protein quality control machinery, such as the autophagy system, is involved and orchestrated with the UPR to regulate cell fate transition.

## 4 Experimental Section

### Cell Culture

H1 human embryonic stem cells (hESCs) (obtained from the WiCell Research Institute) and WTC human induced pluripotent stem cells (hiPSCs)^[^ ^10^ ^]^ were grown on Matrigel (BD, 354 277)‐coated 6‐well plates in E8 medium (STEMCELL Technologies, 05 940) at 37 °C with 5% CO_2_. Cells were passaged every 3–4 days using 0.5 mm EDTA (Thermo Fisher, AM9260G) in Dulbecco's phosphate buffered saline (DPBS) without Ca^2+^ or Mg^2+^ (Gibco, 14 190 136) at 37 °C. 5 µm Rho kinase inhibitor Y‐27632 (Selleck, S1049) was added for the first 24 h after passaging. The E8 medium was changed every day. HEK293T cells (ATCC, CRL‐321) were cultured in high glucose Dulbecco's Modified Eagle's Medium (DMEM, Hyclone, SH30022.01) supplemented with 10% fetal bovine serum (FBS, Hyclone, SH30406.05) and 2 mm GlutaMAX (Gibco, 35 050 061) at 37 °C with 5% CO_2_. HEK293T cells were passaged with TrypLE Express (Gibco, 12 604 021) and the culture medium was changed every other day.

### Cardiomyocyte (CM) Differentiation

Undifferentiated hESCs or hiPSCs cultured in E8 medium were dissociated into single cell suspension by Accutase (STEMCELL Techologies, 7 920) and reseeded onto Matrigel‐coated 24‐well plate at a density of 10^5^ cells per well in E8 medium containing 10 µm Y‐27632. When cells reach ≈80% confluence 2–3 days after plating, CM differentiation was initiated by switching to the differentiation medium named E8 basal+Lip (DMEM/F‐12 (Gibco, 11 330 032) supplemented with 50 U mL^−1^ Penicillin‐Streptomycin (Gibco, 15 070 063), Chemically Defined Lipid Concentrate (1:100, Gibco, 11 905 031), 10.7 µg mL^−1^ holo‐Transferrin human (Sigma‐Aldrich, T0665), 71 µg mL^−1^ L‐Ascorbic acid (Sigma‐Aldrich,, A8960), 14 ng mL^−1^ Sodium selenite (Sigma‐Aldrich, S5261)). 5 µm CHIR99021 (Selleck, S1263) or 3 µm IWP2 (Selleck, S7085) was added into the cardiac differentiation medium from days 0–1 and days 2–5, respectively. 3 µg mL^−1^ heparin was added into the cardiac differentiation medium from days 1–7. 20 µg mL^−1^ Insulin (Sigma‐Aldrich, 91077C) was added into the cardiac differentiation medium from day 7 onward and renewed every 2–3 days.

CM differentiation by the RPMI‐B27 method was described previously.^[^ ^14^, ^15^ ^]^ Briefly, undifferentiated hESCs were dissociated and plated as set forth. CM differentiation was initiated by switching to the RPMI‐B27 medium (RPMI1640 (Thermo Fisher, C11875500BT) supplemented with the B27 Minus Insulin supplement (Gibco, A1895601)). 12 µm CHIR99021 and 3 µm IWP2 were added into the CM differentiation medium from days 0–1 and days 3–5, respectively. B27 Minus Insulin supplement was replaced with the standard B27 supplement (Gibco, 12 587 010) from day 7 onward and the culture medium was renewed every 2–3 days.

Atrial‐like CMs were induced according to a previous study with minor modifications.^[^ ^16^ ^]^ Briefly, undifferentiated hESCs were reseeded onto Matrigel‐coated 12‐well plate at a density of 2.5 × 10^4^ cells per well and allowed to grow for four days. Then cells were cultured in E8 basal+Lip medium supplemented with 6 µm CHIR99021, 3 µm IWP2, and 2 µm Retinoic acid (Sigma‐Aldrich, R2625) from days 0–1, days 2–3, and days 4–5, respectively. Differentiated atrial CMs were maintained in RPMI1640 containing the B27 supplement from day 8 onward.

CMs with advanced maturity were generated by coculture of the differentiating CPCs with hESC‐derived endothelial cells (ECs)^[^ ^21b^ ^]^ with minor modification. Briefly, CMs were differentiated by the heparin method as described above. At differentiation day 6, cells were dissociated into single cell suspension by Accutase, mixed with the H1 hESC‐derived ECs at a ratio of 3:1, plated onto Matrigel‐coated tissue culture plates at a density of 1.6 × 10^5^ cells cm^−2^ in EC medium (DMEM supplemented with 10% FBS and 5 µM Y‐27632), and cultured for 2 weeks. Cells were then treated with 50 µM WY‐14643, a PPARα agonist that promotes CM maturation^[^ ^61^ ^]^ in E8 basal+Lip medium added with 20 µg mL^−1^ Insulin for 4 days, with daily change of the culture medium. Finally, CMs with advanced maturity were purified by firstly culturing in glucose‐ and sodium pyruvate‐free DMEM (Gibco, 11966‐025) supplemented with 20 mm lactate (Sigma‐Aldrich, L7022)^[^ ^62^ ^]^ and then sorted by a BD FACS ARIA SORP, Influx sorter (BD) using an APC Mouse Anti‐Human CD36 antibody (Biolegend, 336 208) as described previously.^[^ ^21a^ ^]^

### Cardioid Differentiation

Cardioid differentiation was described previously.^[^ ^17^ ^]^ Briefly, undifferentiated hESCs cultured in E8 medium were dissociated into single cell suspension by Accutase and seeded in a volume of 200 mL into ultra‐low‐attachment 96‐well plates (Corning) containing 10 µm Y‐27632 and collected by centrifugation for 5 min at 200 g. Cardioids differentiation was initiated by switching to the differentiation medium named E8 basal+Lip (DMEM/F‐12 (Gibco, 11 330 032) supplemented with 50 U mL^−1^ Penicillin‐Streptomycin (Gibco, 15 070 063), Chemically Defined Lipid Concentrate (1:100, Gibco, 11 905 031), 10.7 µg mL^−1^ holo‐Transferrin human (Sigma‐Aldrich, T0665), 71 µg mL^−1^ L‐Ascorbic acid (Sigma‐Aldrich, A8960), 14 ng mL^−1^ Sodium selenite (Sigma‐Aldrich, S5261)). 30 ng mL^−1^ FGF2, 5 µm LY294002, 50 ng mL^−1^ Activin A, 10 ng mL^−1^ BMP4, and 3 µm CHIR99021 was added into the medium from days 0–1.5. 1 mg mL^−1^ of Insulin was optionally added to increase cell viability during this stage. 10 ng mL^−1^ BMP4, 8 ng mL^−1^ FGF2, 10 ng mL^−1^ Insulin, 5 µm IWP2, and 0.5 µm Retinoic acid was added from days 1.5–5.5 with medium change every day. 10 ng mL^−1^ BMP4, 8 ng mL^−1^ FGF2, 10 ng mL^−1^ Insulin was added from days 5.5–7.5 with medium change every day.

### Lateral Mesoderm Differentiation

Undifferentiated hESCs were dissociated into very fine clumps by Accutase and sparsely passaged at a ratio of 1:12 to1:20 in E8 medium containing 10 µm Y‐27632 overnight. The next morning, differentiation was initiated by switching to the E8 basal+Lip and renewed every day. 30 ng mL^−1^ Activin A (PeproTech, 12014E), 40 ng mL^−1^ BMP4 (Gibco, PHC9531), 6 µm CHIR99021, 20 ng mL^−1^ FGF2 (Peprotech, AF‐100‐18B), and 10 µm LY294002 (Selleck, S1105) were added for the first 24 h. 1 µm A83‐01 (Selleck, S7692), 30 ng mL^−1^ BMP4, and 1 µm C59 (Selleck, S7037) were added for next 24 h.

### Definitive Endoderm Differentiation

Undifferentiated hESCs cultured in E8 medium were dissociated into single cell suspension by Accutase and reseeded onto Matrigel‐coated 24‐well plate at a density of 10^5^ cells per well in E8 medium containing 10 µm Y‐27632. When reached 80% confluency, definitive endoderm differentiation was initiated by switching to the endoderm differentiation medium (DMEM/F‐12 supplemented with 50 U mL^−1^ Penicillin‐Streptomycin, Chemically Defined Lipid Concentrate (1:100), 10.7 µg mL^−1^ holo‐Transferrin human, 71 µg mL^−1^ L‐Ascorbic acid, 14 ng mL^−1^ Sodium selenite, and 100 ng mL^−1^ Activin A) and cultured for 3 days. CHIR99021 at 3 µm was added to the medium for the first 24 h of differentiation and removed thereafter. Samples were collected and analyzed on day 3. We routinely obtain greater than 80% differentiated cells based on the presence of the nucleus markers SOX17 and FOXA2 for definitive endoderm.

### Ectoderm Differentiation

Undifferentiated hESCs cultured in E8 medium were dissociated into fine clusters by 0.5 mm EDTA and reseeded onto Matrigel‐coated 24‐well plate at a density of 5 × 10^5^ cells per well in E8 medium containing 10 µm Y‐27632. When hESCs reached ≈95% confluency 1–2 days after reseeding, ectoderm differentiation was initiated by switching to the ectoderm differentiation medium (DMEM/F‐12 supplemented with 50 U mL^−1^ Penicillin‐Streptomycin, 10.7 µg mL^−1^ holo‐Transferrin human, 71 µg ml^−1^ L‐Ascorbic acid, 14 ng mL^−1^ Sodium selenite, 19.4 ng mL^−1^ Insulin, 10 ng mL^−1^ BMP4, 10 µm SB431542 (Selleck, S1067), and 10 µm SU5402 (Selleck, S7667)) and changed every day. Two days later, the concentration of BMP4 was reduced to 5 ng mL^−1^ and the medium was renewed every two days until the cultures were analyzed at day 8. We routinely obtain greater than 90% differentiated cells based on the presence of the nucleus marker PAX6 for ectoderm.

### Cas9‐Mediated PERK Knockout hESC and hiPSC Lines

Two sgRNAs create paired sgRNA target loci for SpCas9‐nickase (D10A mutation) and were designed using a web server tool from Dr. Feng Zhang's lab (http://crispr.mit.edu/). Two sets of oligonucleotides (sgPERK‐1 and sgPERK‐2) were cloned into epiCas9n plasmid^[^ ^63^ ^]^ and confirmed by Sanger sequencing using the checking primers (PERK‐KO‐check‐F PERK‐KO‐check‐R). H1 hESCs and WTC hiPSCs were plated onto the 6‐well plates and transfected 24 h later with 2.5 µg of epiCas9n‐sgPERK‐1_sgPERK‐2 plasmid using the Lipofectamine™ Stem Transfection Reagent (Thermo Fisher, STEM00008). 24 h later, transfected cells were selected with 1 µg mL^−1^ puromycin (Selleck, S7417) for 36 h and reseeded onto Matrigel‐coated 6 cm culture dish at a very low density (5000 cells per dish) to allow clone forming from a single‐cell. Reseeded cells were grown in E8 medium supplemented with the CloneR reagent (STEMCELL Technologies, 05 888) until clones were large enough to pick. Picked clones were then genotyped by Sanger sequencing of the gRNA‐targeted sites. The sequences of the oligonucleotides and checking primer are listed in Table S1 (Supporting Information).

### shRNA Infection and Gene Knockdown (KD) Experiments

shRNAs for knockdown *ATF4*, *WARS1*, *HERPUD1*, or *PSAT1* were selected from predesigned shRNAs by Sigma‐Aldrich (http://www.sigmaaldrich.com/life‐science/functional‐genomics‐and‐rnai/sirna/mission‐predesigned‐sirna.html). The shRNA primers were subcloned into the pLKO.1‐blast (Addgene, 26 655) vector and confirmed by Sanger sequencing. KD lentivirus was made by co‐transfection of HEK293T cells with the Lentiviral pLKO.1 shRNA‐expressing vector, an envelope plasmid (pMD2.G, Addgene, 12 259), and a packaging plasmid (psPAX2, Addgene, 12 260) using Lipofectamine™ 2000 (Thermo Fisher, 11 668 019). Then virus‐containing medium was collected from the HEK293T cells. H1 hESCs or WTC hiPSCs were subjected to two rounds of viral infection (6 h per round) with the presence of 8 µg mL^−1^ polybrene. 48 h after the last infection, transduced cells were selected with 10 µg ml^−1^ blasticidin (Selleck, S7419) for three continuous passages. KD efficiencies of the target genes were evaluated by RT‐qPCR. Sequences of the shRNAs were listed in Table S1 (Supporting Information).

### Lentivirus Production and Generation of Stable Gene‐overexpression hESC Lines

The coding sequences of *PERK*, *ATF4*, *WARS1*, *HERPUD1*, and *PSAT1* were PCR amplified from the full‐length reverse transcript cDNA, cloned into the pLVX‐Tet‐One‐Puro (ClonTech, 631 847) vector, and confirmed by Sanger sequencing. Lentivirus production and cell transduction were performed as mentioned above. 48 h after the last infection, transduced cells were selected with 1 µg mL^−1^ puromycin for three continuous passages. The expression levels of target genes were evaluated by RT‐qPCR. Sequences of the PCR primers used are listed in Table S1 (Supporting Information).

### siRNA‐Mediated Gene KD

Cells at differentiation day 2 were transfected with the predesigned siRNA against *PERK* (GenePharma, A10002) mRNAs using the Lipofectamine‐RNAiMax reagent (Thermo Fisher, 13 778 030). The culture medium was changed 6 h after transfection and KD efficiency was examined. Scramble RNA was used as a negative control. Sequences of the siRNAs are listed in Table S1 (Supporting Information).

### ER Stress Element (ERSE)‐ and ATF4‐luciferase Reporter Assay

For measurement of the ERSE activity, hESCs were plated onto Matrigel‐coated 6‐well plates, cultured overnight, and then transfected with 2.5 µg of the ERSE‐luciferase reporter plasmid (Yeasen, 11547ES03) using the Lipofectamine™ Stem Transfection Reagent (Thermo Fisher, STEM00008). 24 h later, the transfected cells were selected with 100 µg mL^‐1^ Geneticin (G418 Sulfate, Selleck, S3028) for 36 h. Survived cells were then subjected to CM differentiation and collected for luciferase activity detection at the indicated differentiation time points using the Luciferase Assay kit (Promega, E1500), according to the manufacturer's instruction.

For measurement of the ATF4 activity, the pLVX ATF4‐luciferase plasmid, a gift from Prof. Guojun Shi, was used for lentivirus production and cell transduction as mentioned above. 48 h after the last infection, transduced cells were selected with 1 µg mL^−1^ puromycin for three continuous passages. Cells at the indicated differentiation time points were similarly collected for luciferase activity examination ut supra.

### Alkaline Phosphatase (AP) Staining

AP staining was performed using the Alkaline Phosphatase Detection Kit (Sigma‐Aldrich, SCR004) following the manufacturer's instruction. Stained cells were imaged with a Model GS‐800 Calibrated Imaging Densitometer (Bio‐Rad).

### RT‐qPCR Analysis

Total RNA was extracted using the NucleoZol reagent (Macherey‐Nagel, 740 404) and quantified by a NanoDrop spectrophotometer (ThermoFisher). Reverse transcription was performed using the HiScript II Q RT SuperMix (Vazyme, R223). RT‐qPCR was carried out using the ChamQ Universal SYBR qPCR Master Mix (Vazyme, Q711) and performed in a LC480 Real‐Time PCR System (Roche). Relative mRNA levels were normalized to those of *ACTB* or *GAPDH* mRNAs in each reaction and assessed using the comparative Ct method.^[^ ^64^ ^]^ Sequences of primers used for RT‐qPCR were listed in Table S1 (Supporting Information).

### Immunoblotting Analysis

For immunoblotting, cells were trypsinized, washed by DPBS, and lysed in RIPA buffer (Cell Signaling Technology, 9806) containing the Protease Inhibitor Cocktail (Roche, 4 693 132 001) and the Phosphatase Inhibitor Cocktail (Roche, 4 906 845 001). Lysate supernatant was used to measure protein concentration by using the BCA Protein Assay Kit (Beyotime, P0012). Supernatant was mixed with sample loading buffer (ThermoFisher, LC2676) and boiled for 10 min at 98 °C. Samples were then subjected to electrophoresis in a 5–12% SDS‐PAGE and transferred to the PVDF membrane (Millipore, IPVH00010). PVDF membranes were then blocked by 5% BSA and incubated with the primary antibodies against PERK (1:1000, Cell Signaling Technology, 3 192), ATF4 (1:1000, Cell Signaling Technology, 11 815), p‐PERK (1:1000, Invitrogen, PA5‐40294), eIF2α (1:1000, Cell Signaling Technology, 5324), p‐eIF2α (1:1000, Cell Signaling Technology, 3398), Ubiquitin (P37) (1:1000, Cell Signaling Technology, 58 395), CHOP (1:1000, Cell Signaling Technology, 2895), GRP78 (1:1000, Abclonal, A23453), or β‐actin (1:1000, 4A Biotech, 4ab030003) at 4 °C overnight, followed by incubation with the HRP‐conjugated anti‐rabbit (Promega, W4011) or anti‐mouse (Promega, W4021) secondary antibodies at room temperature (RT) for 1 h. For chemiluminescence detection, PVDF membranes were incubated with the chemiluminescence (ECL) kit (ThermoFisher, A38555) and examined by the GE ImageQuant Las4000mini Scanner.

### Biochemical Fractionation and SDS‐PAGE Analysis of Protein Insolubility

Biochemical fractionation of hESCs and mesendoderm cells based on solubility was performed as previously described.^[^ ^19^ ^]^ Briefly, cells were washed once with PBS, then two third of each sample was lysed with the RIPA buffer with Complete Mini Protease Inhibitor Cocktail (Roche, 11 836 153 001). RIPA supernatant (soluble fraction) was collected after a 15‐min centrifugation at 16 000 g at 4 °C. The remaining insoluble pellet was then washed with RIPA buffer and centrifuged 15 min at 16 000 g at 4 °C. This supernatant was discarded and the insoluble pellet was dissolved in PBS supplemented with 4% SDS, 50 mm N‐ethylmaleimide (Pierce, 23030B), 25 mm TCEP (ThermoFisher, 77 720), and 150 mm NaCl (insoluble fraction). For normalization, one third of each sample was directly lysed in PBS with 4% SDS, 50 mm N‐ethylmaleimide, 25 mm TCEP, and 150 mm NaCl (total fraction). Both the lysis solution and the working solution of N‐ethylmaleimide were freshly prepared before use. Lysates were stored at −20 °C. For visualization, lysates were run on 12.5% Omni‐EasyPAGE (EpiZyme, PG213). The concentrations of the samples were measured by the EZQ Protein Quantitation Kit (ThermoFisher, R33200) and equal concentrations of total fraction were loaded across samples. For each sample, four‐times the volume of total fraction was loaded for the insoluble fraction, and half the volume of the total fraction was loaded for the soluble fraction. Gels were stained with Coomassie Blue Fast Staining Solution (Beyotime, P0017) according to manufacturer's instructions, and imaged with a Tanon‐2000 Gel Image Analysis System. The intensity of each lane or a specified area was measured by using the ImageJ software (v1.51j8).

### Flow Cytometry Analysis

Cells were dissociated into single cell using Accutase for 5 min at 37 °C, washed twice with ice‐cold wash buffer (DPBS containing 2% FBS), resuspended in ice‐cold blocking buffer (DPBS containing 5% FBS). For surface protein detection, cells were stained with TRA‐1‐81‐DyLight 488 (1:100, Stemgent, 09–0069) and SSEA‐4‐DyLight 550 (1:100, Stemgent, 09–0087) antibodies for 1 h at 4 °C. For apoptosis testing, cells were stained with Annexin V‐FITC/PI Kit (Beyotime, C1062M) for 20 min at room temperature.

For intracellular antigen detection, cells were fixed and permeabilized using the Cytofix/Cytoperm Kit (BD, 554 714). For detecting CMs, cells were stained with cTNT antibody (1:500, Thermo Fisher, MA5‐12960) for 1 h at room temperature and followed by incubation with the anti‐mouse‐Alexa Fluor 647 secondary antibody (1:1000, Thermo Fisher, A31571) for 45 min at room temperature. For maturation testing, cells were stained with the cTNT antibody (1:500, Thermo Fisher, MA5‐12960) and α‐SMA antibody (1:500, BOSTER, BM0002) for 1 h at room temperature, and followed by incubation with the anti‐mouse IgG1‐Alexa Fluor 488 secondary antibody (1:1000, Thermo Fisher, A31571) and anti‐mouse IgG2a‐Alexa Fluor 647 secondary antibody (1:1000, Thermo Fisher, A31571) for 45 min at room temperature. For proliferation testing, cells were stained with the Ki67 antibody (1:500, Abcam, ab16667) for 1 h at room temperature, and followed by incubation with the anti‐rabbit‐Alexa Fluor 488 secondary antibody (1:1000, Thermo Fisher, A31571) for 45 min at room temperature. Then cells were washed three times, resuspended in wash buffer, and analyzed using the CytoFLEX S Flow Cytometer (Beckman Coulter). Cells incubated with the isotype‐matched control antibodies were served as negative controls, including DyLight 488‐conjugated mouse IgM isotype control (Thermo Fisher, MA1‐194‐D488), PE‐conjugated mouse IgG3 isotype (R&D, IC007P), and APC‐conjugated mouse IgG2a kappa isotype (Thermo Fisher, 17‐4724‐81). Data was analyzed using the FlowJo Software (FlowJo LCC).

### Immunofluorescence Analysis

For intracellular protein immunofluorescence, cells were fixed in 4% paraformaldehyde (PFA) for 15 min at RT and washed twice with DPBS. Cells were permeabilized in the permeabilization buffer (DPBS supplemented with 0.3% Triton X‐100) for 30 min at RT, washed once in DPBS, and blocked in the blocking‐permeabilization buffer (DPBS supplemented with 3% bovine serum albumin (BSA) and 0.1% Triton X‐100) for 1 h at RT. Cells were then stained with primary antibody diluted in the blocking‐permeabilization buffer overnight at 4 °C. Primary antibodies included SOX2 (1:200, Abcam, ab79351), NANOG (1:200, Cell Signaling Technology, 3 580), OCT4 (1:200, Santa Cruz, sc‐5279), Ki67 (1:100, BD, 550 609), cTNT (1:200, Thermo Fisher, MA5‐12960), α‐actinin (1:200, Sigma‐Aldrich, A773225), MEF2C (1:100, Cell Signaling Technology, D80C1), SOX17 (1:200, R&D, AF‐1924), FOXA2 (1:200, Cell Signaling Technology, 8186), PAX6 (1:100, Thermo Fisher, MA1‐109), ISL1 (1:50, DSHB), Brachyury (1:100, R&D, AF2085), Ubiquitin (P37) (1:200, Cell Signaling Technology, #58 395), ATF4 (1:200, Cell Signaling Technology, 11 815), and GRP78 (1:200, Abclonal, A23453). After washing with DPBS, cells were stained with secondary antibody diluted in the secondary antibody buffer (DPBS supplemented with 1% BSA and 0.05% Triton X‐100) at RT for 45 minutes. Secondary antibodies included Alexa Fluor 488‐conjugated donkey anti‐rabbit (1:800, Thermo Fisher, A21206), Alexa Fluor 488‐conjugated goat anti‐mouse (1:800, Thermo Fisher, A21121), Alexa Fluor 594‐conjugated donkey anti‐goat (1:800, Thermo Fisher, A11058), Alexa Fluor 594‐conjugated goat anti‐mouse (1:800, Thermo Fisher, A21145), Alexa Fluor 647‐conjugated chicken anti‐mouse (1:800, Thermo Fisher, A21463), Alexa Fluor 647‐conjugated donkey anti‐mouse (1:800, Thermo Fisher, A31570), and Alexa Fluor 555‐conjugated goat anti‐mouse (1:800, Thermo Fisher, A21127). Stained samples were imaged with a Leica DMi8 inverted fluorescence microscope.

For immunofluorescence analyses of misfolded and/or aggregated proteins, cells grown on the CellCarrier‐96 microplate (PerkinElmer, 6 005 550) were stained with the Proteostat Aggresome Detection Kit (Enzo, ENZ‐51035) according to the manufacturer's instructions. For analyses of apoptosis or necrosis cells, cells were stained with the TUNEL BrightRed Apoptosis Detection Kit (Vazayme, A113‐03) or Propidium Iodide (PI) (Sigma, P4170) according to the manufacturer's instructions. Stained samples were imaged using the Operetta CLS™ high‐content analysis system (Perkin Elmer). Data analyses were performed by using the Harmony 4.5 software. Thirty randomly selected fields of view were captured and analyzed per sample, using the same parameters across all samples and images.

### Teratoma Analysis

hESCs were dissociated with 0.5 mm EDTA, centrifuged at 200 g for 3 min, and resuspended in 0.5 mL DMEM/F‐12 1:1 mixed with the Matrigel. 2 × 10^6^ cells per group were injected subcutaneously into the groin of the 8‐week‐old female NOD‐SCID mice (GemPharmatech). Eight weeks after injection, teratoma was dissected and fixed with 4% PFA, and analyzed by hematoxylin‐eosin staining. Slides were imaged with an Upright metallurgical microscope (Olympus BX51). Animal experiments were approved by the institutional ethics and animal welfare committee of Sun Yat‐sen University. The size of the tumors generated in this study was within the limits allowed in the ethical guidelines of the institution.

### RNA‐Seq Assay and Data Analysis

Total RNA was extracted using the NucleoZol reagent and quantified by NanoDrop spectrophotometer. mRNA libraries were prepared using the VAHTS® mRNA‐seq V2 Library Prep Kit for Illumina from Vazyme and sequenced on an Illumina HiSeq 2000 with 150 bp paired‐end reads by GENEWIZ. Raw RNA‐seq sequenced reads were quality tested using FASTQC and were aligned to GRCh38/hg38 Homo sapiens reference genome using STAR aligner with ENCODE standard options for long RNA‐seq pipeline.^[^ ^65^ ^]^ RSEM was used to estimate gene expression abundance and calculate TPM values.^[^ ^66^ ^]^ Differential gene expression was carried out with DESeq2.^[^ ^67^ ^]^ Significant differently expressed genes were defined as having an adjusted *P*‐value < 0.05, a log2 fold change>1. Gene ontology (GO) enrichment analysis of differentially expressed genes was performed using clusterProfiler.^[^ ^68^ ^]^ CellNet analysis was performed using the CellNet R package with default settings.^[^ ^22^, ^69^ ^]^

### ChIP‐Seq Assay and Data Analysis

ChIP assays were performed following the previously published protocol.^[^ ^70^ ^]^ ChIP‐grade antibodies against ATF4 were purchased from Cell Signaling Technology (11 815). DNA without antibody immunoprecipitation was served as the input controls. ChIP‐seq libraries were generated and sequenced an Illumina HiSeq X Ten with 150 bp paired‐end reads by Novogene. Raw ChIP‐seq sequenced reads were trimmed for adapters and low sequencing quality bases using fastp.^[^ ^71^ ^]^ Data were analyzed using the Encode transcription factor ChIP‐seq standard pipeline. In brief, BWA was used to map the 150 bp paired‐end reads to the GRCh38/hg38 Homo sapiens reference genome.^[^ ^72^ ^]^ Duplicates were then marked and removed by Picard. The SPP program was used to call peaks, and an IDR‐based strategy was used to identify the stable peaks across pseudo‐replicates. Homer was used to identify enriched motifs within the called peaks.^[^ ^73^ ^]^ Peaks were annotated using ChIPseeker,^[^ ^74^ ^]^ and closest genes were used for GO or Reactome pathway enrichment in Metascape.^[^ ^75^ ^]^

### Spontaneous Ca^2+^ Transient Measurement in CMs

hESC‐derived CMs were treated with 1 µm Fluo‐4 AM (Thermo Fisher, F14201) in the Tyrode's solution (140 mm NaCl, 5 mm KCl, 2 mm MgCl_2_, 10 mm HEPES, 1.8 mm CaCl_2_, 10 mm glucose, pH 7.4) at 37 °C for 10–15 min. Fluo‐4 AM was then washed off for three times with the Tyrode's solution and Ca^2+^ transients were captured in the line‐scan model using a Zeiss LSM 710 confocal microscope with a 63× objective. During recording, cells were maintained at 37 °C in a heated chamber. The Ca^2+^ transient data were analyzed with the IDL software (ITT Corporation).

### Statistical Analysis

Values were presented as mean ± SD and quantified from at least three biological repeats unless otherwise stated. Unpaired two‐tailed student's *t*‐test was used for statistical significance between two groups if data were in a normal distribution, otherwise, the Wilcoxon test was used. For comparisons of multiple groups, one‐way analysis of variance (ANOVA) with a post‐hoc Tukey test was used. *P*‐value < 0.05 was considered two‐sided significant.

## Conflict of Interest

The authors declare no conflict of interest.

## Author Contributions

F.L., Z.L., W.C., and Q.Z. contributed equally to this work. N.C., F.L., and Z.C. conceived the project. F.L., Z.L., W.C., and Q.Z. designed, performed, and analyzed the experiments. W.C. performed the bioinformatics analysis. X.Z., H.Z., M.Y., Y.G., and Q.J. participated in molecular biology experiments. H.X. participated in animal experiments. L.W., G.S., S.G., and J.W. provided materials or assisted with the experiments. N.C., F.L, Z.L., W.C., and Z.C. wrote the manuscript.

## Supporting information

Supporting Information

## Data Availability

The data that support the findings of this study are available from the corresponding author upon reasonable request.
