# Supplementary material for: The PERK Branch of the Unfolded Protein Response Safeguards Protein Homeostasis and Mesendoderm Specification of Human Pluripotent Stem Cells
Source: Adv Sci (Weinh). 2023 Oct 27;10(35):2303799. doi: 10.1002/advs.202303799 (PMC10724406; doi:10.1002/advs.202303799)
Supplement: Supplementary file 1 — Supporting Information [file ADVS-10-2303799-s001.pdf]

ADVANCED SCIENCE
Open Access

Supporting Information

for *Adv. Sci.*, DOI 10.1002/advs.202303799

The PERK Branch of the Unfolded Protein Response Safeguards Protein Homeostasis and Mesendoderm Specification of Human Pluripotent Stem Cells

Fang Liu, Zhun Liu, Weisheng Cheng, Qingquan Zhao, Xinyu Zhang, He Zhang, Miao Yu, He Xu, Yichen Gao, Qianrui Jiang, Guojun Shi, Likun Wang, Shanshan Gu, Jia Wang, Nan Cao and Zhongyan Chen**

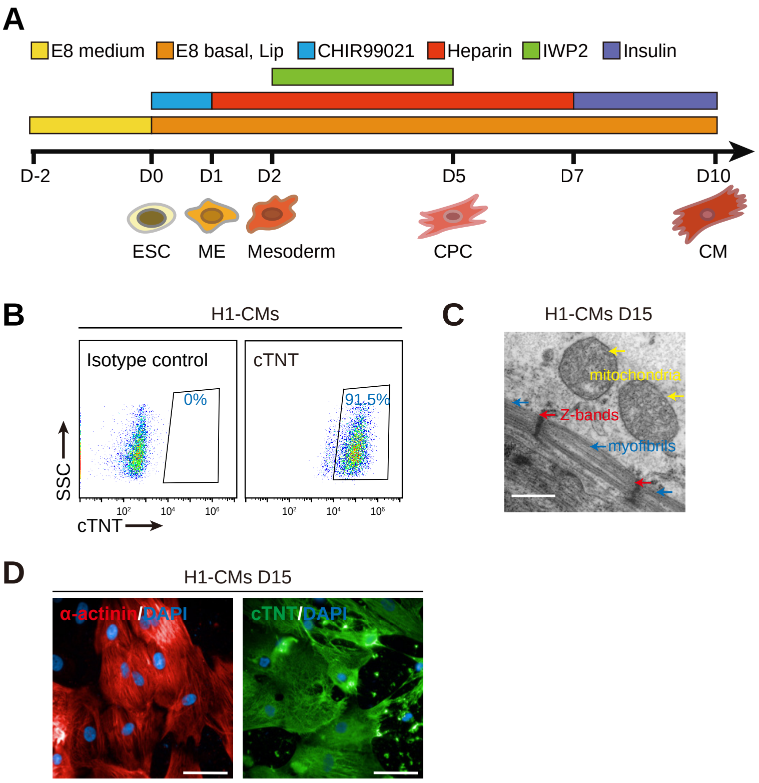

Figure S1. Differentiation of human embryonic stem cells (hESCs) into cardiomyocytes (CMs)
A, Schematic of the CM differentiation protocol of hESCs. Lip, chemically defined lipid concentrates. D, differentiation day. ESC, embryonic stem cells; ME, mesendoderm; CPC, cardiac progenitor cell.
B, Representative flow cytometry analysis of $cTNT^+$ cells in D10 cultures differentiated from H1 hESCs.
C, Transmission electron microscopic image of hESC-derived CMs showing myofibrils (blue arrows) with Z-bands (red arrows) and mitochondria (yellow arrows). Scale bar, 500 nm.
D, Immunofluorescence analysis of CM markers α-actinin and cTNT in D15 cultures differentiated from H1 hESCs. Scale bars, 50 μm.

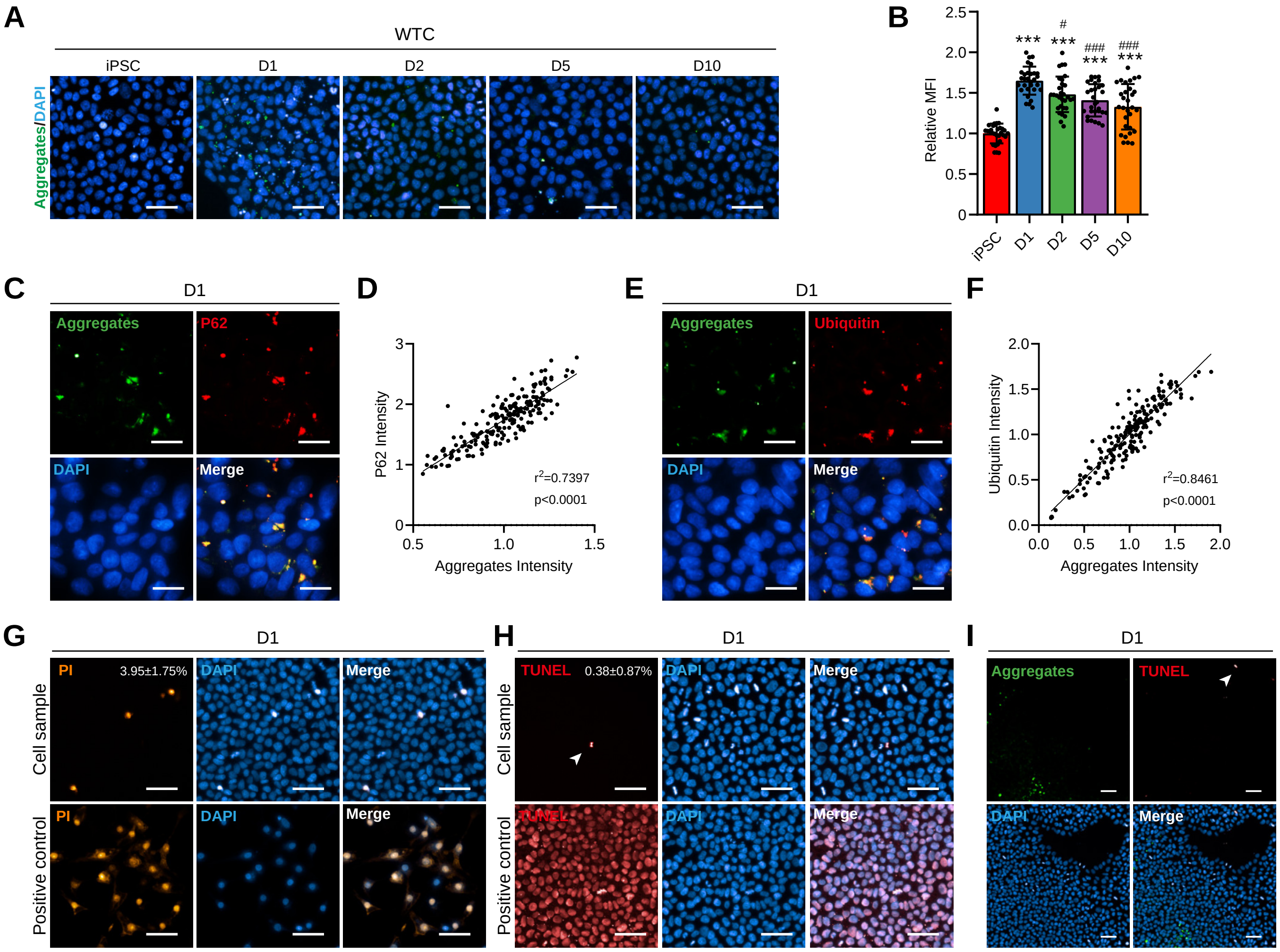

Figure S2. Identification of unfolded protein aggregates at the early stage of CM differentiation

A, Representative immunofluorescence analysis of cells at each stage of CM differentiation of WTC human induced pluripotent stem cells (hiPSCs) stained with Proteostat (green, protein aggregates). Scale bars, 50 μm.

B, Quantification of the mean fluorescence intensity (MFI) of the protein aggregates normalized to cell number in samples in (A). n = 3 biologically independent experiments, 10 fields of view per experiment. $^{***}P<0.001$ vs. iPSC; $^{\#}P<0.05$, $^{\#\#\#}P < 0.001$ vs. D1.

C, Representative immunofluorescence staining of the protein aggregates and P62 in H1 hESC-derived D1 cultures. Scale bars, 50 μm.

D, Scatter plot showing the correlation between the protein aggregates and P62 in H1 hESC-derived D1 cultures. n = 4 biologically independent experiments, 50 fields of view per experiment.

E, Representative immunofluorescence staining of the protein aggregates and ubiquitin in H1 hESC-derived D1 cultures. Scale bars, 50 μm.

F, Scatter plot showing the correlation between the protein aggregates and ubiquitin in H1 hESC-derived D1 cultures. n = 4 biologically independent experiments, 50 fields of view per experiment.

G, Representative propidium iodide (PI) staining of H1 hESC-derived D1 cultures. Fixed cells were used as positive control. n = 4 biologically independent experiments, 5 fields of view per experiment. Scale bars, 50 μm.

H, Representative TUNEL staining of H1 hESC-derived D1 cultures. Positive controls were cells treated with DNase **I**. n = 3 biologically independent experiments, 5 fields of view per experiment. Scale bars, 50 μm.

I, Representative immunofluorescence staining of the protein aggregates and TUNEL in H1 hESC-derived D1 cultures. Scale bars, 50 μm.

Data represent mean ± SD. Statistical significance was determined by one-way ANOVA with a post-hoc Tukey test (**B**) and two-tailed Pearson's correlation test (**D** and **F**).

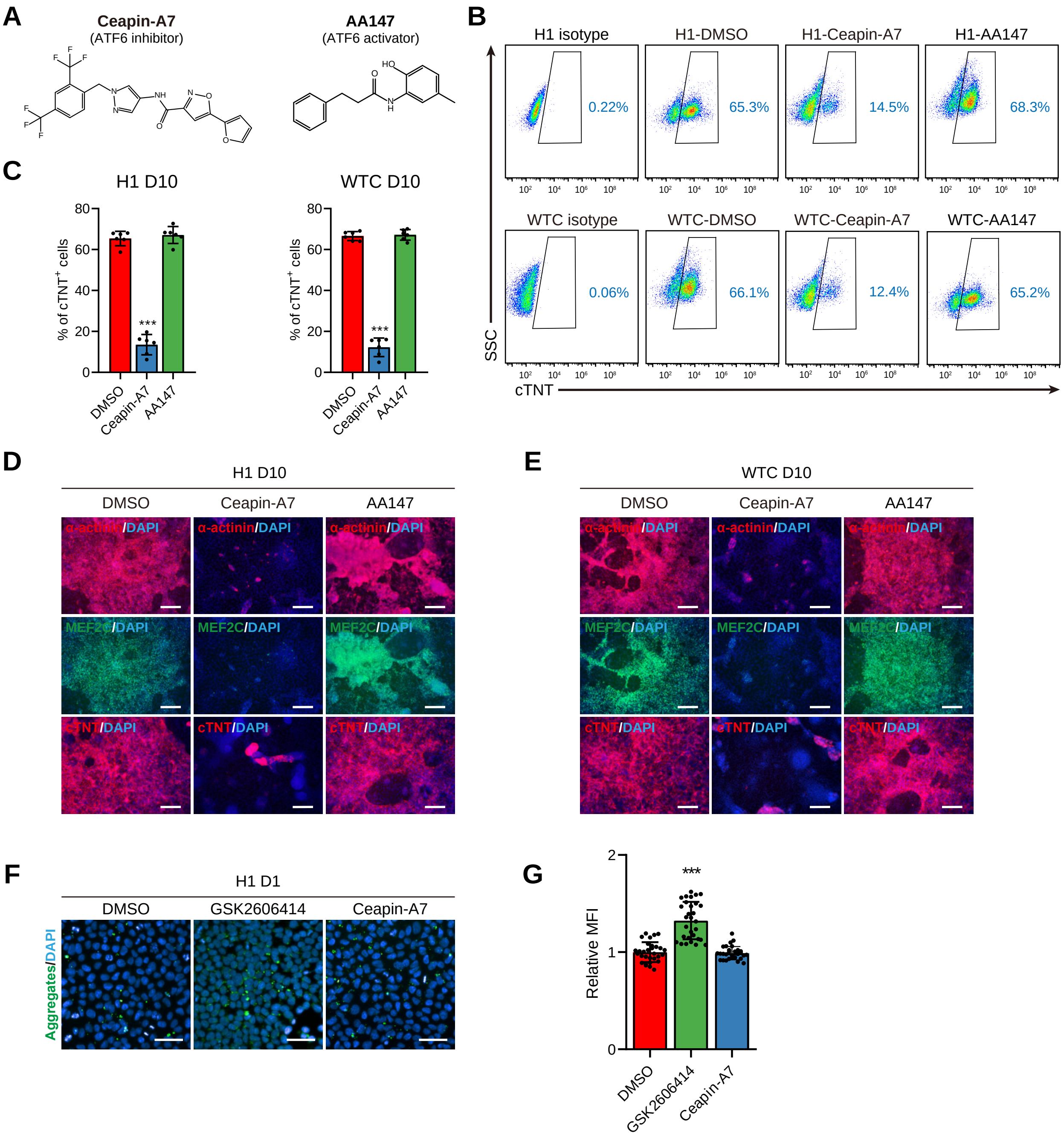

Figure S3. Effects of ATF6 inhibition or activation on CM differentiation

A, Chemical structure of the ATF6 inhibitor Ceapin-A7 and the ATF6 activator AA147.

B and **C**, Representative (**B**) and quantitative (**C**) flow cytometric analysis of cTNT^{+} cells in H1 hESC- or WTC hiPSC-derived D10 cultures treated with DMSO, Ceapin-A7, or AA147 during differentiation. n = 6 biologically independent experiments. ***P<0.001 vs. DMSO.

D and **E**, Immunofluorescence analysis of the CM markers α-actinin, MEF2C, and cTNT in H1 hESC (**D**)- or WTC hiPSC (**E**)-derived D10 cultures treated with DMSO, Ceapin-A7, or AA147 during differentiation. Scale bars, 200 μm.

F and **G**, Representative (**F**) and quantitative (**G**) immunofluorescence analysis of the protein aggregates in H1 hESC-derived D1 cells treated with DMSO, GSK2606414, or Ceapin-A7 during differentiation. n = 3 biologically independent experiments, 10 fields of view per experiment. ***P<0.001 vs. DMSO. Scale bars, 50 μm.

Data represent mean ± SD. Statistical significance was determined by one-way ANOVA with a post-hoc Tukey test.

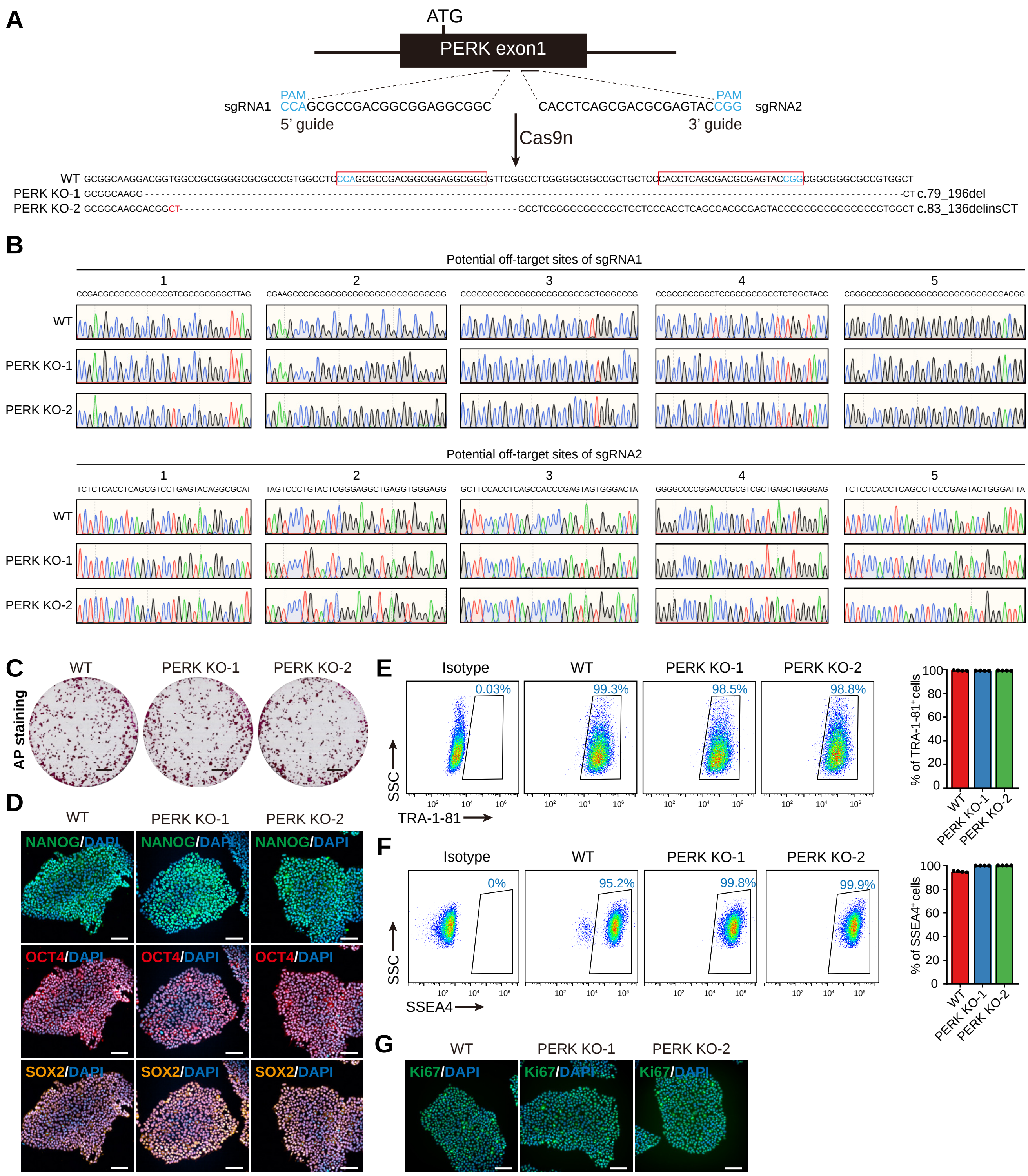

Figure S4. Generation of PERK knockout hESCs

A, Schematic of CRISPR/Cas9-mediated knockout (KO) of PERK and genotyping of two PERK KO hESC clones by Sanger sequencing.

B, Sanger sequencing of the potential off-target sites of sgRNA1 or sgRNA2 predicated by Cas-OFFinder in WT and PERK KO hESCs.

C, Alkaline phosphatase (AP) staining analysis of WT and PERK KO hESCs. Scale bar, 5 mm.

D, Immunofluorescence analysis of the pluripotency markers NANOG, OCT4, and SOX2 in WT and PERK KO hESCs. Scale bars, 100 μm.

E and **F**, Representative (left) and quantitative (right) flow cytometry analysis of pluripotency markers TRA-1-81 (**E**) and SSEA4 (**F**) in WT and PERK KO hESCs. n = 4 biologically independent experiments.

G, Immunofluorescence analysis of the proliferation marker Ki67 in WT and PERK KO hESCs. Scale bars, 100 μm.

Data represent mean ± SD. Statistical significance was determined by one-way ANOVA with a post-hoc Tukey test.

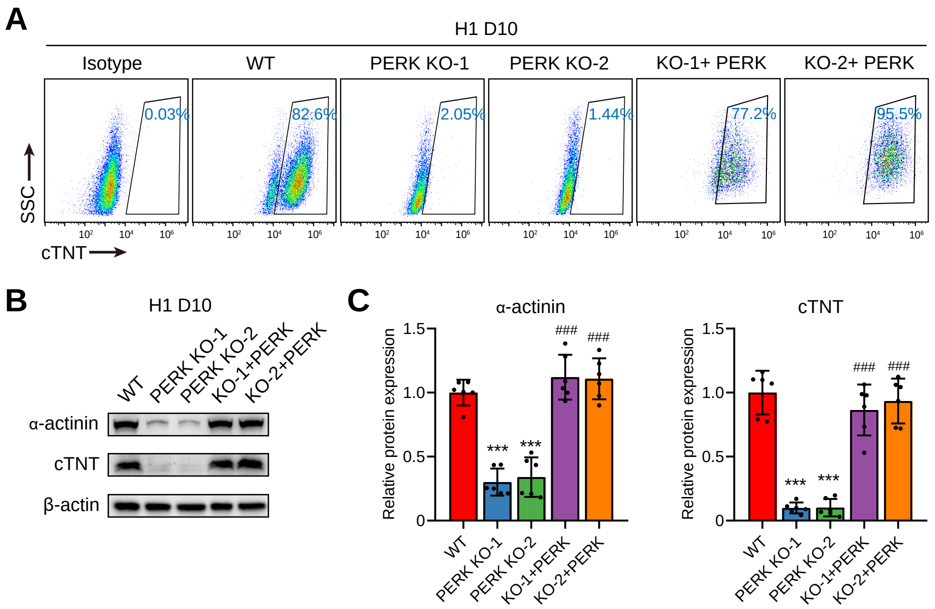

Figure S5. PERK is essential for cardiogenesis of H1 hESCs

A, Representative flow cytometry analysis of cTNT expression in D10 cultures differentiated from WT, PERK KO, or PERK re-expressed H1 hESCs.

B and **C**, Representative (**B**) and quantitative (**C**) immunoblot analysis of the CM markers α-actinin and cTNT in D10 cultures differentiated from WT, PERK KO, or PERK re-expressed H1 hESCs. β-actin was used as a loading control. n = 6 biologically independent experiments. $^{***}P$<0.001 vs. ESC; $^{\#\#\#}P$ < 0.001 vs. the corresponding PERK KO clone.

Data represent mean ± SD. Statistical significance was determined by one-way ANOVA with a post-hoc Tukey test.

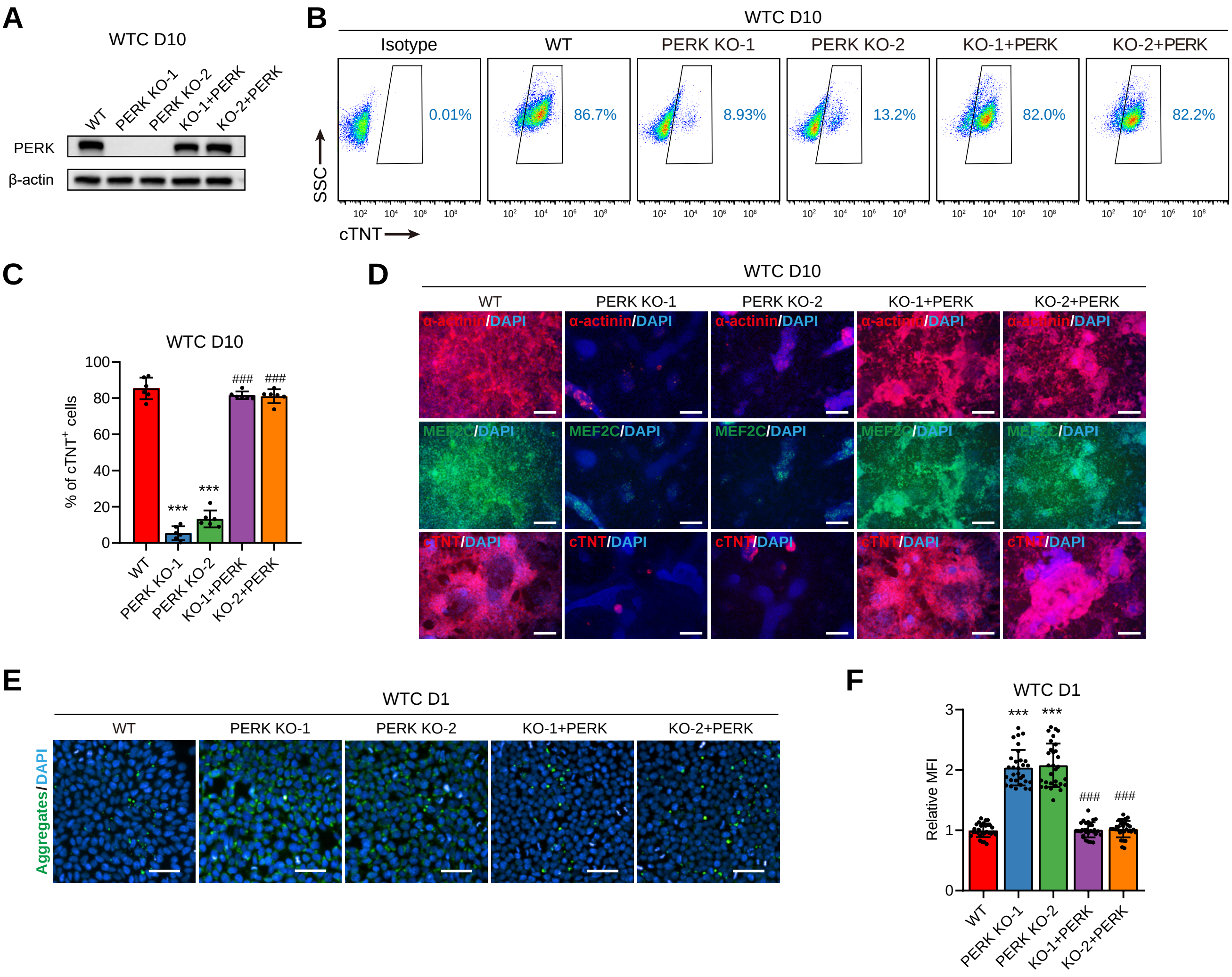

Figure S6. PERK is essential for cardiogenesis of WTC hiPSCs

A, Immunoblot analysis of PERK in WT, PERK KO, and PERK re-expressed WTC hiPSC clones.

B and **C**, Representative (**B**) and quantitative (**C**) flow cytometric analysis of cTNT expression in D10 cultures differentiated from WT, PERK KO, or PERK re-expressed WTC hiPSCs. n = 6 biologically independent experiments. ****P*<0.001 vs. WT; $^{\#\#\#}$*P*<0.001 vs. the corresponding PERK KO clone.

D, Immunofluorescence analysis of CM markers in D10 cultures differentiated from WT, PERK KO, and PERK re-expressed WTC hiPSCs. Scale bars, 200 μm.

E and **F**, Representative (**E**) and quantitative (**F**) immunofluorescence analysis of the protein aggregates in D1 cultures differentiated from WT, PERK KO, and PERK re-expressed WTC hiPSCs. n = 3 biologically independent experiments, 10 fields of view per experiment. ****P*<0.001 vs. WT; $^{\#\#\#}$*P*<0.001 vs. the corresponding PERK KO clone. Scale bars, 50 μm.

Data represent mean ± SD. Statistical significance was determined by one-way ANOVA with a post-hoc Tukey test.

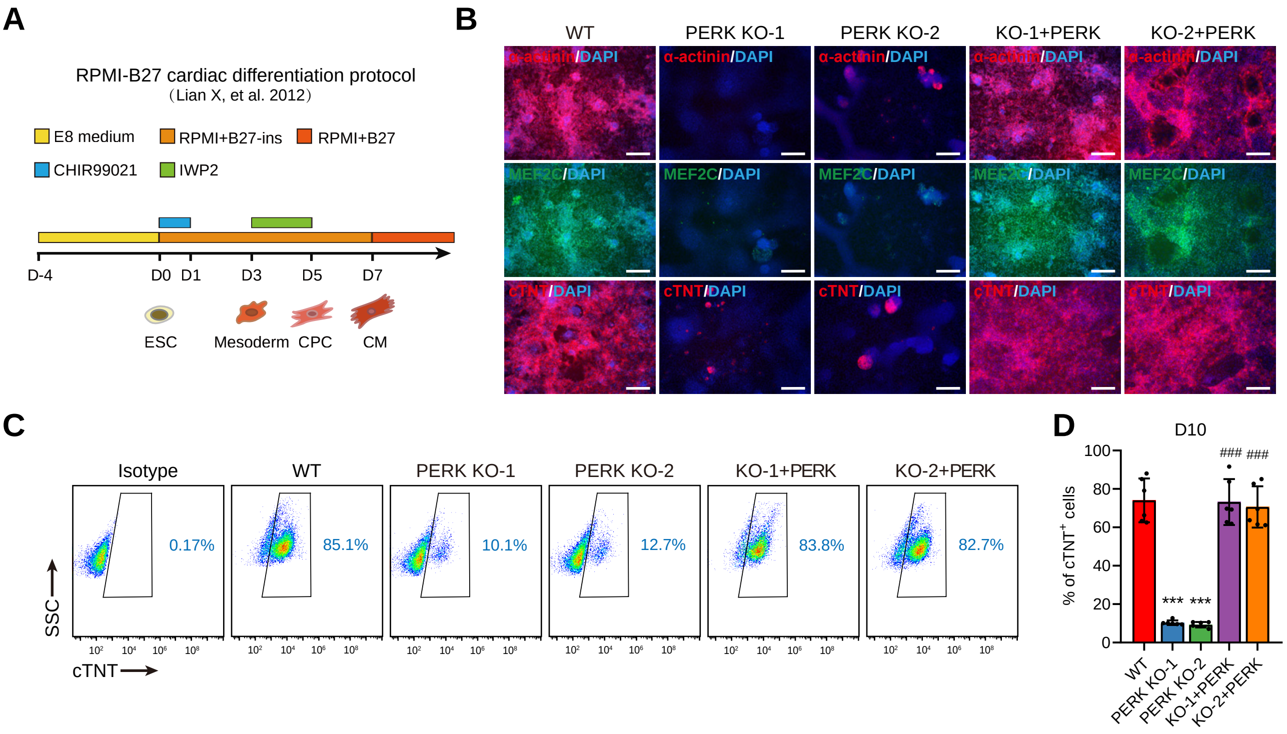

Figure S7. PERK is essential for cardiogenesis in an alternative RPMI-B27 cardiac differentiation protocol

A, Schematic of the RPMI-B27 cardiac differentiation protocol.

B, Immunofluorescence analysis of CM markers in D10 cultures differentiated from WT, PERK KO, and PERK re-expressed H1 hESCs using the RPMI-B27 protocol. Scale bars, 200 μm.

C and **D**, Representative (**C**) and quantitative (**D**) flow cytometric analysis of cTNT expression in D10 cultures differentiated from WT, PERK KO, or PERK re-expressed H1 hESCs using the RPMI-B27 protocol. n = 6 biologically independent experiments. ***P<0.001 vs. WT; $^{###}P$<0.001 vs. the corresponding PERK KO clone.

Data represent mean ± SD. Statistical significance was determined by one-way ANOVA with a post-hoc Tukey test.

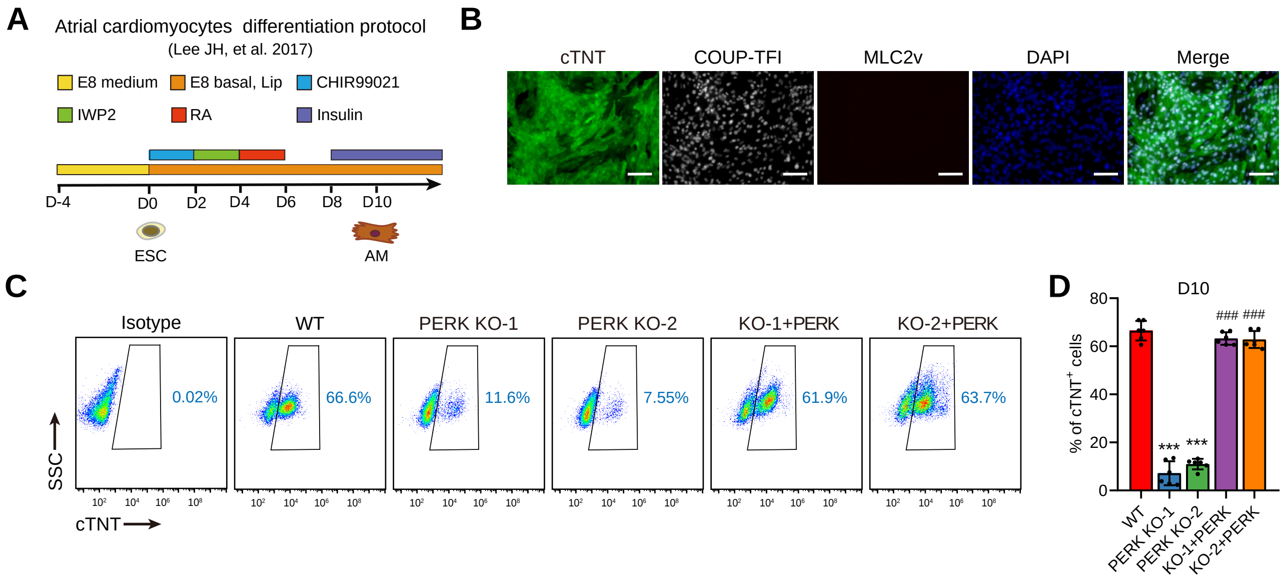

Figure S8. PERK is essential for atrial CM differentiation

A, Schematic of the atrial CM differentiation protocol.

B, Immunofluorescence analysis of the general CM marker cTNT, ventricular CM marker MLC2v, and atrial CM marker COUP-TFI in D10 cultures differentiated from H1 hESCs using the protocol descripted in A. Scale bars, 100 μm.

C and **D**, Representative (**C**) and quantitative (**D**) flow cytometric analysis of cTNT expression in D10 cultures differentiated from WT, PERK KO, or PERK re-expressed H1 hESCs using the protocol shown in A. n = 6 biologically independent experiments. ****P*<0.001 vs. WT; $^{\#\#\#}$*P*<0.001 vs. the corresponding PERK KO clone.

Data represent mean ± SD. Statistical significance was determined by one-way ANOVA with a post-hoc Tukey test.

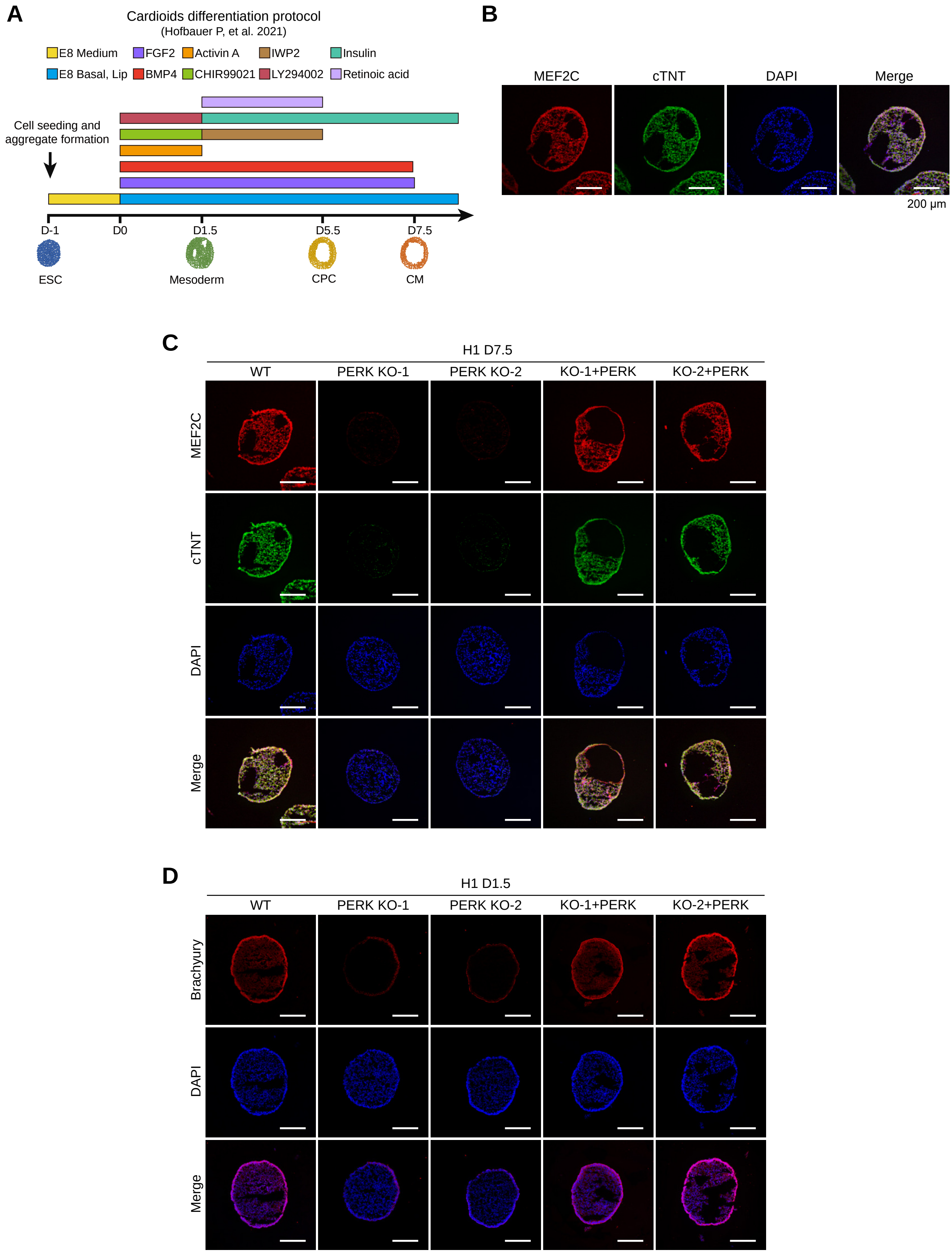
A
Cardioids differentiation protocol
(Hofbauer P, et al. 2021)
E8 Medium
FGF2
Activin A
IWP2
Insulin
E8 Basal, Lip
BMP4
CHIR99021
LY294002
Retinoic acid
Cell seeding and aggregate formation
D-1
D0
D1.5
D5.5
D7.5
ESC
Mesoderm
CPC
CM
B
MEF2C
cTNT
DAPI
Merge
200 μm
C
H1 D7.5
WT
PERK KO-1
PERK KO-2
KO-1+PERK
KO-2+PERK
MEF2C
cTNT
DAPI
Merge
D
H1 D1.5
WT
PERK KO-1
PERK KO-2
KO-1+PERK
KO-2+PERK
Brachyury
DAPI
Merge

Figure S9. PERK is essential for cardioids differentiation

A, Schematic of the cardioids differentiation protocol.

B, Immunofluorescence analysis of the CM markers MEF2C and cTNT in D7.5 cardioids differentiated from H1 hESCs using the protocol descripted in A. Scale bars, 200 μm.

C, Immunofluorescence analysis of the CM markers MEF2C and cTNT in D7.5 cardioids differentiated from WT, PERK KO, and PERK re-expressed H1 hESCs using the protocol shown in A. Scale bars, 200 μm.

D, Immunofluorescence analysis of the mesoderm marker Brachyury in D1.5 cardioids differentiated from WT, PERK KO, and PERK re-expressed H1 hESCs using the protocol shown in A. Scale bars, 200 μm.

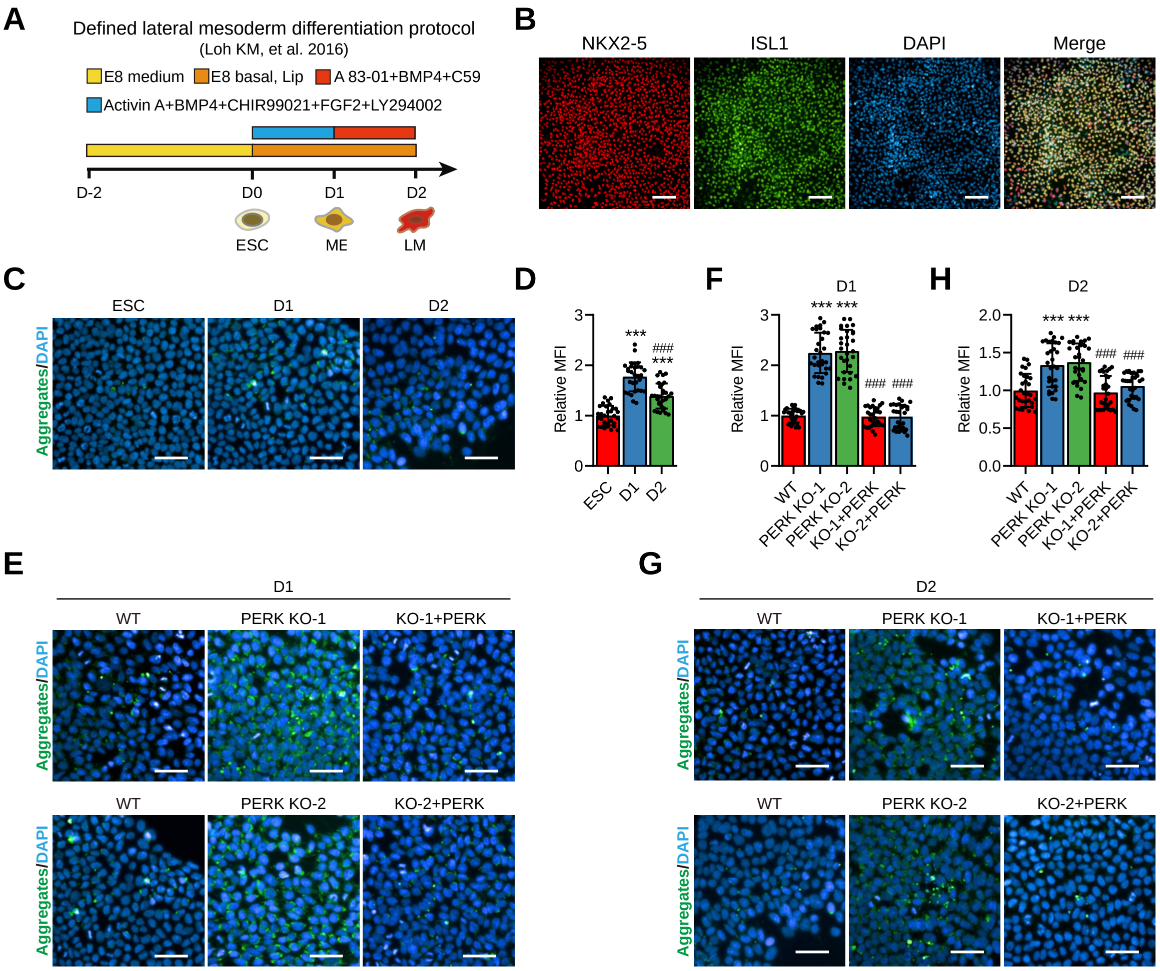

Figure S10. PERK is essential for lateral mesoderm differentiation

A, Schematic of the lateral mesoderm differentiation protocol. LM, lateral mesoderm.

B, Immunofluorescence analysis of LM markers NKX2-5 and ISL1 in D2 cultures differentiated from H1 hESCs using the protocol shown in A. Scale bars, 100 μm.

C and **D**, Representative (**C**) and quantitative (**D**) immunofluorescence analysis of the protein aggregates at each stage of LM differentiation of H1 hESCs. n = 3 biologically independent experiments, 10 fields of view per experiment. $^{***}P$<0.001 vs. ESC; $^{\#\#\#}P$<0.001 vs. D1. Scale bars, 50 μm.

E and **F**, Representative (**E**) and quantitative (**F**) immunofluorescence analysis of the protein aggregates in D1 ME cells differentiated from WT, PERK KO, and PERK re-expressed H1 hESCs. n = 3 biologically independent experiments, 10 fields of view per experiment. $^{***}P$<0.001 vs. WT; $^{\#\#\#}P$<0.001 vs. the corresponding PERK KO clone. Scale bars, 50 μm.

G and **H**, Representative (**G**) and quantitative (**H**) immunofluorescence analysis of the protein aggregates in D2 LM cells differentiated from WT, PERK KO, and PERK re-expressed H1 hESCs. n = 3 biologically independent experiments, 10 fields of view per experiment. $^{***}P$<0.001 vs. WT; $^{\#\#\#}P$<0.001 vs. the corresponding PERK KO clone. Scale bars, 50 μm.

Data represent mean ± SD. Statistical significance was determined by one-way ANOVA with a post-hoc Tukey test.

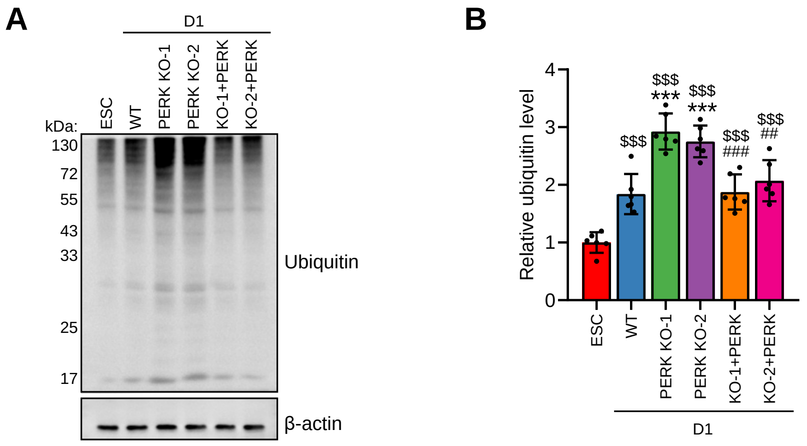

Figure S11. PERK affects the overall protein ubiquitination level of D1 ME cells

A and **B**, Representative (**A**) and quantitative (**B**) immunoblot analysis of the ubiquitin in D1 ME cells differentiated from WT, PERK KO, and PERK re-expressed H1 hESCs. β-actin was used as a loading control. n = 6 biologically independent experiments. $^{\$\$\$}P<0.001$ vs. WT ESC; $^{***}P<0.001$ vs. WT D1; $^{\#\#}P<0.01$, $^{\#\#\#}P<0.001$ vs. the corresponding PERK KO clone at D1.

Data represent mean ± SD. Statistical significance was determined by one-way ANOVA with a post-hoc Tukey test.

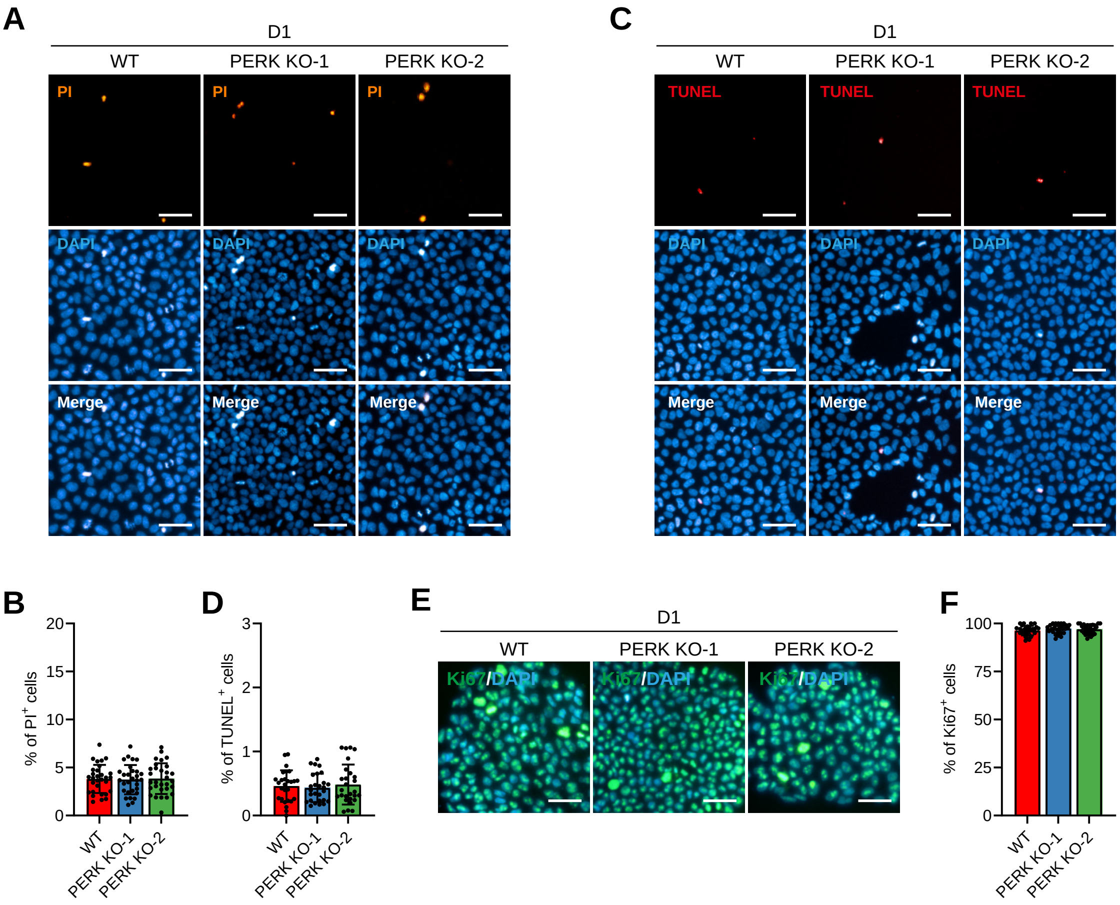

Figure S12. PERK is dispensable for cell death and proliferation of D1 ME cells

A and **B**, Representative (**A**) and quantitative (**B**) immunofluorescence analysis of the propidium iodide (PI) in D1 cultures differentiated from WT and PERK KO hESCs. n = 3 biologically independent experiments, 10 fields of view per experiment. Scale bars, 50 μm.

C and **D**, Representative (**C**) and quantitative (**D**) immunofluorescence analysis of the TUNEL in D1 cultures differentiated from WT and PERK KO hESCs. n = 3 biologically independent experiments, 9 fields of view per experiment. Scale bars, 50 μm.

E and **F**, Representative (**E**) and quantitative (**F**) immunofluorescence analysis of the proliferation marker Ki67 in D1 cultures differentiated from WT and PERK KO hESCs. n = 3 biologically independent experiments, 10 fields of view per experiment. Scale bars, 50 μm.

Data represent mean ± SD. Statistical significance was determined by one-way ANOVA with a post-hoc Tukey test.

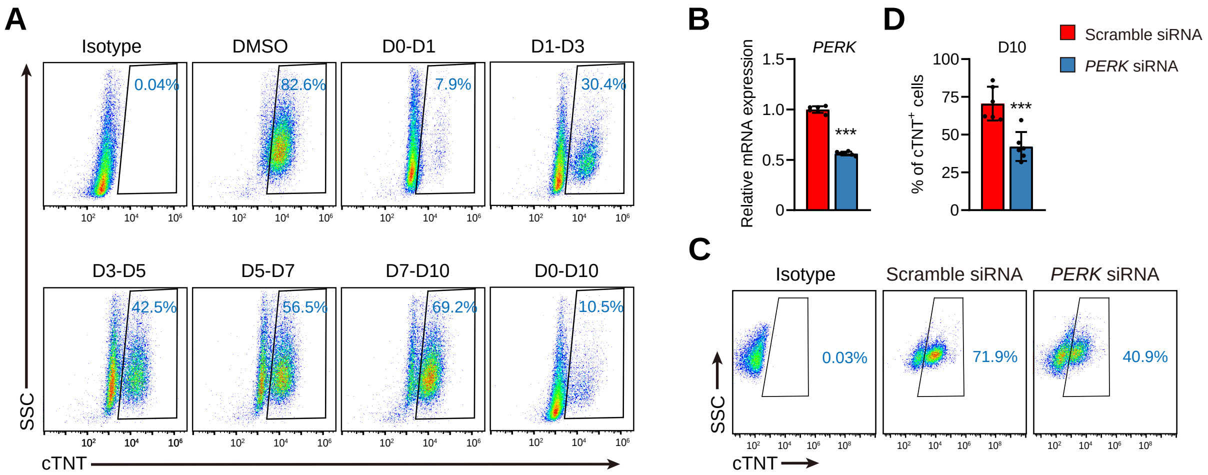

Figure S13. Stage specific effect of PERK depletion on CM differentiation

A, Representative flow cytometry analysis of cTNT expression in H1 hESCs-derived D10 cultures treated with GSK2606414 at various time windows during differentiation.

B, RT-qPCR analysis of PERK in H1 hESCs-derived D2 cultures 6 hours after transfected with the Scramble or PERK siRNA at D1. n = 6 biologically independent experiments. ****P*<0.001 vs. Scramble siRNA.

C and **D**, Representative (**C**) and quantitative (**D**) flow cytometric analysis of cTNT^{+} cells in H1 hESCs-derived D10 cultures transfected with Scramble or PERK siRNA at D2. n = 6 biologically independent experiments. ****P*<0.001 vs. Scramble siRNA.

Data represent mean ± SD. Statistical significance was determined by unpaired two-tailed t-tests.

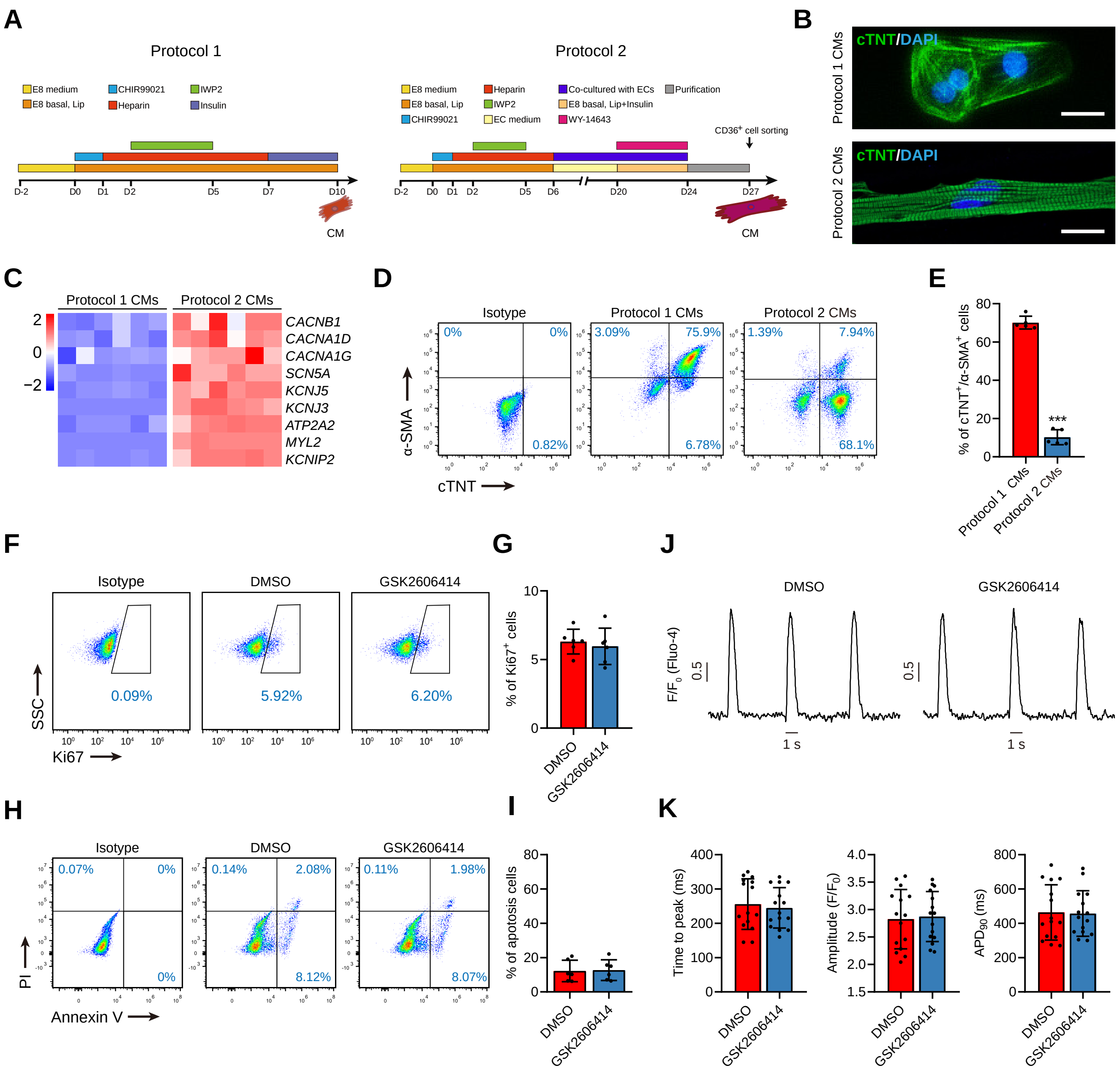

Figure S14. Effect of PERK depletion on CMs with advanced maturity

A, Schematic of the two protocols to obtain CMs.

B, Immunofluorescence analysis of cTNT in CMs differentiated by protocol 1 or protocol 2. scale bars, 20 μm.

C, Heatmap showing the relative expression level of maturation marker genes in CMs differentiated by protocol 1 and protocol 2 as determined by RT-qPCR. n = 6 biologically independent experiments.

D and **E**, Representative (**D**) and quantitative (**E**) flow cytometric analysis of cTNT and α-SMA in CMs differentiated by protocol 1 and protocol 2. n = 6 biologically independent experiments. ****P*<0.001 vs. protocol 1 CMs.

F and **G**, Representative (**F**) and quantitative (**G**) flow cytometric analysis of the proliferation marker Ki67 in protocol 2 CMs treated with DMSO or GSK2606414. n = 6 biologically independent experiments.

H and **I**, Representative (**H**) and quantitative (**I**) flow cytometric analysis of Annexin V and PI in CMs from protocol 2 treated with DMSO or GSK2606414. n = 6 biologically independent experiments.

J, Representative traces of Ca^{2+} transients in CMs from protocol 2 treated with DMSO or GSK2606414.

K, Quantification of time to peak (left panel), amplitude (middle panel) and AP duration at 90% repolarization (APD90, right panel) in protocol 2 CMs treated with DMSO or GSK2606414. n = 15 cells from 3 biologically independent experiments. APD, action potential duration.

Data represent mean ± SD. Statistical significance was determined by unpaired two-tailed t-tests.

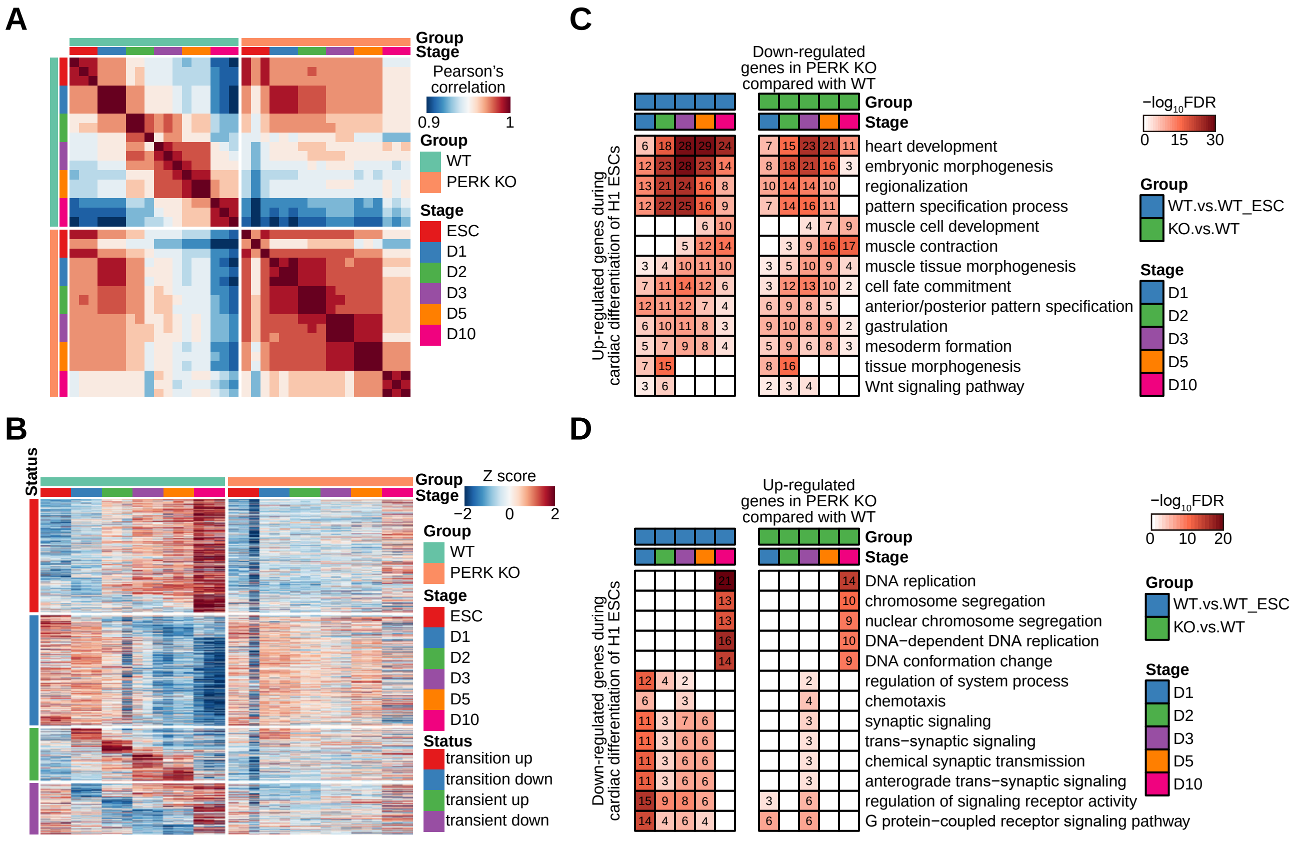

Figure S15. Depletion of PERK disrupt genome-wide transcriptional signatures of developing CMs

A, Pearson's correlation analysis of all samples in WT and PERK KO hESCs during CM differentiation.

B, Time course RNA-seq data analysis using ImpluseDE2 showing the dynamic gene expression pattern of WT and PERK KO hESCs during CM differentiation.

C, Gene ontology (GO) analysis reveal that genes that up-regulated during CM differentiation compared to undifferentiated hESCs (left panel) enrich similar GO terms with the down-regulated genes in PERK KO cells compared to the WT control (right panel).

D, GO analysis reveal that genes that down-regulated during CM differentiation compared to undifferentiated hESCs (left panel) enrich similar GO terms with the up-regulated genes in PERK KO cells compared to the WT control (right panel).

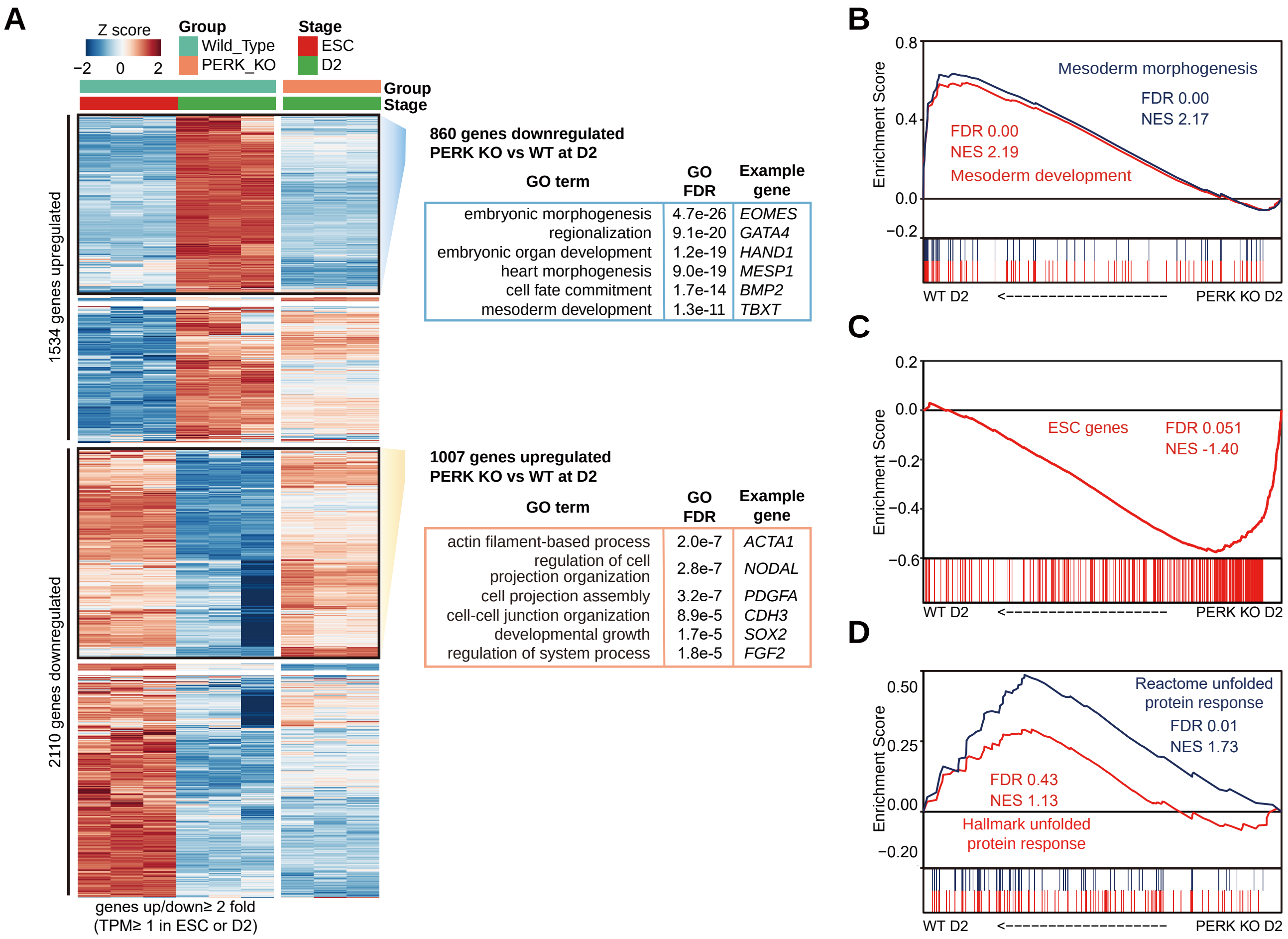

Figure S16. PERK KO hESCs fail to activate the mesoderm transcriptional program during cardiac specification

A, Heatmap showing up- and down-regulated genes in WT and PERK KO mesoderm cells (D2), as compared to WT ESCs determined by RNA-seq. GO analysis of genes deregulated by at least 2-fold in PERK KO mesoderm cells as compared to WT mesoderm cells are presented in the right panel.

B-D, Gene set enrichment analysis of the RNA-seq data from WT and PERK KO mesoderm cells (D2). Gene sets from the GO term ‘mesoderm development’ and ‘mesoderm morphogenesis’, the ESC-enriched genes, as well as the Reactome Pathways term ‘unfolded protein response’ and Hallmark Gene Sets term ‘unfolded protein response’ are used.

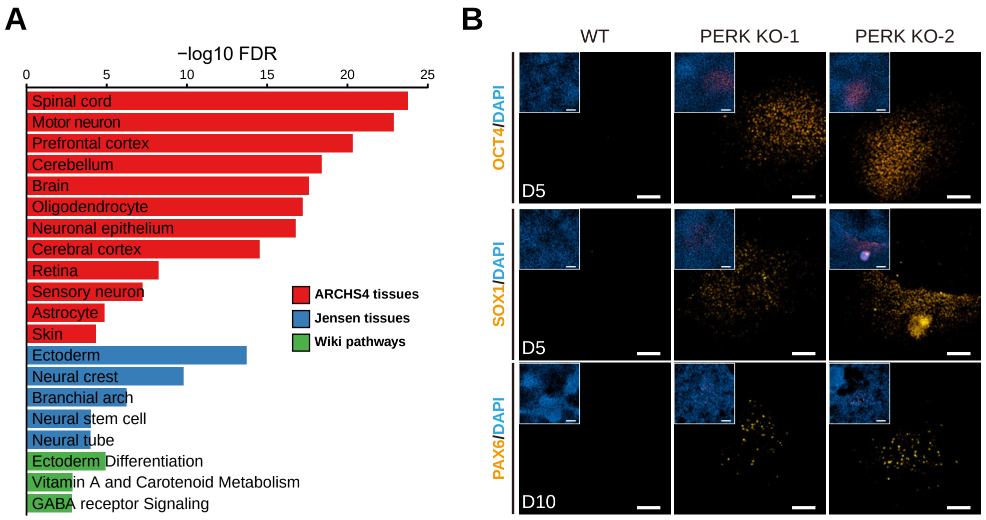

Figure S17. PERK KO hESCs retains pluripotency and differentiate into ectoderm lineage in ME-inducing conditions

A, Tissue enrichment analysis of the top 500 up-regulated genes in PERK-KO vs WT at D10 using Enricher tools.

B, Immunofluorescence analysis of the pluripotent marker OCT4 and the ectoderm markers SOX1 and PAX6 in D5 (for OCT4 and SOX1) or D10 (for PAX6) cultures derived from WT and PERK KO hESCs during CM differentiation. Insets show image at lower magnification to provide an overall view. Scale bars, 200 μm.

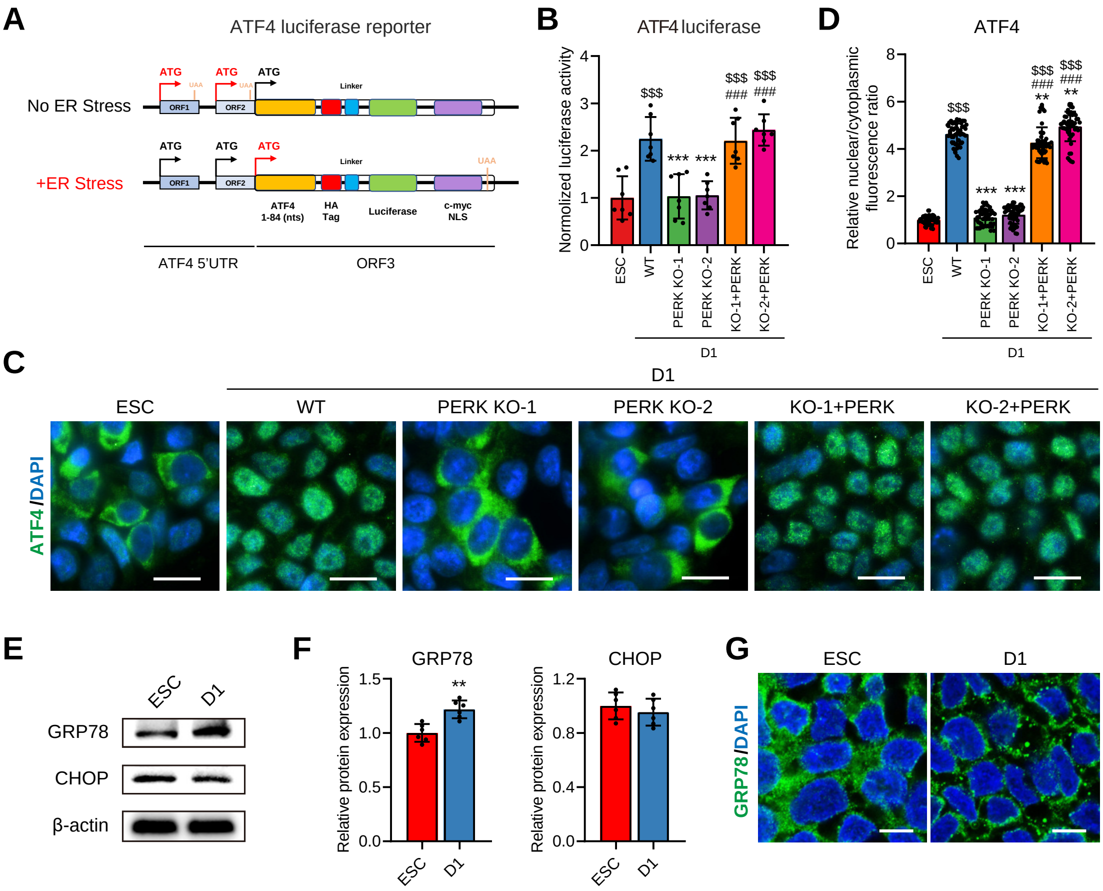

Figure S18. ATF4 and GRP78 are activated in D1 ME cells

A, Schematic of the ATF4 luciferase reporter construct. Luciferase activity indicates the ATF4 mRNA translation rate, which is regulated by the upstream open reading frames (ORFs).

B, Quantitative analysis of ATF4-luciferase activity in D1 ME cells differentiated from WT, PERK KO, or PERK re-expressed H1 hESCs. n = 6 biologically independent experiments. $^{\$\$\$}P<0.001$ vs. WT ESC; $^{***}P<0.001$ vs. WT D1; $^{\#\#\#}P<0.001$ vs. the corresponding PERK KO clone at D1.

C and **D**, Representative (**C**) and quantitative (**D**) immunofluorescence analysis of the subcellular distribution of ATF4 in D1 ME cells differentiated from WT, PERK KO, and PERK re-expressed H1 hESCs. $^{\$\$\$}P<0.001$ vs. WT ESC; $^{**}P<0.01$, $^{***}P<0.001$ vs. WT D1; $^{\#\#\#}P<0.001$ vs. the corresponding PERK KO clone at D1. Scale bars, 20 μm.

E and **F**, Representative (**E**) and quantitative (**F**) immunoblot analysis of GRP78 and CHOP in ESC and D1 cells. β-actin was used as a loading control. n = 6 biologically independent experiments. $^{**}P<0.01$ vs. ESC.

G, Immunofluorescence analysis of GRP78 in ESC and D1 cells. Scale bars, 10 μm.

Data represent mean ± SD. Statistical significance was determined by one-way ANOVA with a post-hoc Tukey test (**B** and **D**) and unpaired two-tailed *t*-test (**F**).

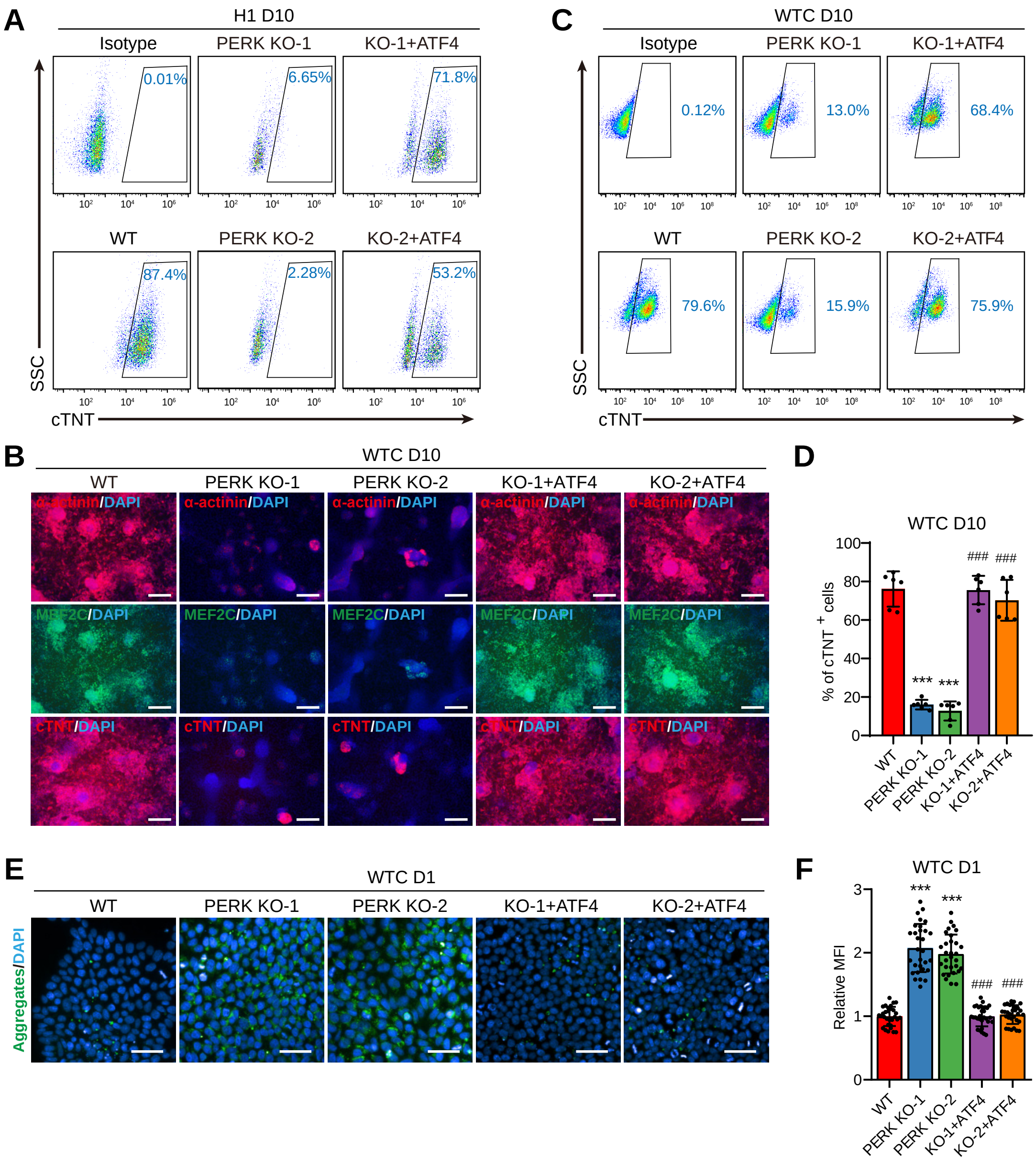

Figure S19. ATF4 activation rescues PERK KO-induced CM differentiation defect

A, Representative flow cytometry analysis of cTNT expression in D10 cultures differentiated from WT, PERK KO, or ATF4-overexpressed PERK KO H1 hESCs.

B, Immunofluorescence analysis of CM markers in D10 cultures differentiated from WT, PERK KO, and ATF4-overexpressed PERK KO WTC hiPSCs. Scale bars, 200 μm.

C and **D**, Representative (**C**) and quantitative (**D**) flow cytometry analysis of cTNT expression in D10 cultures differentiated from WT, PERK KO, or ATF4-overexpressed PERK KO WTC hiPSCs. n = 6 biologically independent experiments. ***P<0.001 vs. WT; $^{###}P$<0.001 vs. the corresponding PERK KO clone.

E and **F**, Representative (**E**) and quantitative (**F**) immunofluorescence analysis of the protein aggregates in D1 cultures differentiated from WT, PERK KO, or ATF4-overexpressed PERK KO WTC hiPSCs. n = 3 biologically independent experiments, 10 fields of view per experiment. Scale bars, 50 μm. ***P<0.001 vs. WT; $^{###}P$<0.001 vs. the corresponding PERK KO clone. Data represent mean ± SD. Statistical significance was determined by one-way ANOVA with a post-hoc Tukey test.

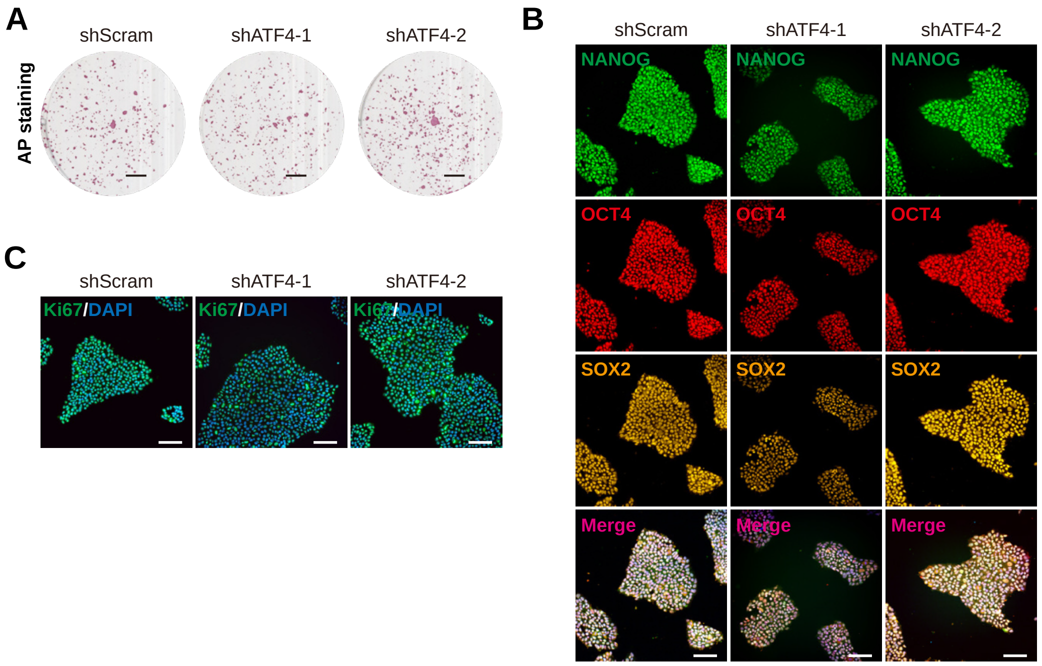

Figure S20. *ATF4* knockdown does not affect self-renewal of H1 hESCs

A, AP staining analysis of shScram control and *ATF4* knockdown (KD) H1 hESCs. shATF4-1 and shATF4-2 represent two independent *ATF4* shRNAs. Scale bar, 5mm.

B and **C**, Immunofluorescence analysis of the pluripotency markers (**B**) and the proliferation marker Ki67 (**C**) in shScram control and *ATF4* KD H1 hESCs. Scale bars, 100 μm.

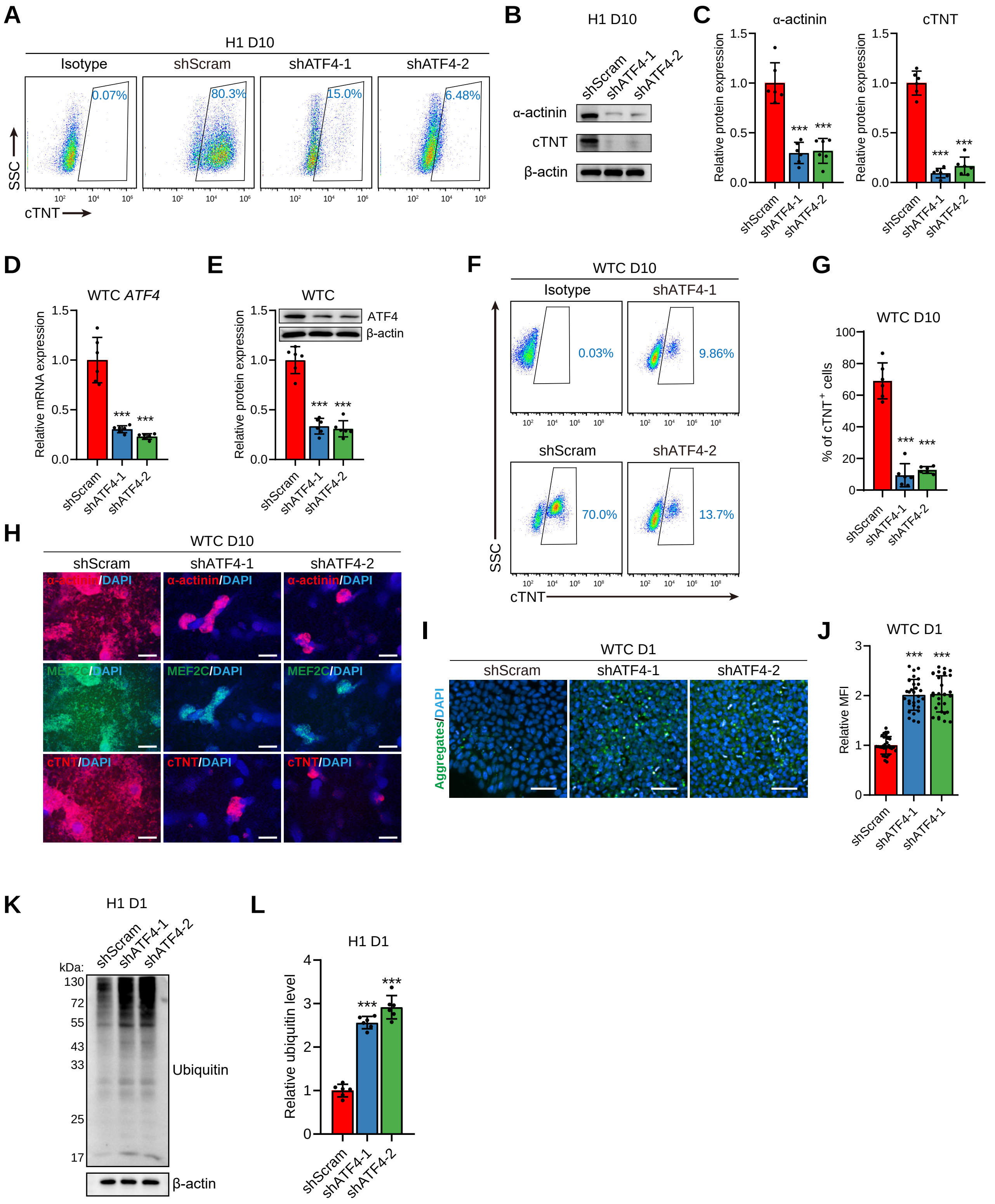
A
H1 D10
Isotype
shScram
shATF4-1
shATF4-2
0.07%
80.3%
15.0%
6.48%
SSC
cTNT
B
H1 D10
shScram
shATF4-1
shATF4-2
α-actinin
cTNT
β-actin
C
α-actinin
cTNT
Relative protein expression
D
WTC ATF4
Relative mRNA expression
E
WTC
ATF4
β-actin
Relative protein expression
F
WTC D10
Isotype
shATF4-1
0.03%
9.86%
shScram
shATF4-2
70.0%
13.7%
SSC
cTNT
G
WTC D10
% of cTNT+ cells
H
WTC D10
shScram
shATF4-1
shATF4-2
α-actinin/DAPI
MEF2C/DAPI
cTNT/DAPI
I
WTC D1
shScram
shATF4-1
shATF4-2
Aggregates/DAPI
J
WTC D1
Relative MFI
shScram
shATF4-1
shATF4-1
K
H1 D1
shScram
shATF4-1
shATF4-2
kDa:
130
72
55
43
33
25
17
Ubiquitin
β-actin
L
H1 D1
Relative ubiquitin level
***

Figure S21. *ATF4* knockdown impairs CM differentiation

A, Representative flow cytometry analysis of cTNT expression in D10 cultures differentiated from the shScram control and *ATF4* KD H1 hESCs. shATF4-1 and shATF4-2 represent two independent ATF4 shRNAs.

B and **C**, Representative (**B**) and quantitative (**C**) immunoblot analysis of the CM markers α-actinin and cTNT in D10 cultures differentiated from shScram control and *ATF4* KD H1 hESCs. n = 6 biologically independent experiments. ****P*<0.001 vs. shScram.

D and **E**, RT-qPCR (**D**) and immunoblot (**E**) analysis of ATF4 in D1 ME cells differentiated from the shScram control and *ATF4* KD WTC hiPSCs. n = 6 biologically independent experiments. ****P*<0.001 vs. shScram.

F and **G**, Representative (**F**) and quantitative (**G**) flow cytometric analysis of cTNT expression in D10 cultures differentiated from the shScram control and *ATF4* KD WTC hiPSCs. n = 6 biologically independent experiments. ****P*<0.001 vs. shScram.

H, Immunofluorescence analysis of CM markers in D10 cultures differentiated from the shScram control and *ATF4* KD WTC hiPSCs. Scale bars, 200 μm.

I and **J**, Representative (**I**) and quantitative (**J**) immunofluorescence analysis of the protein aggregates in D1 cultures differentiated from the shScram control and *ATF4* KD WTC hiPSCs. n = 3 biologically independent experiments, 10 fields of view per experiment. Scale bars, 50 μm. ****P*<0.001 vs. shScram.

K and **L**, Representative (**K**) and quantitative (**L**) immunoblot analysis of the ubiquitin in D1 ME cells differentiated from shScram control and *ATF4* KD H1 hESCs. β-actin was used as a loading control. n =

6 biologically independent experiments. ****P*<0.001 vs. shScram.

Data represent mean ± SD. Statistical significance was determined by one-way ANOVA with a post-hoc Tukey test.

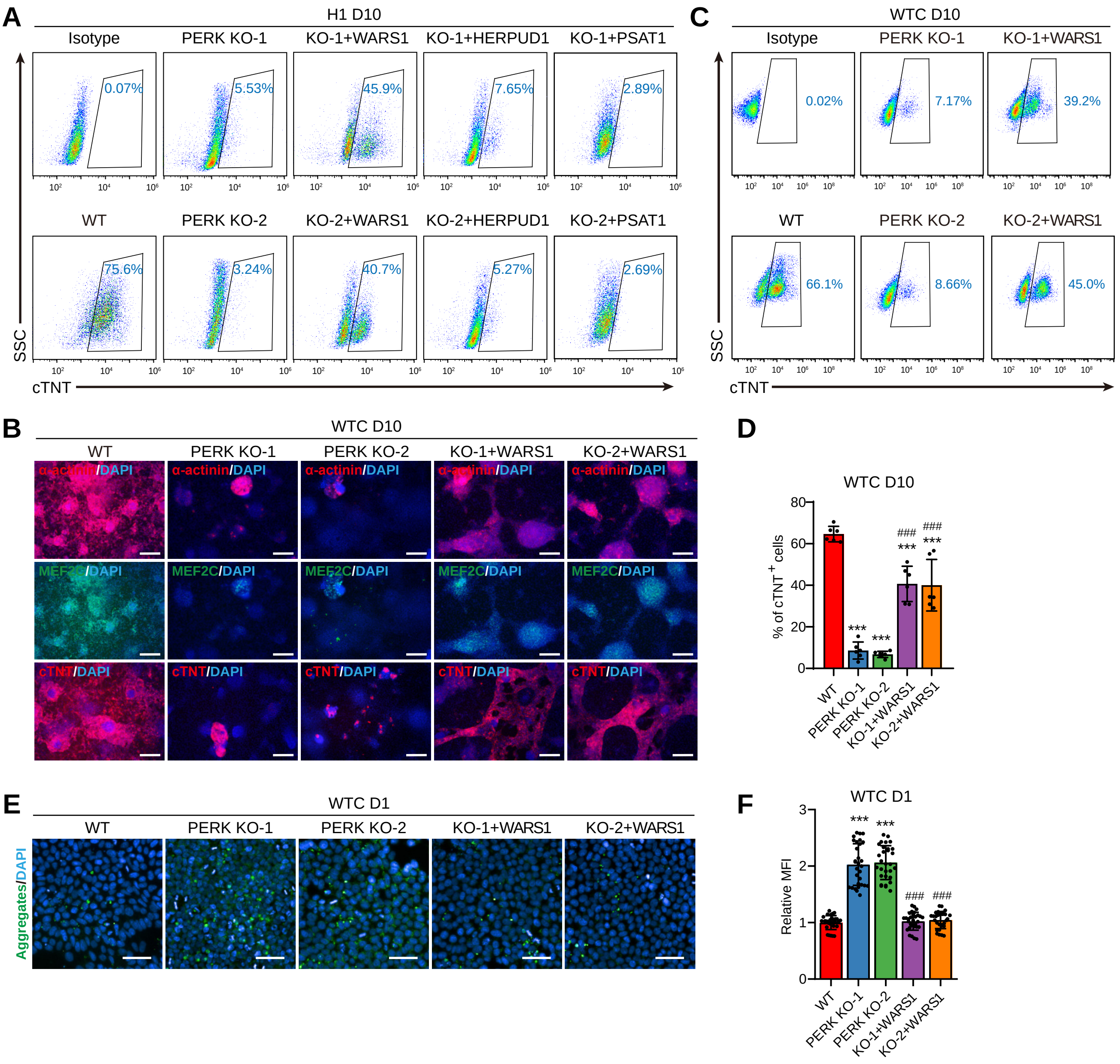

Figure S22. WARS1 activation rescues PERK KO-induced CM differentiation defect

A, Representative flow cytometry analysis of cTNT expression in D10 cultures differentiated from WT and PERK KO, as well as PERK KO H1 hESCs that receive WARS1-, HERPUD1-, or PSAT1-overexpression, respectively.

B, Immunofluorescence analysis of CM markers in D10 cultures differentiated from WT, PERK KO, and WARS1-overexpressed PERK KO WTC hiPSCs. Scale bars, 200 μm.

C and **D**, Representative (**C**) and quantitative (**D**) flow cytometric analysis of cTNT expression in D10 cultures differentiated from WT, PERK KO, or WARS1-overexpressed PERK KO WTC hiPSCs. n = 6 biologically independent experiments. ***P<0.001 vs. WT; ###P<0.001 vs. the corresponding PERK KO clone.

E and **F**, Representative (**E**) and quantitative (**F**) immunofluorescence analysis of the protein aggregates in D1 cultures differentiated from WT, PERK KO, and WARS1-overexpressed PERK KO WTC hiPSCs. n = 3 biologically independent experiments, 10 fields of view per experiment. Scale bars, 50 μm. ***P<0.001 vs. WT; ###P<0.001 vs. the corresponding PERK KO clone.

Data represent mean ± SD. Statistical significance was determined by one-way ANOVA with a post-hoc Tukey test.

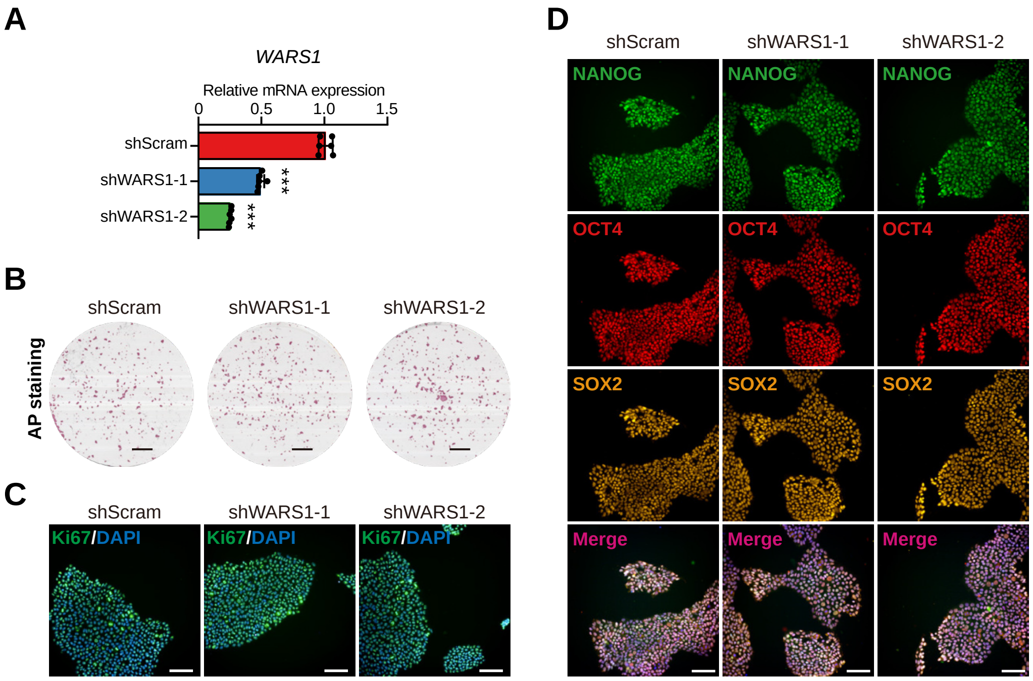

Figure S23. *WARS1* knockdown does not affect self-renewal of H1 hESCs

A, RT-qPCR analysis of *WARS1* in shScram control and *WARS1* KD ME cells at D1. shWARS1-1 and shWARS1-2 represent two independent *WARS1* shRNAs. n = 6 biologically independent experiments. ****P*<0.001 vs. shScram.

B, AP staining of shScram control and *WARS1* KD H1 hESCs. Scale bar, 5mm.

C and **D**, Immunofluorescence analysis of the pluripotency markers (**C**) and the proliferation marker Ki67 (**D**) in shScram control and *WARS1* KD H1 hESCs. Scale bars, 100 μm.

Data represent mean ± SD. Statistical significance was determined by one-way ANOVA with a post-hoc Tukey test.

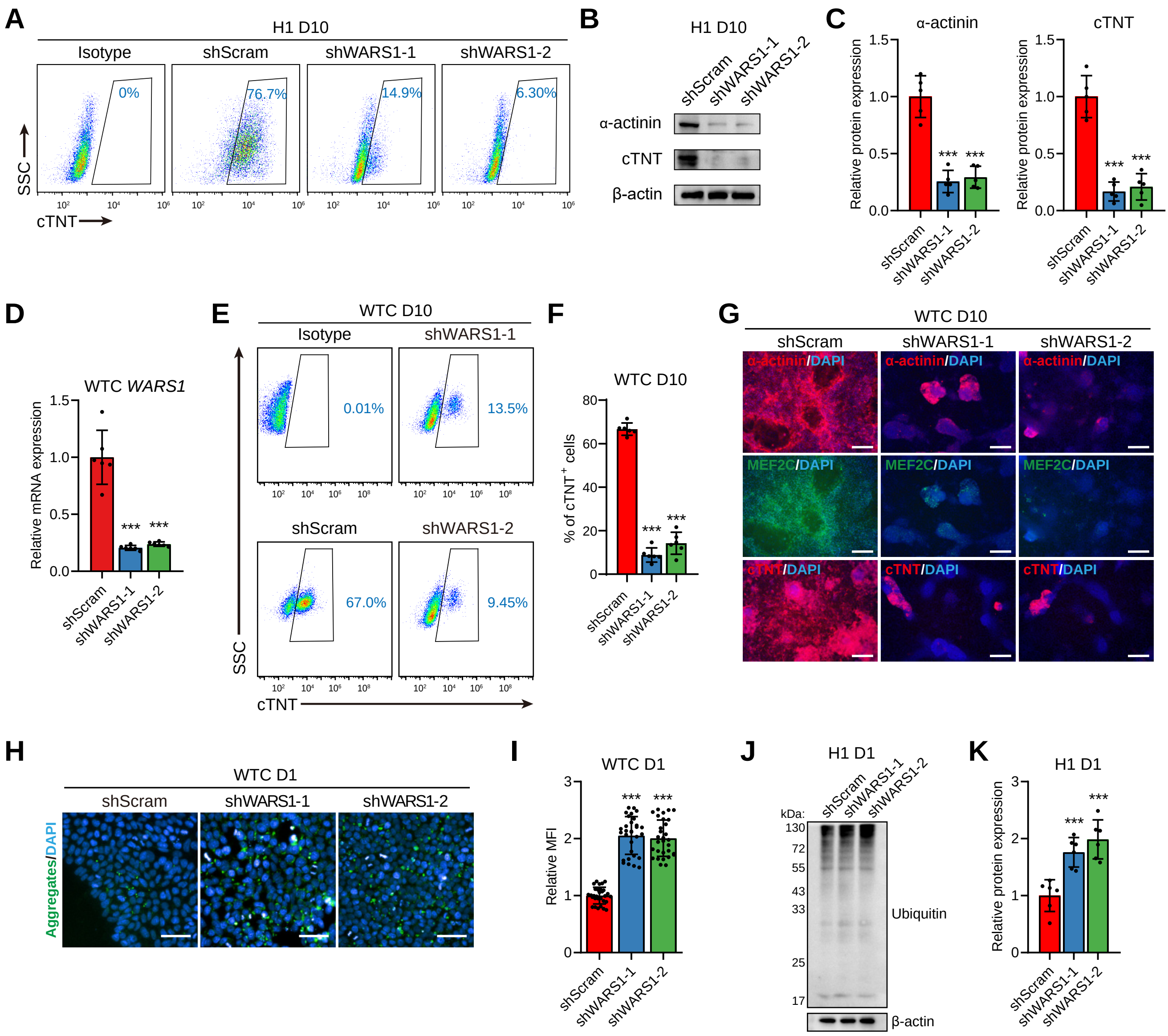

A
H1 D10
Isotype
shScram
shWARS1-1
shWARS1-2
0%
76.7%
14.9%
6.30%
SSC
cTNT
B
H1 D10
shScram
shWARS1-1
shWARS1-2
α-actinin
cTNT
β-actin
C
α-actinin
cTNT
Relative protein expression
D
WTC WARS1
Relative mRNA expression
E
WTC D10
Isotype
shWARS1-1
shScram
shWARS1-2
0.01%
13.5%
67.0%
9.45%
F
WTC D10
% of cTNT+ cells
G
WTC D10
α-actinin/DAPI
MEF2C/DAPI
cTNT/DAPI
H
WTC D1
Aggregates/DAPI
I
WTC D1
Relative MFI
J
H1 D1
kDa:
130
72
55
43
33
25
17
Ubiquitin
β-actin
K
H1 D1
Relative protein expression

Figure S24. *WARS1* knockdown impairs CM differentiation

A, Representative flow cytometry analysis of cTNT expression in D10 cultures differentiated from shScram control and *WARS1* KD H1 hESCs.

B and **C**, Representative (**B**) and quantitative (**C**) immunoblot analysis of the CM markers α-actinin and cTNT in D10 cultures differentiated from shScram control and *WARS1* KD H1 hESCs. n = 5 biologically independent experiments. ****P*<0.001 vs. shScram.

D, RT-qPCR analysis of *WARS1* in D1 ME cells differentiated from the shScram control and *WARS1* KD WTC hiPSCs. shWARS1-1 and shWARS1-2 represent two independent *WARS1* shRNAs. n = 6 biologically independent experiments. ****P*<0.001 vs. shScram.

E and **F**, Representative (**E**) and quantitative (**F**) flow cytometric analysis of cTNT expression in D10 cultures differentiated from the shScram control and *WARS1* KD WTC hiPSCs. n = 6 biologically independent experiments. ****P*<0.001 vs. shScram.

G, Immunofluorescence analysis of CM markers in D10 cultures differentiated from the shScram control and *WARS1* KD WTC hiPSCs. Scale bars, 200 µm.

H and **I**, Representative (**H**) and quantitative (**I**) immunofluorescence analysis of the protein aggregates in D1 cultures differentiated from the shScram control and *WARS1* KD WTC hiPSCs. n = 3 biologically independent experiments, 10 fields of view per experiment. Scale bars, 50 µm. ****P*<0.001 vs. shScram.

J and **K**, Representative (**J**) and quantitative (**K**) immunoblot analysis of the ubiquitin in D1 ME cells differentiated from shScram control and *WARS1* KD H1 hESCs. β-actin was used as a loading control. n = 6 biologically independent experiments. ****P*<0.001 vs. shScram.

Data represent mean ± SD. Statistical significance was determined by one-way ANOVA with a post-hoc Tukey test.

Table S1 Primer sequences used in this paper

Primer	Sequence(5'→3')	Usage
shATF4-1-F	CCGGCCACTCCAGATCATTCCTTTACTCGAG TAAAGGAATGATCTGGAGTGGTTTTTG	Construct knock down plasmid
shATF4-1-R	AATTCAAAAACCACTCCAGATCATTCCTTTA CTCGAGTAAAGGAATGATCTGGAGTGG	
shATF4-2-F	CCGGCCTCAGTGCATAAAGGAGGAACTCGA GTTCCTCCTTTATGCACTGAGGTTTTTG	
shATF4-2-R	AATTCAAAAACCTCAGTGCATAAAGGAGGA ACTCGAGTTCCTCCTTTATGCACTGAGG	
shWARS1-1-F	CCGGGTCACGGATGAGATAGTGAAACTCGA GTTTCACTATCTCATCCGTGACTTTTTG	
shWARS1-1-R	AATTCAAAAAGTCACGGATGAGATAGTGAA ACTCGAGTTTCACTATCTCATCCGTGAC	
shWARS1-2-F	CCGGCTTTGACATCAACAAGACTTTCTCGAG AAAGTCTTGTTGATGTCAAAGTTTTTG	
shWARS1-2-R	AATTCAAAAACTTTGACATCAACAAGACTTT CTCGAGAAAGTCTTGTTGATGTCAAAG	
OE-*hPERK*-F	TCCTACCCTCGTAAAGAATTCATGGAGCGCG CCATCAGC	Construct overexpression plasmid
OE-*hPERK*-R	CAGGGGAGGTGGTCTGGATCCCTACTTATCG TCGTCATCCTTGTAATCATTGCTTGGCAAAG GGCTATG	
OE-*hATF4*-F	TCCTACCCTCGTAAAGAATTCATGACCGAAA TGAGCTTCCTGA	
OE-*hATF4*-R	CAGGGGAGGTGGTCTGGATCCCTACTTATCG TCGTCATCCTTGTAATCGGGGACCCTTTTCTT CCCC	
OE-*hWARS1*-F	TCCTACCCTCGTAAAGAATTCATGCCCAACA GTGAGCCCG	
OE-*hWARS1*-R	CAGGGGAGGTGGTCTGGATCCCTACTTATCG TCGTCATCCTTGTAATCCTGAAAGTCGAAGG ACAGCTTCC	
OE-*hPSAT1*-F	TCCTACCCTCGTAAAGAATTCATGGACGCCC CCAGGCAG	
OE-*hPSAT1*-R	CAGGGGAGGTGGTCTGGATCCTCACTTATCG TCGTCATCCTTGTAATCTAGCTGATGCATCTC CAAAAATTT	
OE-*hHERPUD1*-F	TCCTACCCTCGTAAAGAATTCATGGAGTCCG AGACCGAACC	
OE-*hHERPUD1*-R	CAGGGGAGGTGGTCTGGATCCTCACTTATCG TCGTCATCCTTGTAATCGTTTGCGATGGCTG GGGG	
hSOX17-qPCR-F	GTGGACCGCACGGAATTTG	RT-qPCR
hSOX17-qPCR-R	GGAGATTCACACCGGAGTCA	
hFOXA2-qPCR-F	GGAGCAGCTACTATGCAGAGC	
hFOXA2-qPCR-R	CGTGTTCATGCCGTTCATCC	
hGATA4-qPCR-F	CGACACCCCAATCTCGATATG	

hGATA4-qPCR-R	GTTGCACAGATAGTGACCCGT
hOTX2-qPCR-F	CATGCAGAGGTCCTATCCCAT
hOTX2-qPCR-R	AAGCTGGGGACTGATTGAGAT
hPAX6-qPCR-F	TCGAAGGGCCAAATGGAGAAGAGAAG
hPAX6-qPCR-R	GGTGGGTTGTGGAATTGGTTGGTAGA
hNESTIN-qPCR-F	GGCAGCGTTGGAACAGAGG
hNESTIN-qPCR-R	CCTTCCAGGACCTGAGCGA
hOCT4-qPCR-F	AGTGCCCGAAACCCACACTG
hOCT4-qPCR-R	ACCACACTCGGACCACATCCT
hNANOG-qPCR-F	TTTGTGGGCCTGAAGAAAACT
hNANOG-qPCR-R	AGGGCTGTCCTGAATAAGCAG
hSOX2-qPCR-F	CACTGCCCCTCTCACACATG
hSOX2-qPCR-R	TCCCATTTCCCTCGTTTTTCT
hATF4-qPCR-F	GCTAAGGCGGGCTCCTCCGA
hATF4-qPCR-R	ACCCAACAGGGCATCCAAGTCG
hTBXT-qPCR-F	CAGTGGCAGTCTCAGGTTAAGAAGGA
hTBXT-qPCR-R	CGCTACTGCAGGTGTGAGCAA
hEOMES-qPCR-F	CATGCAGGGCAACAAAATGTATG
hEOMES-qPCR-R	GTGTTGTTGTTATTTGCGCCTTTGT
hTBX6-qPCR-F	AGCCTGTGTCTTTCCATCGT
hTBX6-qPCR-R	GCTGCCCGAACTAGGTGTAT
hNKX2-5-qPCR-F	CAAGTGTGCGTCTGCCTTT
hNKX2-5-qPCR-R	CAGCTCTTTCTTTTCGGCTCTA
hISL1-qPCR-F	ATCAGGTTGTACGGGATCAAATG
hISL1-qPCR-R	ATGTGATACACCTTGGAGCG
hMYH6-qPCR-F	GCTGGTCACCAACAATCCCTA
hMYH6-qPCR-R	CGTCAAAGGCACTATCGGTGG
hMYL7-qPCR-F	ACATCATCACCCATGGAGACGAGA
hMYL7-qPCR-R	GCAACAGAGTTTATTGAGGTGCCC
hTNNT2-qPCR-F	TTCACCAAAGATCTGCTCCTCGCT
hTNNT2-qPCR-R	TTATTACTGGTGTGGAGTGGGTGTGG
hWARS1-qPCR-F	GTTTCCACGGACGCCCA
hWARS1-qPCR-R	GACGCATTTCCCGCTTTGAG
hHERPUD1-qPCR-F	CCGGTTACACACCCTATGGG
hHERPUD1-qPCR-R	TGAGGAGCAGCATTCTGATTG
hPSAT1-qPCR-F	TGCCGCACTCAGTGTTGTTAG
hPSAT1-qPCR-R	GCAATTCCCGCACAAGATTCT
hGAPDH-qPCR-F	AAGGTGAAGGTCGGAGTCAAC
hGAPDH-qPCR-R	GGGGTCATTGATGGCAACAATA
hACTB-qPCR-F	CCTGTACGCCAACACAGTGC
hACTB-qPCR-R	ATACTCCTGCTTGCTGATCC
hPERK-qPCR-F	AATGCCTGGGACGTGGTGGC
hPERK-qPCR-R	TGGTGGTGCTTCGAGCCAGG
hSCN5A-qPCR-F	AGCTGGCTGATGTGATGGTC
hSCN5A-qPCR-R	CACTTGTGCCTTAGGTTGCC
hKCNJ5-qPCR-F	GCTGGCGATTCTAGGAATGC
hKCNJ5-qPCR-R	TCTGTGGCAATGGGGACATAA

hKCNIP2-qPCR-F	ATGGGCAAGTACACGTACCCT	
hKCNIP2-qPCR-R	GTCACCACACCATCCTTGTTT	
hCACNB1-qPCR-F	GGCTACGAGGTTACAGACATGA	
hCACNB1-qPCR-R	CTGCCGTCACACGAGTGAT	
hCACNA1G-qPCR-F	ACACTTGGAACCGGCTTGAC	
hCACNA1G-qPCR-R	AGCACACGGACTGTCCTGA	
hCACNA1D-qPCR-F	GCTTCGGAACCGATGCTTC	
hCACNA1D-qPCR-R	TCCTCGTTCTCTGTCTGGTAAT	
hKCNJ3-qPCR-F	CCTGGCTTTTCATGGCGTC	
hKCNJ3-qPCR-R	GCAAGGCGTGTAGTTACCG	
hATP2A2-qPCR-F	CATCAAGCACACTGATCCCGT	
hATP2A2-qPCR-R	CCACTCCCATAGCTTTCCCAG	
hMYL2-qPCR-F	TTGGGCGAGTGAACGTGAAAA	
hMYL2-qPCR-R	CCGAACGTAATCAGCCTTCAG	
hPERK-Oligo siRNA	GCAUCUGCCUGGUUACUUATT	Oligo siRNA for knock down
